# Hydroquinone–pyrrole dyads with varied linkers

**DOI:** 10.3762/bjoc.12.10

**Published:** 2016-01-18

**Authors:** Hao Huang, Christoffer Karlsson, Maria Strømme, Martin Sjödin, Adolf Gogoll

**Affiliations:** 1 Nanotechnology and Functional Materials, Department of Engineering Sciences, The Ångström Laboratory, Uppsala University, Box 534, SE-751 21 Uppsala, Sweden; 2 Department of Chemistry - BMC, Biomedical Centre, Uppsala University, Box 576, SE-751 23 Uppsala, Sweden

**Keywords:** conjugation, heterocycles, hydroquinone, linker effect, pyrrole

## Abstract

A series of pyrroles functionalized in the 3-position with *p*-dimethoxybenzene via various linkers (CH_2_, CH_2_CH_2_, CH=CH, C≡C) has been synthesized. Their electronic properties have been deduced from ^1^H NMR, ^13^C NMR, and UV–vis spectra to detect possible interactions between the two aromatic subunits. The extent of conjugation between the subunits is largely controlled by the nature of the linker, with the largest conjugation found with the *trans*-ethene linker and the weakest with the aliphatic linkers. DFT calculations revealed substantial changes in the HOMO–LUMO gap that correlated with the extent of conjugation found experimentally. The results of this work are expected to open up for use of the investigated compounds as components of redox-active materials in sustainable, organic electrical energy storage devices.

## Introduction

**Figure 1:**
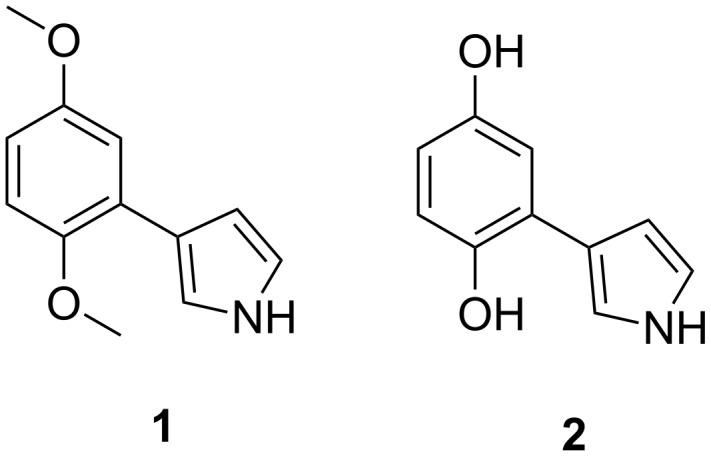
Structure of pyrrole/hydroquinone derivatives 3-(2,5-dimethoxyphenyl)-1*H*-pyrrole (**1**) and 3-(1,4-dihydroxyphenyl)-1*H*-pyrrole (**2**) [5].

Quinone–pyrrole dyads have attracted interest in various applications due to the possibility of modulating the electronic interaction between the two subunits, with porphyrin–quinone dyads being well-known examples [1–3]. Recently, we have shown the suitability of the quinone–hydroquinone redox couple (Figure 1) as the redox active and capacity carrying component in conducting redox polymers (CRPs) [4–5]. To further investigate the interaction between the molecular components in these systems, a series of compounds with different linkers between the pyrrole and hydroquinone subunits was designed [6]. Although it is known that N-protected pyrrole (Py) can be selectively functionalized at the β-position [7], we found the published procedures to be unsuitable for our purpose (vide infra). Therefore, we have developed improved procedures for the synthesis of quinone–pyrrole dyads with a variety of linkers between the two subunits.

**Figure 2:**
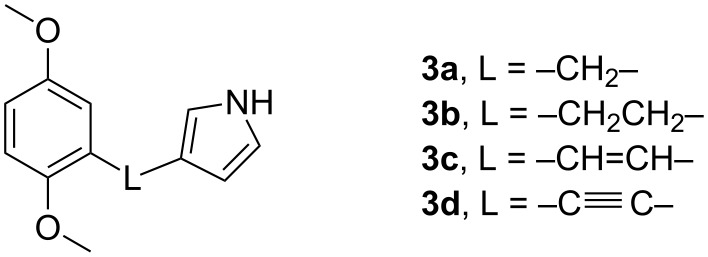
Hydroquinone dimethyl ether functionalized pyrroles with linkers L discussed in this study.

The target compounds thus consist of a pyrrole unit as well as a hydroquinone group (protected as dimethyl ether, i.e., dimethoxybenzene, DMB), connected by different linkers, resulting in varying distance and degree of conjugation between the two subunits (Figure 2). DMB was chosen due to its superior stability compared to the hydroquinone counterpart during the synthesis. The corresponding hydroquinone analogue can be achieved by straightforward demethylation with BBr_3_ [8–10]. The electronic properties of the monomers were investigated by NMR and UV–vis spectroscopy as well as density functional theory (DFT) calculations.

## Results and Discussion

### Synthesis

#### Methyl linker: Synthesis of 3-(2,5-dimethoxybenzyl)-1*H*-pyrrole (**3a**)

**Scheme 1:**
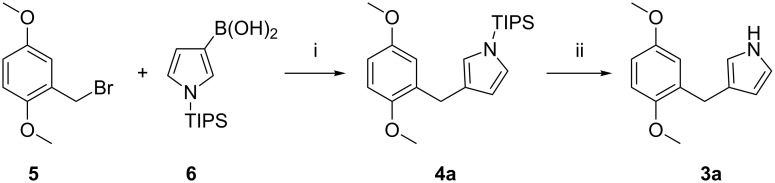
Synthetic route for 3-(2,5-dimethoxybenzyl)-1*H*-pyrrole (**3a**). Conditions: i) Pd(PPh_3_)_4_, Na_2_CO_3_ (2 M aq), MeOH/toluene, microwave heating, 110 °C, 4 h, 56%; ii) TBAF, THF, rt, 0.5 h, 88%.

For our target compound 3-(2,5-dimethoxybenzyl)-1*H*-pyrrole (**3a**), we initially considered some published procedures, but none of them were deemed adequate for our requirements. For example, Foos et al. have devised a synthetic route using alkylation of pyrrylmagnesium bromide with the benzyl bromide **5** [10], but yielding only 3.1% after a tedious work-up. Aquino-Binag et al. achieved a total yield of 70% [11], however, their procedure relies on a Wolff–Kishner reduction of the corresponding acylpyrrole with hydrazine hydrate, making it impractical in countries where the use of hydrazine has been restricted by law [12]. As an alternative, the Suzuki–Miyaura reaction can be utilized for C–C cross coupling of benzyl halides with heteroarylboronic acids. However, in contrast to its use for the synthesis of thiophene and furane derivatives, it has rarely been employed for the coupling of pyrrolylboronic acids with benzyl halides [13–17]. We applied the Suzuki–Miyaura cross-coupling as shown in Scheme 1, with a total yield of nearly 50% (from 2,5-dimethoxybenzyl bromide (**5**)).

Starting material **5** was prepared from 2,5-dimethoxybenzylalcohol by a reported procedure [18] in 95% yield. Using typical conditions, the yield of the coupling reaction was 56%, which was considered satisfactory because of the ready availability of starting materials and the modest reaction time of 4 hours. Since the starting materials in the Suzuki–Miyaura cross-coupling tolerate a wide variety of functional groups, facile and versatile combination of different dihydroxybenzyl halide derivatives and pyrrolylboronic acids should be possible.

#### Ethylene linker: Synthesis of 3-(2,5-dimethoxystyryl)-1*H*-pyrrole (**3c**)

Our initial approach of synthesizing vinyl linker dyad **3c** was to use a Heck reaction, coupling 3-iodo-1-(triisopropylsilyl)-1*H*-pyrrole and dimethoxystyrene. A variation of reaction parameters was investigated, involving the selection of base and solvent. However, none of the attempted conditions gave the desired product. Instead, desilylation of 3-iodo-1-(triisopropylsilyl)-1*H*-pyrrole was observed during the reaction, and some of the dimethoxystyrene could be recovered (for details, see Supporting Information File 1).

According to a study by Liu et al. [19], the protecting group for 3-iodo pyrrole is essential in the Heck reaction involving pyrrole. Based on this suggestion, 3-iodo-1-tosyl-1*H*-pyrrole was also tested but did not result in any product. It is worth to mention that in their study, ten equivalents of styrene were used to obtain 28% yield, indicating that the reaction conditions were not optimal, and therefore cannot be applied to other vinyl substrates such as dimethoxystyrene directly. A synthetic route via hydrogenation of the ethynyl linker compound **4d** was also investigated (vide infra), but did not produce satisfactory results.

Settambolo et al. [20] reported a synthetic route starting from an acylation of pyrrole by phenylacetyl chloride via a Friedel–Crafts reaction, followed by reduction by NaBH_4_, and elimination to give the vinylpyrrole product. When this strategy was applied to the reaction of (2,5-dimethoxyphenyl)acetyl chloride with 1-(triisopropylsilyl)-1*H*-pyrrole, nearly 50% yield was achieved in the first step, but requiring a reaction time of more than one day. Therefore, considering the total reaction time and availability of starting material, a Wittig reaction was chosen as an alternative.

**Scheme 2:**
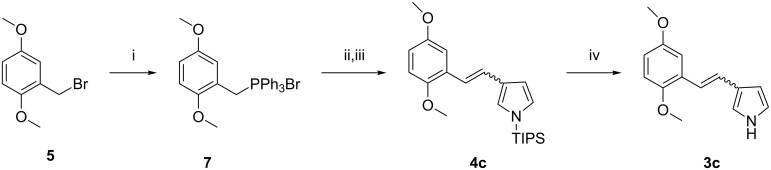
Synthetic route for 3-(2,5-dimethoxystyryl)-1*H*-pyrrole (**3c**); *cis*-**4c** and *trans*-**4c** were separated chromatographically to achieve the synthesis of both isomers of **3c** separately. Conditions: i) PPh_3_, toluene, reflux, overnight, quantitative; ii) *t*-BuOK, THF, 0.5 h, not isolated; iii) 1-(triisopropylsilyl)-1*H*-pyrrole-3-carbaldehyde, 80 °C, 3 h, 40%; iv) TBAF, THF, rt, 0.5 h, 86%.

The Wittig reaction approach is shown in Scheme 2. In this reaction, phosphonium salt **7** was prepared in quantitative yield from **5** and triphenylphosphine by reflux in toluene, according to a procedure reported for dimethoxybenzyl chloride [21]. The work-up involved simple filtration and washing with toluene. Use of *t*-BuOK as a base to deprotonate the phosphonium salt resulted in a dark red phosphorous ylide, to which 1-(triisopropylsilyl)-1*H*-pyrrole-3-carbaldehyde was added, followed by heating at 80 °C. The desired product **4c** was obtained in 40% yield after 3 h. When the reaction was performed at room temperature, only 20% yield was obtained after 48 h. Close to equal quantities of *cis*-**4c** and *trans*-**4c** were formed in this reaction, and were separated chromatographically. Having access to both isomers was desirable, since the stereoisomers are expected to have different electronic properties. After desilylation, compound **3c** was obtained in a combined total yield of 35% (sum of *cis*-**3c** and *trans*-**3c**) from the pyrrole-3-carbaldehyde. To obtain *trans*-**4c** selectively, a Wittig–Horner reaction was investigated, with dimethoxybenzyl bromide as starting material for the corresponding phosphonate. However, neither *n*-BuLi nor *t*-BuOK used as base succeeded to give the desired product.

#### Acetylene linker: Synthesis of 3-((2,5-dimethoxyphenyl)ethynyl)-1*H*-pyrrole (**3d**)

**Scheme 3:**
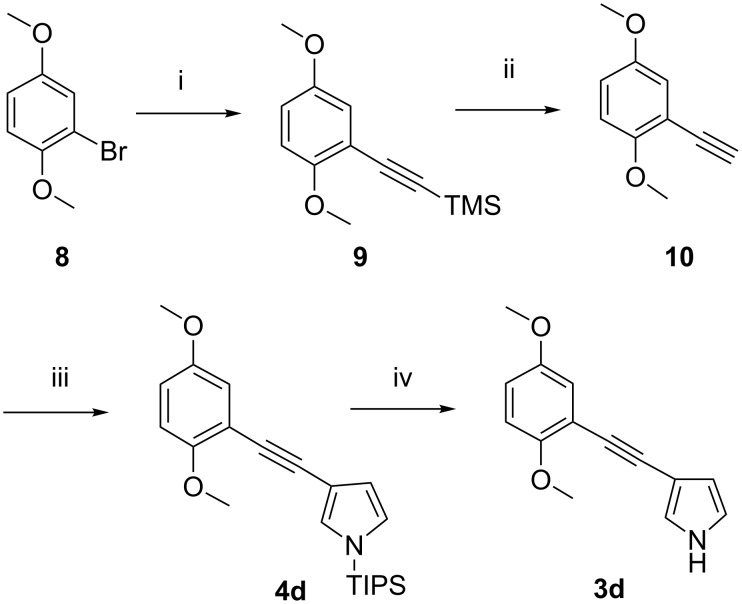
Synthesis of 3-((2,5-dimethoxyphenyl)ethynyl)-1*H*-pyrrole (**3d**). Conditions: i) Ethynyltrimethylsilane, Pd(PPh_3_)_2_Cl_2_, CuI, PPh_3_, diethylamine, DMF, microwave heating, 120 °C, 30 min, 95%; ii) NaOH (1 M, aq), MeOH/CHCl_3_, rt, 0.5 h, 90%; iii) Pd(PPh_3_)_2_Cl_2_, CuI, triethylamine, 3-iodo-1-(triisopropylsilyl)-1*H*-pyrrole, THF, rt, overnight, 50%; iv) TBAF, THF, rt, 0.5 h, 85%.

The synthesis strategy for the ethynyl-linked compound **3d** was straightforward, using a Sonogashira coupling reaction (Scheme 3). Thus, 2,5-dimethoxyphenyl bromide (**8**) was converted to ((2,5-dimethoxyphenyl)ethynyl)trimethylsilane (**9**), which was desilylated by addition of NaOH (aq) to give 2-ethynyl-1,4-dimethoxybenzene (**10**). Sonogashira conditions were as described by Erdélyi et al. using microwave heating [22], allowing the reaction to be completed in half an hour with 95% yield. A second Sonogashira reaction of **10** with 3-iodo-1-(triisopropylsilyl)-1*H*-pyrrole afforded the protected pyrrole derivative **4d**. For this second Sonogashira reaction, these conditions, when attempting to couple **10** to 3-bromo-1-(triisopropylsilyl)-1*H*-pyrrole resulted in low yield (20%) and also homocoupling. Considering that desilylation of triisopropylsilyl protected pyrrole might occur at high temperature in DMF, an overnight reaction at room temperature was performed, resulting in a yield for **4d** of 50%. In summary, after a four step synthesis route, the total yield of **3d** from **8** was 36%. Attempts were made to convert alkyne **4d** into the corresponding alkene **4c**. However, partial hydrogenations with a Lindlar catalyst [23] or with trimethylsilane using a palladium catalyst [24] were either unsuccessful or produced low yields (for details, see Supporting Information File 1).

#### Ethyl linker: Synthesis of 3-(2,5-dimethoxyphenethyl)-1*H*-pyrrole (**3b**)

**Scheme 4:**
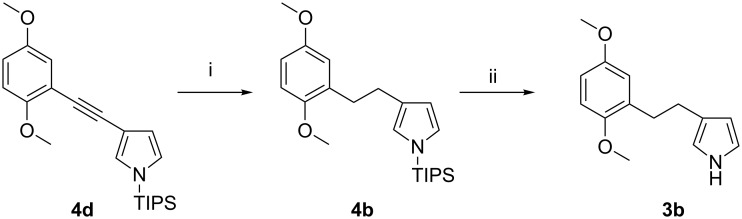
Synthesis of 3-(2,5-dimethoxyphenethyl)-1*H*-pyrrole (**3b**). Conditions: i) Pd/C, MeOH/acetone, rt, 1.5 h, 98%; ii) TBAF, THF, rt, 0.5 h, 85%.

Compound **3b** was prepared by reduction of **4d** with H_2_ over Pd/C catalyst (Scheme 4). The N-protected **4d** was used instead of **3d** to minimize the risk of decomposition during the hydrogenation reaction, since unprotected pyrrole is known to be far less stable [25]. After desilylation by TBAF, 3-(2,5-dimethoxyphenethyl)-1*H*-pyrrole (**3b**) was obtained in a total yield of 36% over five steps from **8**.

### Electronic properties: Evidence from NMR spectroscopy, UV–vis spectroscopy and DFT calculations

**Figure 3:**
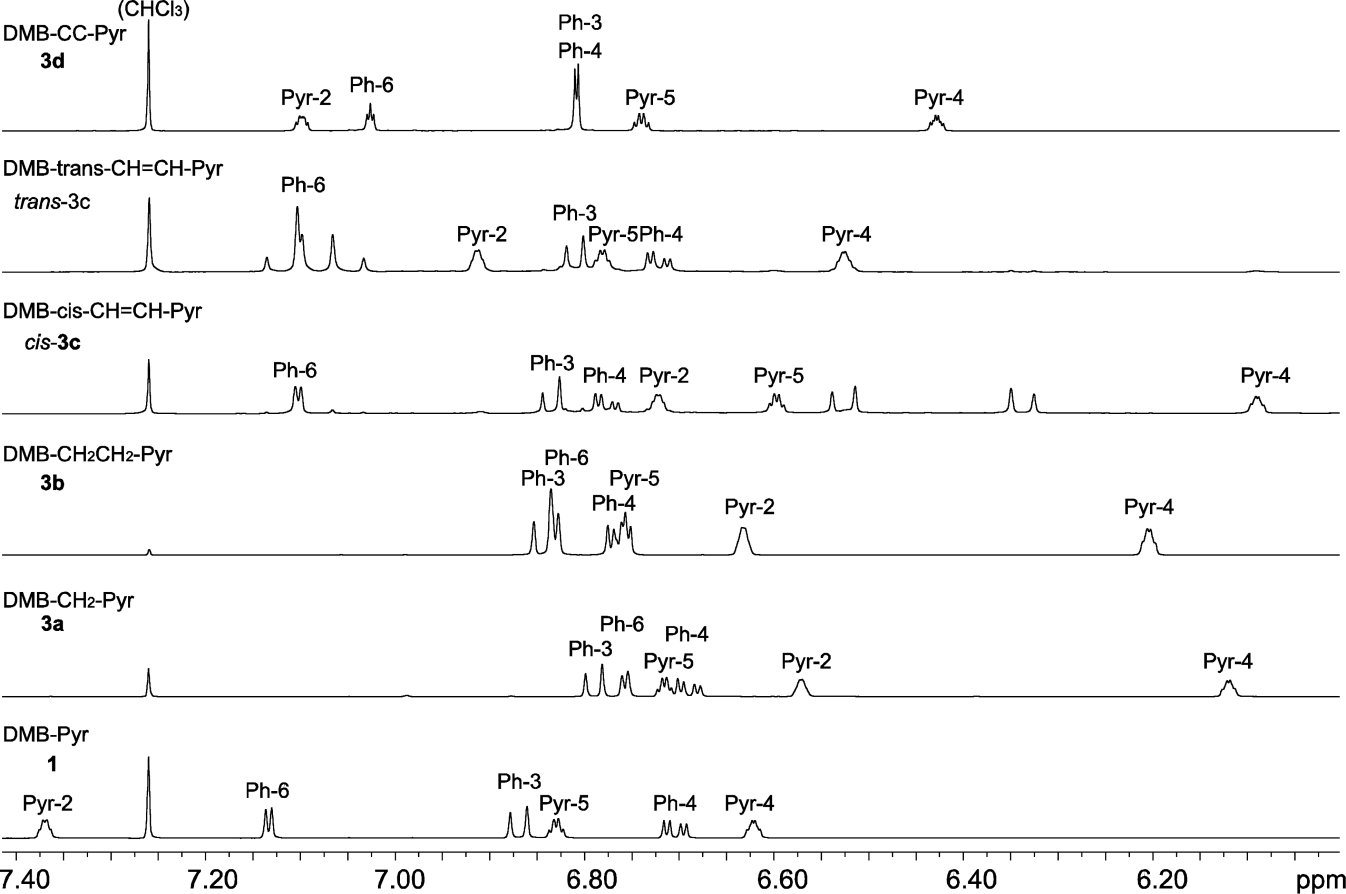
^1^H NMR spectra (400 MHz, CDCl_3_ solution) of the DMB-pyrrole dyads (aliphatic signals not shown).

**Figure 4:**
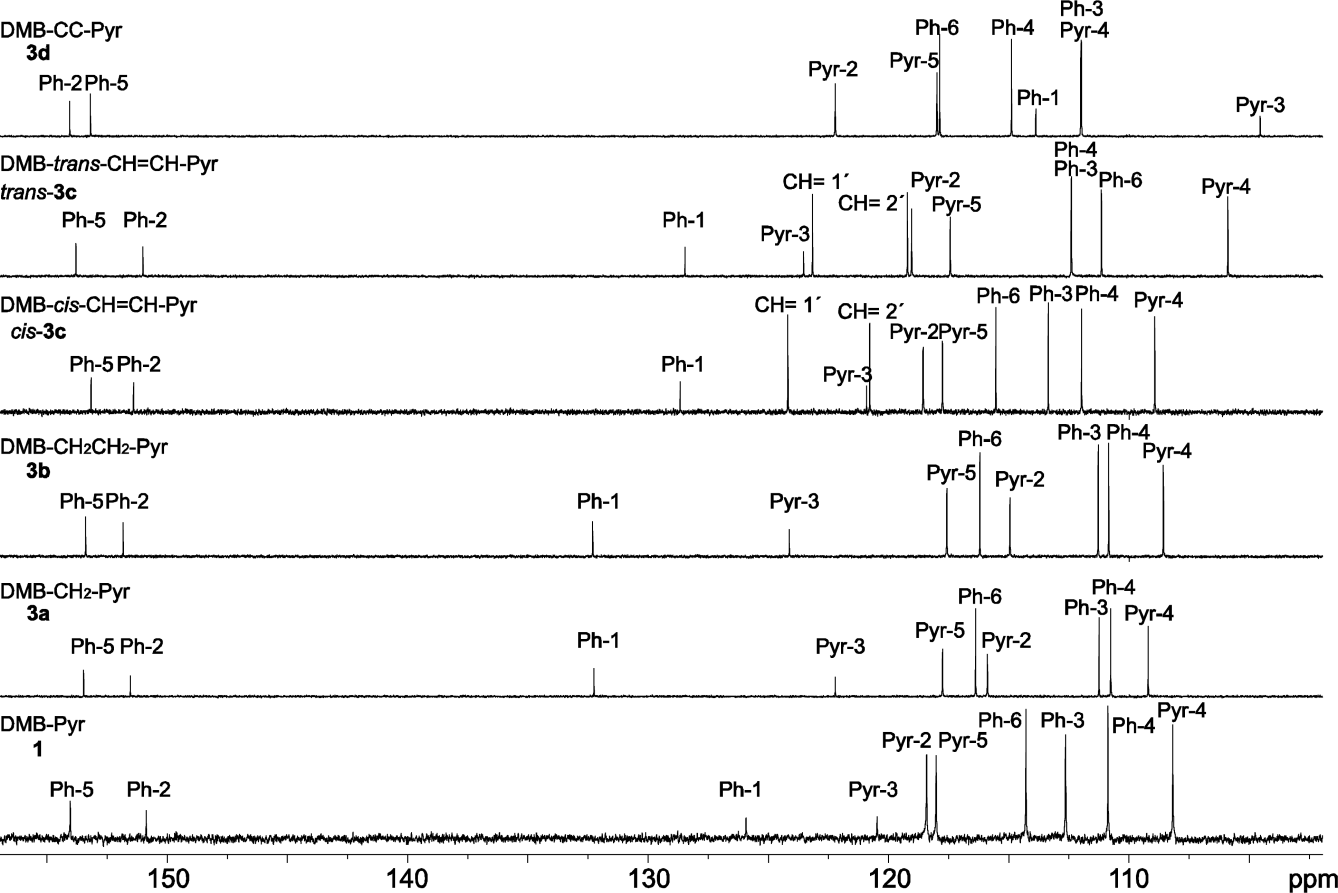
^13^C NMR spectra (100.6 MHz, CDCl_3_ solution) of the DMB-pyrrole dyads (aliphatic signals not shown).

In the NMR spectra, conjugation between the DMB and the pyrrole units is indicated by increased chemical shifts for DMB-H-6 (7.13–7.03 for **1**, **3c** and **3d**) as compared to the non-conjugated compounds (6.83–6.76 for **3a** and **3b**) (Figure 3). In particular, the chemical shift of the pyrrole H-2 (Pyr-2) is lower than that of the pyrrole H-5 (Pyr-5) in the non-conjugated compounds **3a** and **3b**, whereas pyrrole H-2 has considerably higher chemical shift than H-5 in the conjugated compounds **1**, **3c** and **3d**. This proton might therefore be used as an indicator of conjugation. Also, in the ^13^C NMR spectra, DMB C-1 (Ph-1) has a lower chemical shift in the conjugated compounds (128.7–113.9 for **1**, **3c** and **3d**) as compared to the non-conjugated ones (132.3 for **3a** and **3b**) (Figure 4).

To rationalize these chemical shift patterns, DFT calculations were performed. The observed ^1^H and ^13^C NMR chemical shifts are closely matched by those derived from the DFT calculations (see Supporting Information File 1 for details) [26–27], with average errors of 0.13 ppm for ^1^H (RMS error = 0.17), and 1.9 ppm for ^13^C (RMS error = 2.6). The above mentioned pattern of δ_pyrrole H-2_ > δ_pyrrole H-5_ for the conjugated compounds, with reversal for the non-conjugated compounds, was also present in the calculated chemical shifts, except for **3a** and *trans*-**3c**. We notice that in these two compounds, the deviation between observed and calculated chemical shift is untypically high at 0.23 ppm for both Py-5 in **3a** and Py-2 in *trans*-**3c**. This illustrates the well-known caveat for chemical shift predictions [26].

**Figure 5:**
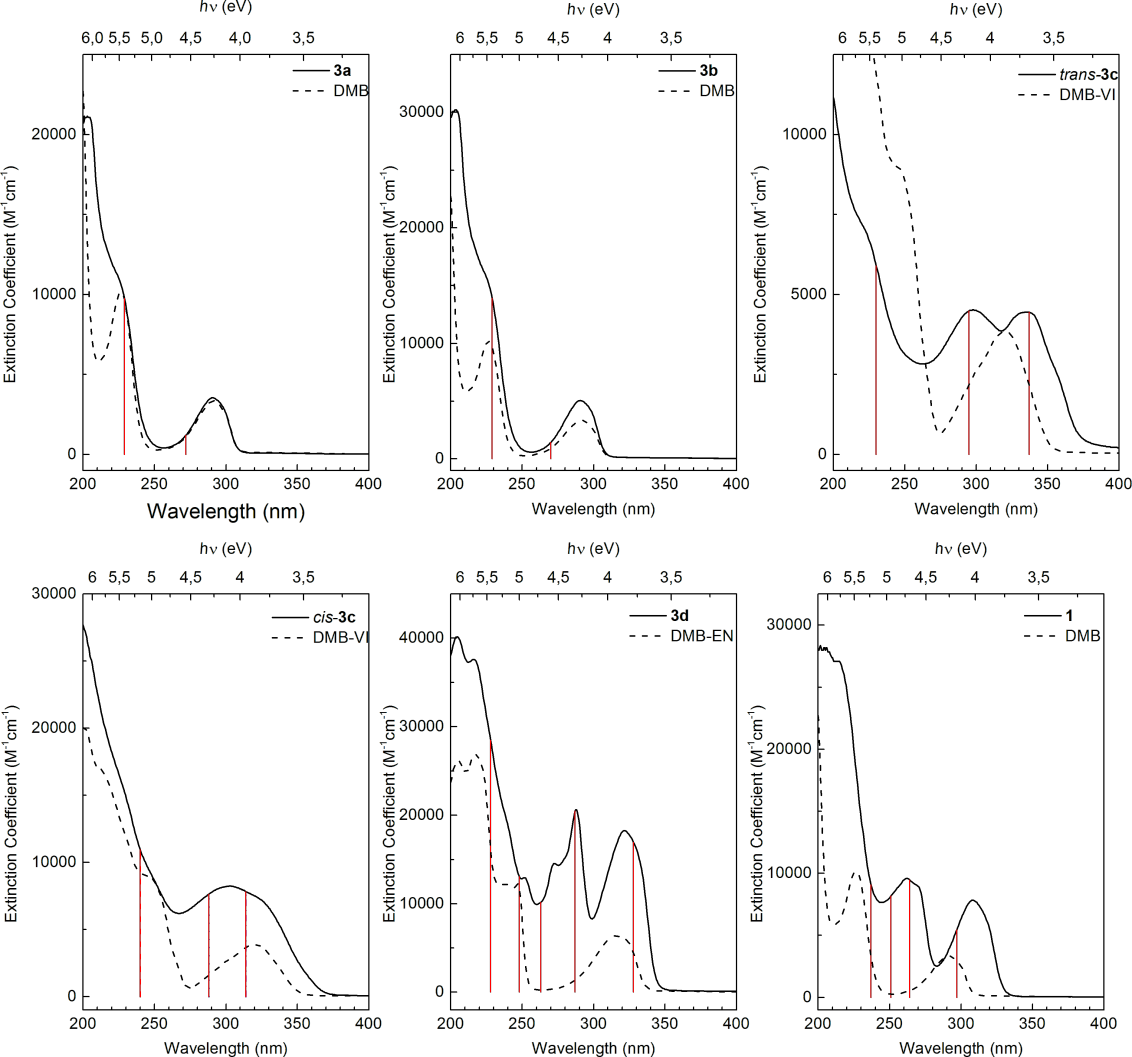
UV–vis absorption spectra of **1**, **3a**–**d**, (full lines) and the reference compounds DMB, DMB-VI, DMB-EN (dotted lines). The calculated wavelengths for absorption maxima from DFT calculations for each linker compound are indicated by vertical red lines.

To assess the electronic properties as indicated in UV–vis spectra, reference spectra of pyrrole, DMB, 1,4-dimethoxy-2-vinylbenzene (DMB-VI), and 1,4-dimethoxy-2-ethynylbenzene (DMB-EN) were recorded. The UV–vis spectra of non-conjugated **3a** and **3b** closely resemble the sum of absorptions from the individual DMB and pyrrole spectra, indicating the absence of substantial electronic effects from the substituent. On the other hand, for the conjugated **3c**, **3d** and **1**, a red shift is observed when compared to the individual reference compound spectra (Figure 5).

According to DFT calculations (see Supporting Information File 1 for details), the HOMO–LUMO transition is allowed for all compounds and has π–π* character. The HOMO of DMB couples strongly to the pyrrole HOMO, resulting in increased HOMO energy in **1**, *cis*-**3c**, *trans*-**3c,** and **3d**. Additionally, the corresponding coupling between the DMB and pyrrole LUMOs results in a lowering of the LUMO level in the combined molecular systems, since they are anti-bonding. The perturbation of the HOMOs and LUMOs depends on the degree of interaction between the two subunits, and is therefore a measure of the charge delocalization in the molecule. This effect can be viewed as an increased delocalized system that results in a lowering of the HOMO–LUMO gap, which can be traced by the red shifts of the highest wavelength absorption in UV–vis spectra (Table 1).

**Table 1:** Calculated and experimental values (nm) for the absorption maxima corresponding to the HOMO–LUMO transition in DMB, **1** and **3a**–**d**.

	DMB	**1**	**3a**	**3b**	*trans*-**3c**	*cis*-**3c**	**3d**

Calculation	270	297	270	272	337	314	328
Experimental	290	308	290	290	336	325	322

**Figure 6:**
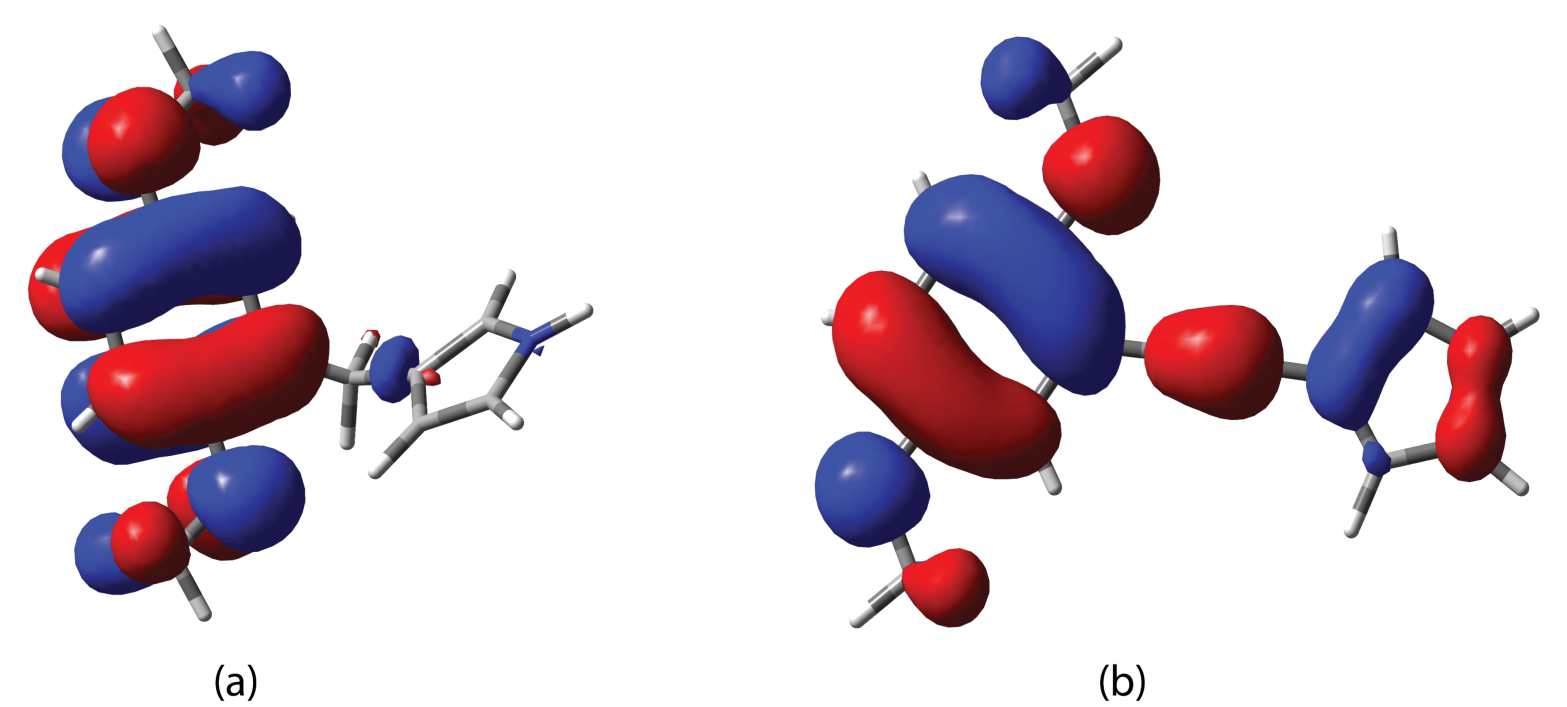
Calculated HOMO for **3a** (a) and **3d** (b).

The extent of conjugation among the series of linker compounds can be assessed by the HOMO–LUMO transition (Table 1). Since there is no conjugation between the two subunits in **3a** and **3b**, the HOMO and LUMO levels do not shift with respect to the reference compound DMB. This is also revealed by the electron distribution on both of the rings at the HOMO level. For both **3a** and **3b**, the HOMO is entirely localized at the DMB unit (Figure 6a), whereas for conjugated compounds such as **3d**, the HOMO is distributed over both rings (Figure 6b).

Among the conjugated compounds, **1** has the largest HOMO–LUMO gap. This may be explained by the smaller overall conjugated π system as compared to *cis*-**3c**, **3d** and *trans*-**3c**. The vinyl-linker structures (*cis*- and *trans*-**3c**) have more extended π systems, which yield lower energy HOMO–LUMO transitions. However, a significant difference is observed between *trans*-**3c** and *cis*-**3c**, which arises from the difference in their geometry: Whereas *trans*-**3c** has a planar structure, for *cis*-**3c** the pyrrole and DMB substituents on the vinyl linker were found to be twisted due to steric hindrance between them, which might also explain the low chemical shift of the pyrrole-H-4 in *cis*-**3c** as the result of being exposed to an anisotropy effect of the DMB ring.

## Conclusion

In summary, synthesis protocols for a series of methyl-protected hydroquinone-pyrrole dyads with good overall yields have been devised. DFT calculations allow insight into the electronic properties and conjugation within these compounds, with good agreement between calculations and experimental spectroscopic data. The extent of conjugation was found to vary with the linker, with *trans*-**3c** (*trans*-3-(2,5-dimethoxystyryl)-1*H*-pyrrole) having the strongest conjugation, as judged by the HOMO–LUMO gap, while **3a** (3-(2,5-dimethoxybenzyl)-1*H*-pyrrole) and **3b** (3-(2,5-dimethoxyphenethyl)-1*H*-pyrrole) have no conjugation, with HOMO–LUMO gaps similar to DMB (*p*-dimethoxybenzene). These compounds should be interesting as components of redox active materials, as we have recently shown in the synthesis of conducting redox polymers for use as the active material in organic electrical energy storage [6].

## Supporting Information

File 1 Synthetic details.

File 2 Optimization of experimental conditions, computated electron distributions, UV–vis, IR, and NMR spectral data.
