# Supplementary material for: Hydroquinone–pyrrole dyads with varied linkers
Source: Beilstein J Org Chem. 2016 Jan 18;12:89–96. doi: 10.3762/bjoc.12.10 (PMC4734300; doi:10.3762/bjoc.12.10)
Supplement: File 1 — Synthetic details. [file Beilstein_J_Org_Chem-12-89-s001.pdf]

Supporting Information

for

Hydroquinone–pyrrole dyads with varied linkers

Hao Huang[1], Christoffer Karlsson[1], Maria Strømme[1], Martin Sjödin[1] and Adolf Gogoll*,[2]

Address: [1] Nanotechnology and Functional Materials, Department of Engineering Sciences, The Ångström Laboratory, Uppsala University, Box 534, SE-751 21 Uppsala, Sweden and [2] Department of Chemistry - BMC, Biomedical Centre, Uppsala University, Box 576, SE-751 23 Uppsala, Sweden

Email: Adolf Gogoll - adolf.gogoll@kemi.uu.se

*Corresponding author

Synthetic details

Experimental

General information: All reagents were purchased from Sigma-Aldrich except boronic acid (purchased from Frontier Scientific, Inc., purity was quantified to be nearly 75%) and were used without further purification. Tetrahydrofuran (THF) and toluene were dried using a PureSolv PS-MD-4-EN solvent purification system and stored under argon atmosphere. Microwave-accelerated reactions were conducted under argon atmosphere in heavy-walled glass Smith process vials sealed with aluminum crimp caps fitted with a silicon septum. The microwave heating was

perfomed in a Smith Synthesizer single-mode microwave cavity producing continuous irradiation at 2450 MHz. Flash chromatography was either performed using VWR Normasil 60 silica gel (40–63 µm, 60 Å) or on a Grace REVELERIS® X2 Flash Chromatography System using Reveleris® high resolution flash cartridges. Analytical thin layer chromatography was performed using pre-coated Merck Silica 60 F254 plates, and compound visualization was achieved with UV light (254 nm). Room temperature (rt) refers to 20–23 °C. NMR spectra were recorded on an Agilent 400-MR spectrometer (^{1}H at 399.97 MHz, ^{13}C at 100.58 MHz). Chemical shifts are reported using the chloroform signal as an indirect reference to TMS (δ_H = 7.26 ppm, δ_C = 77.0 ppm). Coupling constants (*J*) are reported in Hz. Signal assignments are based on gHSQC [1], gHMBC [2], and NOESY [3,4] spectra. IR spectra were recorded for neat compounds on a Perkin Elmer Spectrum 100 FTIR spectrometer with UATR accessory. Melting points were measured in open capillaries using a Stuart Scientific SMP10 melting-point apparatus and are uncorrected. Mass spectra were recorded on a Thermo Finnigan GCQ mass spectrometer with direct inlet interface and electron ionization (70 eV). UV–vis spectra were recorded on a Shimadzu UV-1650PC spectrometer using 10 mm quartz cuvettes at RT with MeCN as solvent. HRMS were acquired using a Thermo Scientific LTQ Orbitrap Velos apparatus in infusion mode. NMR and IR spectra can be found in Supporting Information File 2.

3-(2,5-Dimethoxybenzyl)-1-(triisopropylsilyl)pyrrole (4a):

1-(Triisopropylsilyl)pyrrol-3-ylboronic acid (0.52 g, 1.5 mmol), 2,5-dimethoxybenzyl bromide (0.3 g, 1.3 mmol) and $Pd(PPh_3)_4$ (0.075 g, 0.07 mmol) were dissolved in a toluene/methanol mixture (8 mL, 1:1) that was pre-purged with argon in a heavy-wall

Smith process vial for 5 minutes, and then 2 M Na_2CO_3 (0.65 mL, 1.3 mmol) was added. The resulting solution was stirred under an argon atmosphere at 110 °C for 4 h in a microwave cavity. The reaction mixture was then poured into ice-water (20 mL) and extracted three times with ethyl acetate (10 mL). The combined organic phases were washed with brine and dried over $MgSO_4$. The solvent was evaporated under reduced pressure and a black crude oil was obtained. The crude product was purified on a silica column using 2.5% diethyl ether in pentane to give the pure product **4a** (0.27g, 0.73 mmol) as white solid in 56% yield. ^{1}H NMR: (400 MHz, $CDCl_3$) δ_H = 6.78 (1H, d, *J* = 8.3, Ph-H-3), 6.73 (1H, d, *J* = 3.0, Ph-H-6), 6.71 (1H, m, Py-H-2), 6.69 (1H, dd, *J* =3.0, 8.3, Ph-H-4), 6.58 (1H, m, Py-H-5), 6.17 (1H, m, Py-H-4), 3.83 (2H, s, -CH_2-), 3.79 (3H, s, OCH_3), 3.69 (3H, s, OCH_3), 1.43 (3H, sept, *J* = 7.6, TIPS-CH), 1.10 (18H, d, *J* = 7.6, TIPS-CH_3); ^{13}C NMR (100.6 MHz, $CDCl_3$) δ_C =153.5, 151.6, 132.6, 124.2, 123.7, 122.4, 115.7, 111.5, 111.3, 111.1, 56.1, 55.5, 27.3, 17.9, 11.7; mp 50-51 °C; IR (neat): 2948, 2867, 1499, 1210, 1092, 1045 cm^{-1}; UV–vis (MeCN, λ_{max}): 220, 290 nm; MS (ESI, 30 eV), *m/z (%)*: 373.0 (100); HRMS, calcd. for $C_{22}H_{36}NO_2Si$: *m/z* = 374.2510 $[M + H]^+$; found 374.2475.

3-((2,5-Dimethoxyphenyl)ethynyl)-1-(triisopropylsilyl)pyrrole (4d): Precursors ((2,5-dimethoxyphenyl)ethynyl)trimethylsilane (**9**) and 2-ethynyl-1,4-dimethoxybenzene (**10**) were synthesized according to literature procedures [5,6].To a solution of 3-iodo-1-(triisopropylsilyl)pyrrole (0.35 g, 1 mmol) in triethylamine (5 mL) was added $Pd(PPh_3)_2Cl_2$ (0.014 g, 0.02 mmol), and CuI (0.002 g, 0.01 mmol). A solution of **10** (0.16 g, 1 mmol) in THF (5 mL) was added to the suspension and stirred for 3 h under an argon atmosphere. The reaction mixture was concentrated under reduced pressure and the resulting residue was purified on a silica column

using gradient elution (5% to 20% diethyl ether in pentane) to give pure product **4d** (0.19 g, 0.5 mmol) as yellow soft solid in 50% yield. ^{1}H NMR: (400 MHz, $CDCl_3$) δ_H = 7.08 (1H, dd, *J* = 1.3, 2.2, Py-H-2), 7.03 (1H, dd, *J* = 1.7, 2.0, Ph-H-6), 6.80 (2H, m, Ph-H-3, Ph-H-4), 6.69 (1H, dd, *J* =2.2, 2.7, Py-H-5), 6.48 (1H, dd, *J* =1.3, 2.7, Py-H-4), 3.86 (3H, s, OCH_3), 3.77 (3H, s, OCH_3), 1.44 (3H, sept, *J* = 7.6, TIPS-CH), 1.10 (18H, d, *J* = 7.6, TIPS-CH_3) ppm; ^{13}C NMR (100.6 MHz, $CDCl_3$) δ_C =154.0, 153.2, 128.6, 124.1, 117.8, 114.8, 114.0, 113.9, 111.8, 106.3, 89.5, 84.1, 56.5, 55.8, 17.7, 11.6; mp 71-72 °C; IR (neat): 2952, 2866, 2216, 1499, 1210, 1083, 1043 cm^{-1}; UV–vis (MeCN, λ_{max}): 219, 237, 253, 273, 290, 322 nm; MS (ESI, 30 eV), *m*/*z (%)*: 383.0 (100); HRMS, calcd. for $C_{23}H_{34}NO_2Si$: *m*/*z* = 384.2353 [M + H]$^+$; found 384.2308.

3-(2,5-Dimethoxyphenethyl)-1-(triisopropylsilyl)pyrrole (4b): To a 100 mL round bottom flask, methanol (50 mL) was added, and degassed by switching between vacuum and Ar gas at least 5 times, and then Pd (10% on active carbon, 83 mg, 0.078 mmol) was added. The flask was connected to a hydrogen reservoir (1 atm) and a solution of **4d** (0.3 g, 0.78 mmol) in acetone (5 mL) was added. The reaction mixture was stirred at room temperature overnight and then the solid residue was filtered off by passing the mixture through a celite plug (5 cm) using acetone as eluent. The crude product was purified on a silica column using 5% Et_2O in pentane as eluent, resulting in product **4b** (298 mg, 0.76 mmol) as colorless oil in 98% yield. ^{1}H NMR: (400 MHz, $CDCl_3$) δ_H = 6.77 (1H, d, *J* = 8.6, Ph-H-3), 6.71 (1H, d, *J* = 3.0, Ph-H-6), 6.69 (1H, m, Py-H-2), 6.67 (1H, dd, *J* =3.0, 8.3, Ph-H-4), 6.50 (1H, m, Py-H-5), 6.19 (1H, m, Py-H-4), 3.78 (3H, s, OCH_3), 3.73 (3H, s, OCH_3), 2.87 (2H, m, -CH_2-), 2.78 (2H, m, -CH_2-), 1.41 (3H, sept, *J* = 7.6, TIPS-CH), 1.08 (18H, d, *J* = 7.6, TIPS-CH_3) ppm; ^{13}C NMR (100.6 MHz, $CDCl_3$) δ_C =153.4, 151.8, 132.4, 125.8,

123.9, 121.2, 116.3, 111.3, 110.8, 110.7, 56.0, 55.6, 31.8, 27.3, 17.9, 11.7; IR (neat): 2945, 2867, 1499, 1210, 1097, 1050 cm^{-1}; UV–vis (MeCN, λ_{max}): 223, 290 nm; MS (ESI, 30 eV), *m/z (%)*: 386.7 (30), 236.0 (100); HRMS, calcd. for $C_{23}H_{38}NO_2Si$: *m*/*z* = 388.2666 $[M + H]^+$; found 388.2628.

3-(2,5-Dimethoxystyryl)-1-(triisopropylsilyl)-1*H*-pyrrole (4c): To a heavy-walled Smith process vial (25 mL), (2,5-dimethoxybenzyl)triphenylphosphonium bromide [7] (1.09 g, 2.2 mmol) and potassium *tert*-butoxide (0.26 g, 2.3 mmol) were added, followed by addition of THF (10 mL). The reaction mixture was stirred at rt for 1 hour and then a solution of 1-(triisopropylsilyl)-1*H*-pyrrole-3-carbaldehyde [8] (0.5 g, 2.0 mmol) in THF (5 mL) was added via cannula. The resulting reaction mixture was heated to 80 °C and stirred for another 3 hours. After the reaction completed, the reaction mixture was concentrated under reduced pressure and the resulting residue was dissolved in acetone. Undissolved solid was removed by filtration and the filtrate was concentrated. This crude product was purified on a Grace REVELERIS® X2 Flash Chromatography System using a Reveleris® high resolution flash cartridge (40 g silica) with gradient from pentane to 4% Et_2O in pentane. Repeating the purification 4 times afforded the separate *cis* and *trans* isomers in a combined yield of 0.31 g (0.8 mmol, 40%).

***trans*-3-(2,5-Dimethoxystyryl)-1-(triisopropylsilyl)-1*H*-pyrrole (*trans*-3c)** was obtained as yellow oil (0.16 g, 0.41 mmol): ^{1}H NMR: (400 MHz, $CDCl_3$) δ_H = 7.12 (1H, d, J = 16.8, CH=CH), 7.11 (1H, d, *J* = 3.0, Ph-H-6), 7.07 (1H, d, *J* = 16.8, CH=CH), 6.88 (1H, m, Py-H-2), 6.81 (1H, d, *J* =8.9, Ph-H-3), 6.77 (1H, m, Py-H-5), 6.72 (1H, dd, *J* =3.0, 8.9, Ph-H-4) 6.62 (1H, m, Py-H-4), 3.83 (3H, s, OCH_3), 3.81 (3H, s,

OCH_3), 1.47 (3H, sept, *J* = 7.6, TIPS-CH), 1.12 (18H, d, *J* = 7.6, TIPS-CH_3) ppm; ^{13}C NMR (100.6 MHz, $CDCl_3$) δ_C =153.8, 151.0, 128.7, 125.6, 125.5, 124.1, 123.5, 119.0, 112.4, 112.3, 111.1, 107.8, 56.4, 55.7, 17.8, 11.7; IR (neat): 2944, 2867, 1634, 1604, 1492, 1230, 1219, 1090 cm^{-1}; UV–vis (MeCN, λ_{max}) 224, 306, 335; MS (ESI, 30 eV), *m/z (%)*: 385.0 (100); HRMS, calcd. for $C_{23}H_{36}NO_2Si$: *m/z* = 386.2510 $[M + H]^+$; found 386.2466.

***cis*-3-(2,5-Dimethoxystyryl)-1-(triisopropylsilyl)-1*H*-pyrrole (*cis*-4c)** was obtained as colorless oil (0.15 g, 0.39 mmol): ^1H NMR: (400 MHz, $CDCl_3$) δ_H = 7.09 (1H, d, *J* = 2.9, Ph-H-6), 6.83 (1H, d, *J* = 9.0, Ph-H-3), 6.76 (1H, dd, *J* = 2.9, 9.0, Ph-H-4), 6.64 (1H, m, Py-H-5), 6.54 (1H, d, *J* = 11.8, CH=CH), 6.53 (1H, m, Py-H-2), 6.32 (1H, d, *J* = 11.8, CH=CH), 6.16 (1H, dd, *J* = 1.0, 2.2, Py-H-4), 3.77 (3H, s, OCH_3), 3.69 (3H, s, OCH_3), 1.36 (3H, sept, *J* = 7.6, TIPS-CH), 1.05 (18H, d, *J* = 7.6, TIPS-CH_3) ppm; ^{13}C NMR (100.6 MHz, $CDCl_3$) δ_C =153.2, 151.4, 128.9, 125.3, 124.6, 124.1, 122.9, 120.5, 115.6, 113.2, 111.9, 110.9, 56.4, 55.7, 17.8, 11.6; IR (neat): 2945, 2867,1633, 1604, 1492, 1090; UV–vis (MeCN, λ_{max}) 301, 325; MS (ESI, 30 eV), *m/z (%)*: 384.9 (100); HRMS, calcd. for $C_{23}H_{36}NO_2Si$: *m/z* = 386.2510 $[M + H]^+$; found 386.2468.

General procedure for desilylation: A 1.0 M solution of TBAF in THF (1 equiv) was added to a solution of the silylated compounds (1 equiv) in THF. The reaction mixture was stirred at room temperature for 30 minutes and then the solvent was removed under reduced pressure yielding a crude product. The crude product was purified on a silica column using 20% Et_2O in pentane as eluent to give the pure product.

3-(2,5-Dimethoxybenzyl)-1*H*-pyrrole (3a) was obtained as yellow oil (0.077 g, 0.35 mmol from 0.15 g **4a**, 88%) ^{1}H NMR: (400 MHz, $CDCl_3$) δ_H = 8.02 (1H, bs, NH), 6.79 (1H, d, *J* = 8.6, Ph-H-3), 6.76 (1H, d, *J* = 3.0, Ph-H-6), 6.72 (1H, dd, *J* = 2.4, 4.7, Py-H-5), 6.69 (1H, dd, *J* =3.0, 8.6, Ph-H-4), 6.57 (1H, m, Py-H-2), 6.12 (1H, dd, *J* = 2.4, 3.9, Py-H-4), 3.82 (2H, s, CH_2), 3.81 (3H, s, OCH_3-2), 3.73 (3H, s, OCH_3-5); ^{13}C NMR (100.6 MHz, $CDCl_3$) δ_C = 153.5 (Ph-5), 151.5 (Ph-2), 132.2 (Ph-1), 122.2 (Py-3), 117.7 (Py-5), 116.4 (Ph-6), 116.0 (Py-2), 111.2 (Ph-3), 110.7 (Ph-4), 109.2 (Py-4), 56.1 (OCH_3-2), 55.6 (OCH_3-5), 27.3 (CH_2); IR (neat): 3395, 2996, 2938, 2833, 1497, 1210, 1043, 1024 cm^{-1}; UV–vis (MeCN, λ_{max}): 220, 290 nm; MS (ESI, 30 eV), *m*/*z (%)*: 216.9 (100); HRMS, calcd. for $C_{13}H_{16}NO_2$: *m*/*z* = 218.1176 [M + H]$^+$; found 218.1154.

3-((2,5-Dimethoxyphenyl)ethynyl)-1*H*-pyrrole (3d) was obtained as yellow solid (0.1 g, 0.44 mmol from 0.2 g **4d**, 85%). ^{1}H NMR: (400 MHz, $CDCl_3$) δ_H = 8.32 (1H, bs, NH) 7.08 (1H, m, Py-H-2), 7.03 (1H, dd, *J* = 1.7, 2.0, Ph-H-6), 6.80 (2H, m, Ph-H-3, Ph-H-4), 6.73 (1H, dd, *J* =2.4, 4.8, Py-H-5), 6.42 (1H, m, Py-H-4), 3.86 (3H, s, OCH_3-2), 3.78 (3H, s, OCH_3-5) ppm; ^{13}C NMR (100.6 MHz, $CDCl_3$) δ_C = 154.1 (Ph-2), 153.2 (Ph-5), 122.2 (Py-2), 118.0 (Py-5), 117.9 (Ph-6), 114.9 (Ph-4), 113.9 (Ph-1), 112.0 (Ph-3), 112.0 (Py-4), 104.6 (Py-3), 89.5 (C≡C-1´), 84.1 (C≡C-2´), 56.5 (OCH_3-2), 55.8 (OCH_3-5); mp 128-129 °C; IR (neat): 3366, 2953, 2865, 2208, 1582, 1497, 1210, 1043, 1013 cm^{-1}; UV–vis (MeCN, λ_{max}): 217, 237, 252, 273, 288, 322 nm; MS (ESI, 30 eV), *m*/*z (%)*: 226.9 (100); HRMS, calcd. for $C_{14}H_{14}NO_2$: *m*/*z* = 228.1019 [M + H]$^+$; found 228.0997.

3-(2,5-Dimethoxyphenethyl)-1*H*-pyrrole (3b) was obtained as colorless oil (0.048 g, 0.2 mmol from 0.1 g **4b**, 80%). ^{1}H NMR: (400 MHz, $CDCl_3$) δ_H = 8.02 (1H, bs, NH) 6.84 (1H, d, *J* = 8.7, Ph-H-3), 6.84 (1H, d, *J* = 3.1, Ph-H-6), 6.76 (1H, dd, *J* = 2.6, 4.8, Py-H-5), 6.76 (1H, dd, *J* =3.1, 8.7, Ph-H-4), 6.63 (1H, m, Py-H-2), 6.20 (1H, m, Py-H-4), 3.79 (3H, s, OCH_3-2), 3.76 (3H, s, OCH_3-5), 2.87 (2H, m, CH_2-2´), 2.78 (2H, m, CH_2-1´); ^{13}C NMR (100.6 MHz, $CDCl_3$) δ_C = 153.4 (Ph-5), 151.9 (Ph-2), 132.3 (Ph-1), 124.1 (Py-3), 117.6 (Py-5), 116.2 (Ph-6), 115.0 (Py-2), 111.3 (Ph-3), 110.9 (Ph-4), 108.6 (Py-4), 56.0 (OCH_3-2), 55.7 (OCH_3-5), 32.0 (CH_2-2´), 27.3 (CH_2-1´); IR (neat): 3394, 2934, 2833, 1497, 1210, 1044 cm^{-1}; UV–vis (MeCN, λ_{max}): 222, 290 nm; MS (ESI, 30 eV), *m*/*z (%)*: 231.0 (100), 200.0 (75); HRMS, calcd. for $C_{14}H_{18}NO_2$: *m*/*z* = 232.1331 $[M + H]^+$; found 232.1308.

***trans*-3-(2,5-Dimethoxystyryl)-1*H*-pyrrole (*trans*-3c)** was obtained as oil (0.052 g, 0.22 mmol from 0.1 g *trans*-**4c**, 88%). ^{1}H NMR: (400 MHz, $CDCl_3$) δ_H = 8.18 (1H, bs, NH), 7.13 (1H, d, *J* = 16.5, CH=CH-2´), 7.10 (1H, d, *J* = 3.0, Ph-H-6), 7.05 (1H, d, *J* = 16.5, CH=CH-1´), 6.91 (1H, m, Py-H-2), 6.81 (1H, d, *J* = 9.1, Ph-H-3), 6.78 (1H, m, Py-H-5), 6.72 (1H, dd, *J* =3.0, 9.1, Ph-H-4) 6.52 (1H, m, Py-H-4), 3.83 (3H, s, OCH_3-2), 3.81 (3H, s, OCH_3-5) ppm; ^{13}C NMR (100.6 MHz, $CDCl_3$) δ_C = 153.8 (Ph-5), 151.0 (Ph-2), 128.5 (Ph-1), 123.5 (Py-3), 123.1 (CH=CH-1´), 119.2 (CH=CH-2´), 119.1 (Py-2), 117.4 (Py-5), 112.4 (Ph-4), 112.4 (Ph-3), 111.2 (Ph-6), 105.9 (Py-4), 56.4 (OCH_3-2), 55.7 (OCH_3-5); IR (neat): 3400, 2996, 2938, 2833, 1687, 1489, 1220, 1023; UV–vis (MeCN, λ_{max}): 222, 298, 336 cm^{-1}; MS (ESI, 30 eV), *m*/*z (%)*: 229.0 (100), 198 (85); HRMS, calcd. for $C_{14}H_{16}NO_2$: *m*/*z* = 230.1176 $[M + H]^+$; found 230.1153.

***cis*-3-(2,5-Dimethoxystyryl)-1*H*-pyrrole (*cis*-3c)** was obtained as oil (0.051 g, 0.22 mmol from 0.1 g *cis*-**4c**, 85%). ^{1}H NMR: (400 MHz, $CDCl_3$) δ_H = 8.05 (1H, bs, NH), 7.10 (1H, d, *J* = 3.1, Ph-H-6), 6.84 (1H, d, *J* = 8.9, Ph-H-3), 6.78 (1H, dd, *J* = 3.1, 8.9, Ph-H-4), 6.72 (1H, m, Py-H-2), 6.60 (1H, m, Py-H-5), 6.53 (1H, d, *J* = 11.9, CH=CH-1´), 6.34 (1H, d, *J* = 11.9, CH=CH-2´), 6.09 (1H, m, Py-H-4), 3.78 (3H, s, OCH_3-2), 3.70 (3H, s, OCH_3-5) ppm; ^{13}C NMR (100.6 MHz, $CDCl_3$) δ_C = 153.2 (Ph-5), 151.4 (Ph-2), 128.7 (Ph-1), 124.2 (CH=CH-1´), 120.9 (Py-3), 120.8 (CH=CH-2´), 118.6 (Py-2), 117.8 (Py-5), 115.6 (Ph-6), 113.4 (Ph-3), 112.0 (Ph-4), 108.9 (Py-4), 56.3 (OCH_3-2), 55.8 (OCH_3-5); IR (neat): 3389, 2998, 2938, 2832, 1634, 1489, 1220, 1040; UV–vis (MeCN, λ_{max}): 230, 297, 325 cm^{-1}; MS (ESI, 30 eV), *m*/*z (%)*: 229.0 (100), 198 (91); HRMS, calcd. for $C_{14}H_{16}NO_2$: *m*/*z* = 230.1176 [M + H]$^+$; found 230.1152.

Density Functional Theory Calculation

Gaussian 09 [9] as used to perform the DFT calculations with the 6-311+G(d) basis set at the B3LYP level of theory, which has been previously used for similar structures with good results [10,11]. Structures were optimized in the gas phase and solvated using PCM solvation model in MeCN solution with the UAKS topological method. UV–vis spectra were calculated by performing time dependent (TD-SCF) energy calculations for the twelve lowest energy levels of optimized and solvated structures. MO isosurfaces were rendered with GaussView.

References

1. Davis, A. L.; Keeler, J.; Laue, E. D.; Moskau, D., *J. Magn. Reson.* **1992**, *98*, 207-216.
2. Hurd, R. E.; John, B. K., *J. Magn. Reson.* **1991**, *91*, 648-653.

3. Kumar, A.; Ernst, R. R.; Wüthrich, K., *Biochem. Biophys. Res. Commun.* **1980**, 95, 1-6.
4. Bodenhausen, G.; Kogler, H.; Ernst, R. R., *J. Magn. Reson.* **1984**, *58*, 370-388.
5. M. Beccalli, E.; Marchesini, A.; Pilati, T., *Tetrahedron* **1994**, *50*, 12697-12712.
6. Yamaguchi, Y.; Matsubara, Y.; Ochi, T.; Wakamiya, T.; Yoshida, Z.-i., *J. Am. Chem. Soc.* **2008**, *130*, 13867-13869.
7. Rosowsky, A.; Papoulis, A. T.; Forsch, R. A.; Queener, S. F., *J. Med. Chem.* **1999**, 42, 1007-1017.
8. Bray, B. L.; Mathies, P. H.; Naef, R.; Solas, D. R.; Tidwell, T. T.; Artis, D. R.; Muchowski, J. M., *J. Org. Chem.* **1990**, *55*, 6317-6328.
9. Frisch, M. J.; Trucks, G. W.; Schlegel, H. B.; Scuseria, G. E.; Robb, M. A.; Cheeseman, J. R.; Scalmani, G.; Barone, V.; Mennucci, B.; Petersson, G. A.; Nakatsuji, H.; Caricato, M.; Li, X.; Hratchian, H. P.; Izmaylov, A. F.; Bloino, J.; Zheng, G.; Sonnenberg, J. L.; Hada, M.; Ehara, M.; Toyota, K.; Fukuda, R.; Hasegawa, J.; Ishida, M.; Nakajima, T.; Honda, Y.; Kitao, O.; Nakai, H.; Vreven, T.; Montgomery, J. A., Jr.; Peralta, J. E.; Ogliaro, F.; Bearpark, M.; Heyd, J. J.; Brothers, E.; Kudin, K. N.; Staroverov, V. N.; Kobayashi, R.; Normand, J.; Raghavachari, K.; Rendell, A.; Burant, J. C.; Iyengar, S. S.; Tomasi, J.; Cossi, M.; Rega, N.; Millam, J. M.; Klene, M.; Knox, J. E.; Cross, J. B.; Bakken, V.; Adamo, C.; Jaramillo, J.; Gomperts, R.; Stratmann, R. E.; Yazyev, O.; Austin, A. J.; Cammi, R.; Pomelli, C.; Ochterski, J. W.; Martin, R. L.; Morokuma, K.; Zakrzewski, V. G.; Voth, G. A.; Salvador, P.; Dannenberg, J. J.; Dapprich, S.; Daniels, A. D.; Farkas, Ö.; Foresman, J. B.; Ortiz, J. V.; Cioslowski, J.; Fox, D. J. Gaussian, Inc., Wallingford CT, 2009.

10. Karlsson, C.; Jämstorp, E.; Strømme, M.; Sjödin, M., *J. Phys. Chem. C.* **2011**, 116, 3793-3801.
11. Karlsson, C.; Gogoll, A.; Strømme, M.; Sjödin, M., *J. Phys. Chem. C.* **2012**, 117, 894-901.
