# Supplementary material for: Hydroquinone–pyrrole dyads with varied linkers
Source: Beilstein J Org Chem. 2016 Jan 18;12:89–96. doi: 10.3762/bjoc.12.10 (PMC4734300; doi:10.3762/bjoc.12.10)
Supplement: File 2 — Optimization of experimental conditions, computated electron distributions, UV–vis, IR, and NMR spectral data. [file Beilstein_J_Org_Chem-12-89-s002.pdf]

Supporting Information

for

Hydroquinone–pyrrole dyads with varied linkers

Hao Huang[1], Christoffer Karlsson[1], Maria Strømme[1], Martin Sjödin[1] and Adolf Gogoll*,[2]

Address: [1] Nanotechnology and Functional Materials, Department of Engineering Sciences, The Ångström Laboratory, Uppsala University, Box 534, SE-751 21 Uppsala, Sweden and [2] Department of Chemistry - BMC, Biomedical Centre, Uppsala University, Box 576, SE-751 23 Uppsala, Sweden

Email: Adolf Gogoll - adolf.gogoll@kemi.uu.se

*Corresponding author

Optimization of experimental conditions, computated electron distributions, UV–vis, IR, and NMR spectral data

Contents

Optimization of experimental conditions S2

Table S1: Experimental UV–vis absorptions and calculated UV–vis absorptions for **3a**–**d** and **1** S4

Figure S1–S6: Energy diagrams for **3a**–**d** and **1** S5–S10

Figure S7–S16: IR spectra of **3a**–**d** and **4a**–**d** S11–S15

Figure S14–S40: NMR and mass spectra of **3a**–**3d** and **4a**–**4d** S16–S40

Calculated NMR chemical shifts S41

Optimization attempts for Heck reaction: Synthesis of 4c.

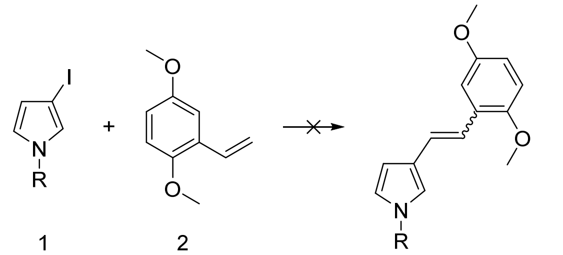

Trial	R	Solvent	Temperature	Catalyst	Base	additives	time
1	TIPS	DMF	150	$Pd(OAc)_2$	K_2CO_3	TBAB	overnight
2	TIPS	DMF	80	$Pd(OAc)_2$	KOAc	TBAB	overnight
3	TIPS	DMF	150	$PdCl_2$	KOAc	MeOH	overnight
4	TIPS	MeCN	80	$Pd(OAc)_2$	Et_3N	PPh_3	overnight
5	TIPS	Toluene	150	$Pd(PPh_3)_4$	Et_3N		20 mins
6	TIPS	Dioxane	150	$Pd_2(dba)_3$	$CsCO_3$	$P(^tBu)_3$	2 h
7	Tosyl	DMF	120	$Pd(PPh_3)_2Cl_2$	Et_3N		1 h
8	Boc	DMF	120	$Pd(PPh_3)_2Cl_2$	Et_3N		1 h

Procedure:

To a solution of **1** (50 mg, 0.14 mmol), **2** (1.2 eq.), base (2 eq.) and additives (for TBAB 1.5 eq. respectly, for PPh3 and $P(^tBu)_{3,}$ 0.1 eq respectly, MeOH was added as co-solvent)in the dry solvent in a heavy-wall Smith process vial, palladium catalyst (0.05 eq.)was added, the vial was sealed and then heat it up in oil bath at the indicated temperature (according to the description of reaction vial provider, this vial sealed can withstand pressures beyond 30 bar in a wide range of conditions). No product was formed in any of the trials.

Optimization attempts for hydrogenation of 4d.

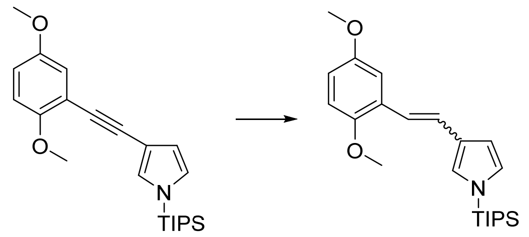

**Trial**	**Condition**	**Yield**	**E:Z** ratio
1[a]	Lindlar catalyst, quinoline, benzene, RT, H_2 (1 atm), overnight	No reaction	n. a.
2[b]	$HSiEt_3$, $Pd(PPh_3)_2Cl_2$, dppf, Cu_2SO_4, Toluene/H_2O, 100 °C, H_2 (1 atm), overnight	34 %	E:Z =3:1
3[c]	$Pd(OAc)_2$, KOH, DMF, 145 °C, H_2 (1 atm), 3 h	25 %[d]	Z only

[a] To a benzene solution (2 ml) of Lindlar catalyst (33 mg, 5 % Pd/C loading) suspended in a 10 ml round-bottom flask, fresh distilled quinoline (2 ul) was added. The resulting solution was degassed by switching between vacuum and Ar gas for 5 times, and then connected with a hydrogen reservoir. Bubbling H_2 gas for 5 mins and then the starting material (60 mg, 0.16 mmol) in 2 ml benzene was added. The reaction mixture was stirred overnight.

[b] Luo, F.; Pan, C.; Wang, W.; Ye, Z.; Cheng, J., *Tetrahedron* **2010,** *66* (6), 1399-1403.

[c] J. Li, R. Hua, T. Liu, *J. Org. Chem.* **2010**, *75*, 2966-2970.

[d] Deprotection occurred during reaction, the yield was calculated referring to deprotected product.

Table S 1. Wavelength (λ , nm) and extinction coefficient (ε , $cm^{-1}M^{-1}$) in UV-vis spectra of compounds **3a** – **3d** and **1**, measured for MeCN solutions, and calculated wavelengths (λ , nm) with corresponding oscillator strength(*f*).

Compound	Experimental		Calculation	
	λ	ε	λ	*f*
**3a**	221	16830	229	0.07
	290	5060	272	0.114
**3b**	224	11080	229	0.07
	290	3580	272	0.11
trans-**3c**	222	7030	230	0.11
	298	4510	295	0.2
	336	4450	337	0.82
cis-**3c**	230	14860	240	0.09
	297	8150	288	0.11
	325	7220	314	0.29
**3d**	217	37560	228	0.14
	252	12940	248	0.08
	273	14540	263	0.1
	288	20640	287	0.19
	322	18260	328	0.66
**1**	214	27090	237	0.09
	265	9590	251	0.08
	270	8970	264	0.1
	308	7840	297	0.3

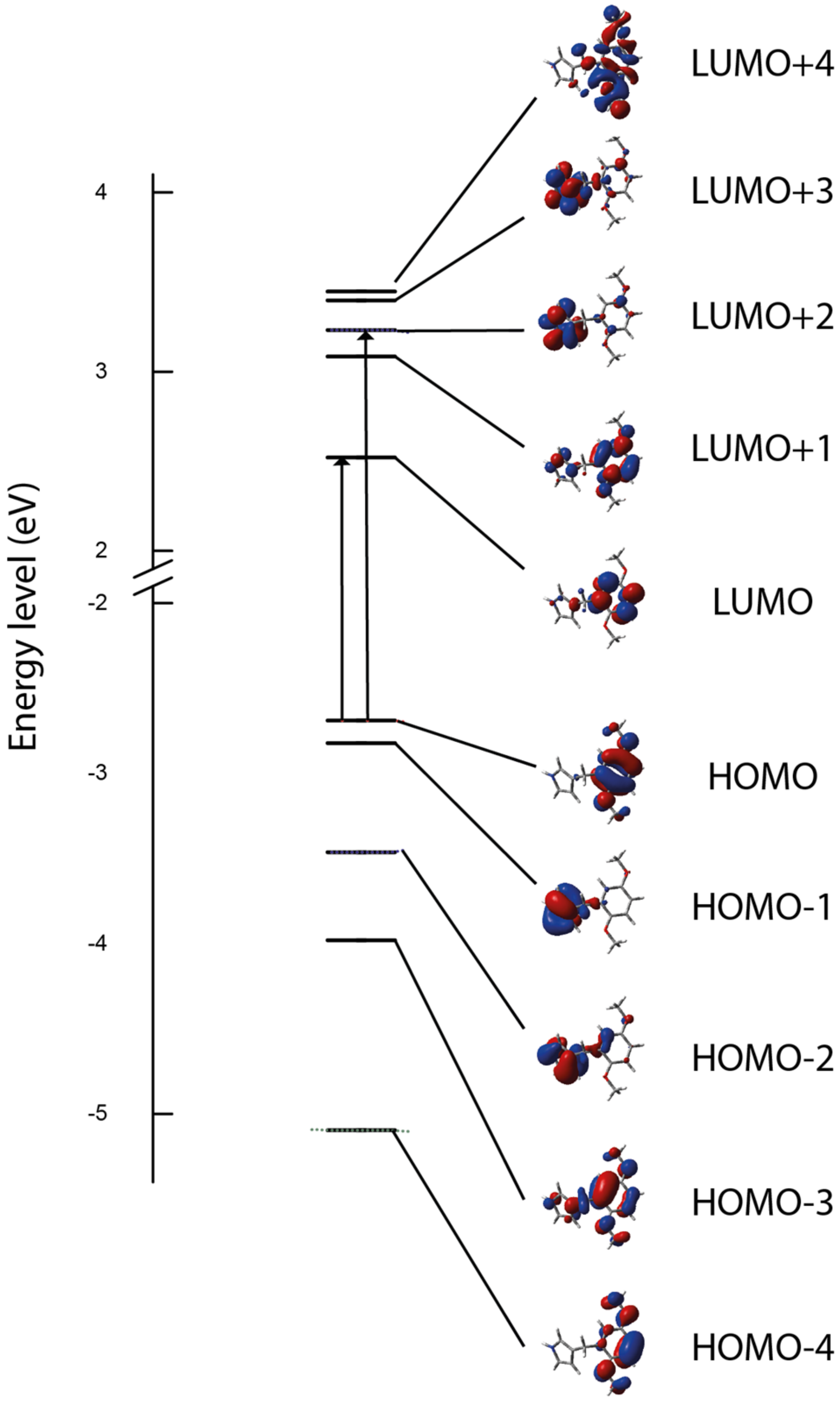

Figure S 1. Energy diagram of 3a with MOs, transitions indicated by calculations marked by arrows.

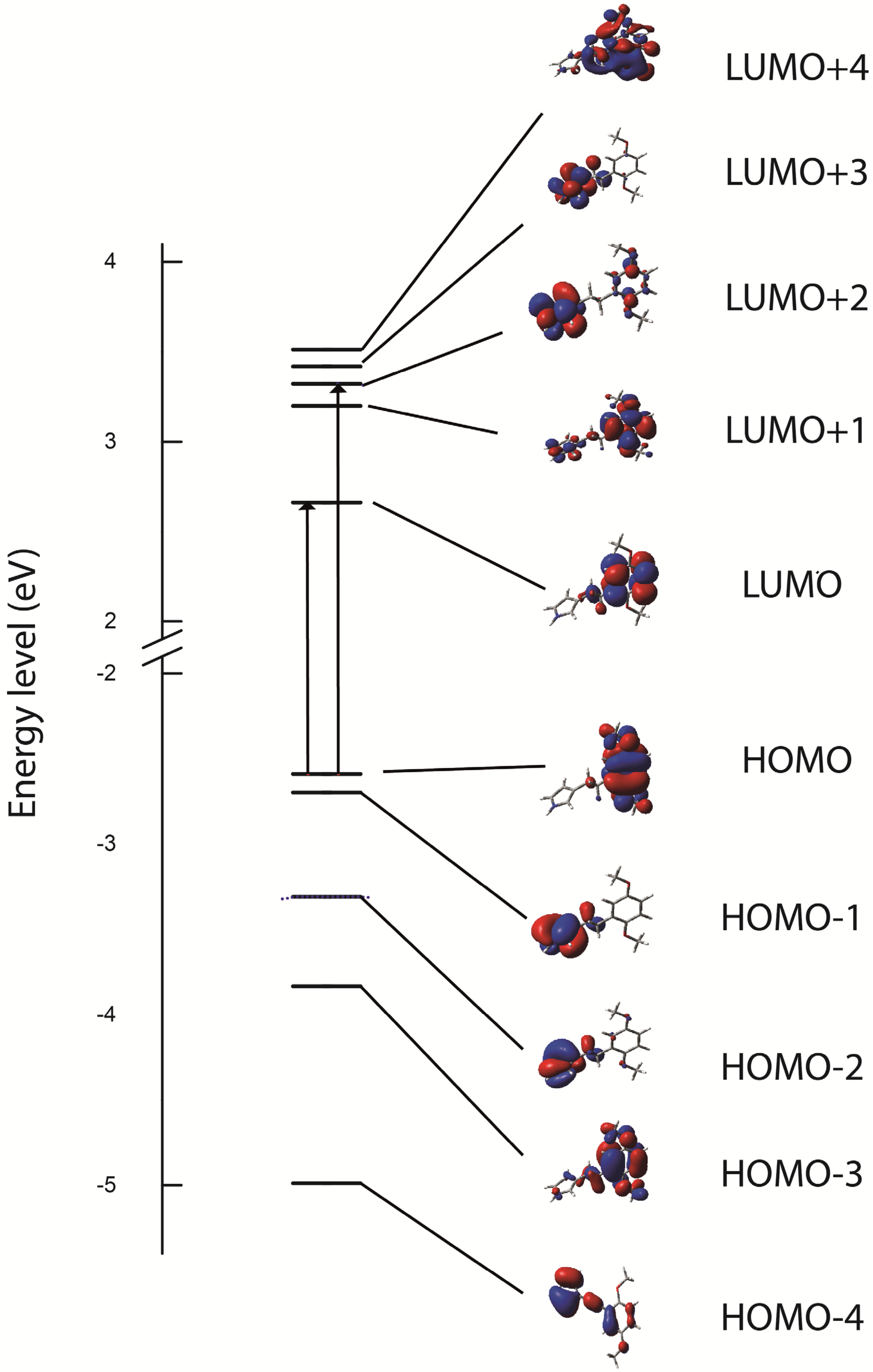

Figure S 2. Energy diagram of **3b** with MOs**,** transitions indicated by calculations marked by arrows.

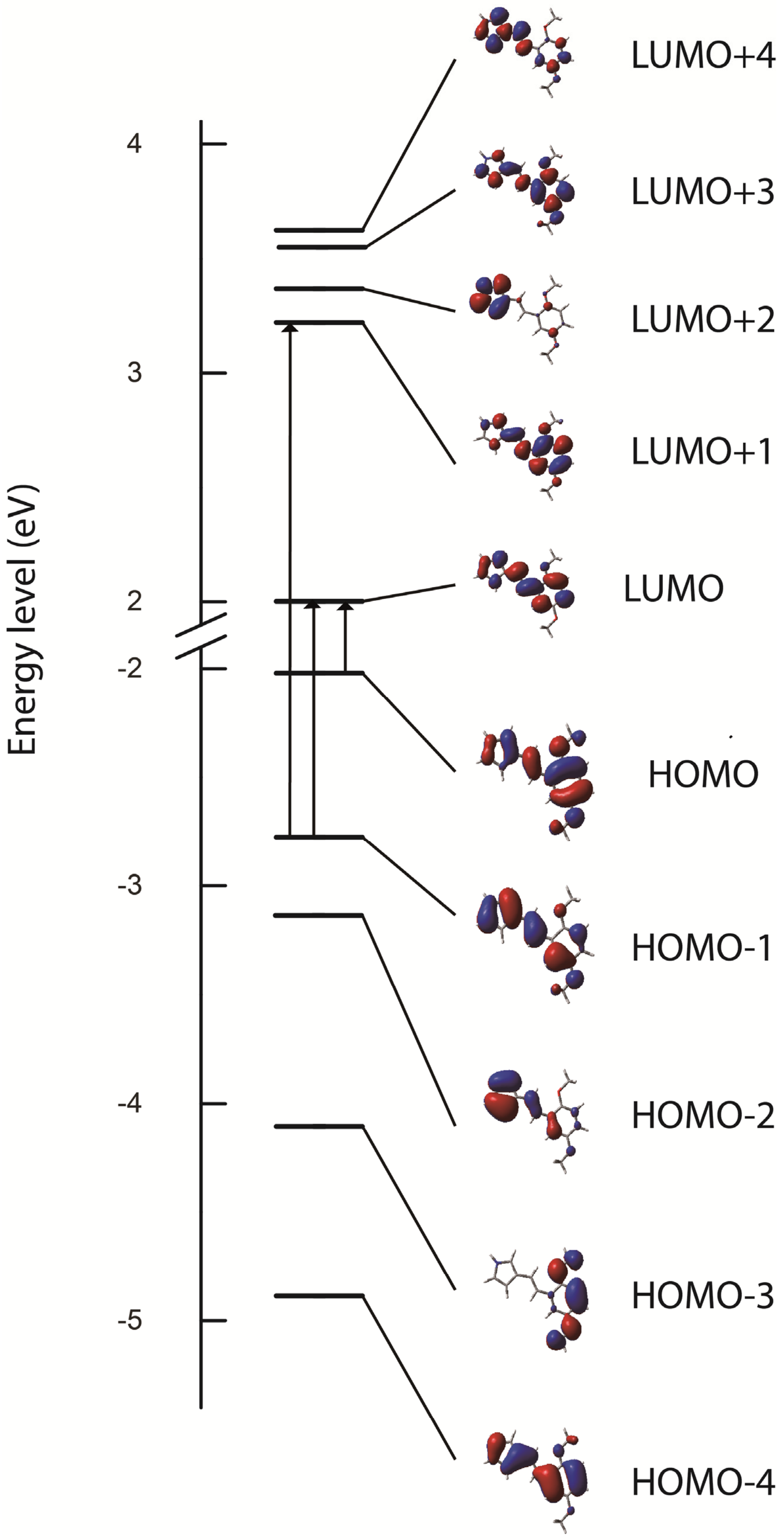

Figure S 3. Energy diagram of *trans*-**3c** with MOs**,** transitions indicated by calculations marked by arrows.

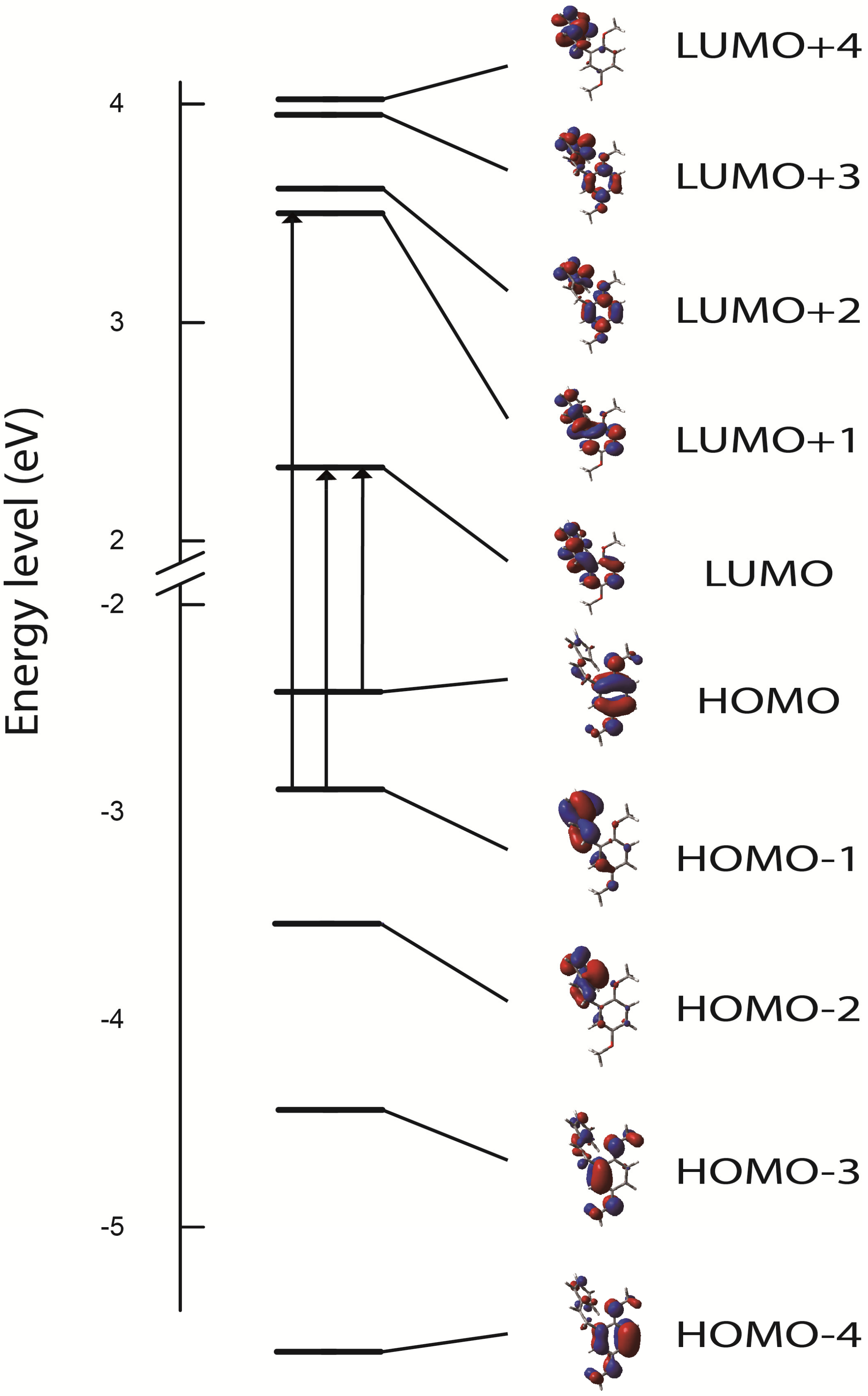

Figure S 4. Energy diagram of *cis*-**3c** with MOs**,** transitions indicated by calculations marked by arrows.

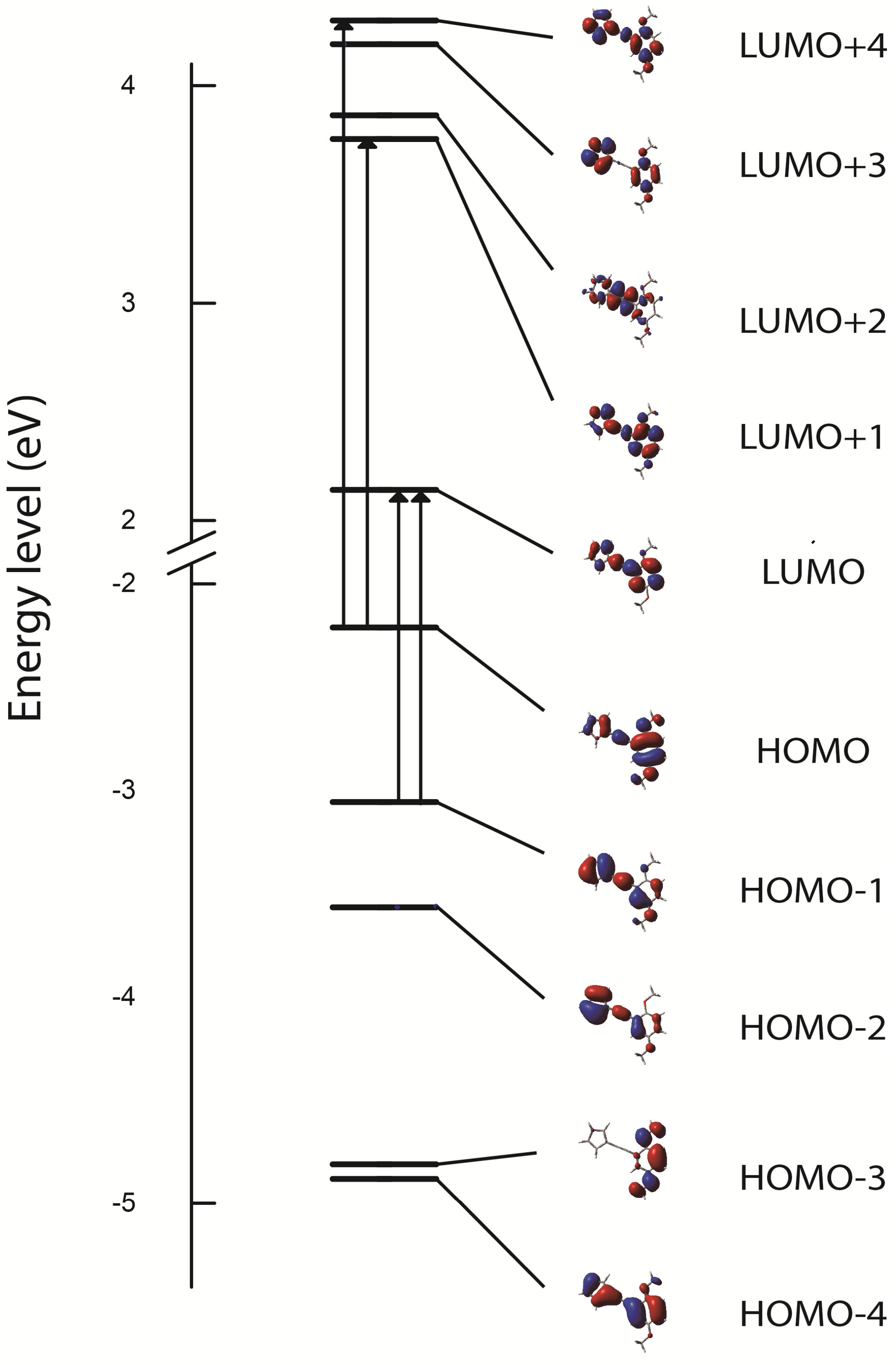

Figure S 5. Energy diagram of **3d** with MOs**,** transitions indicated by calculations marked by arrows.

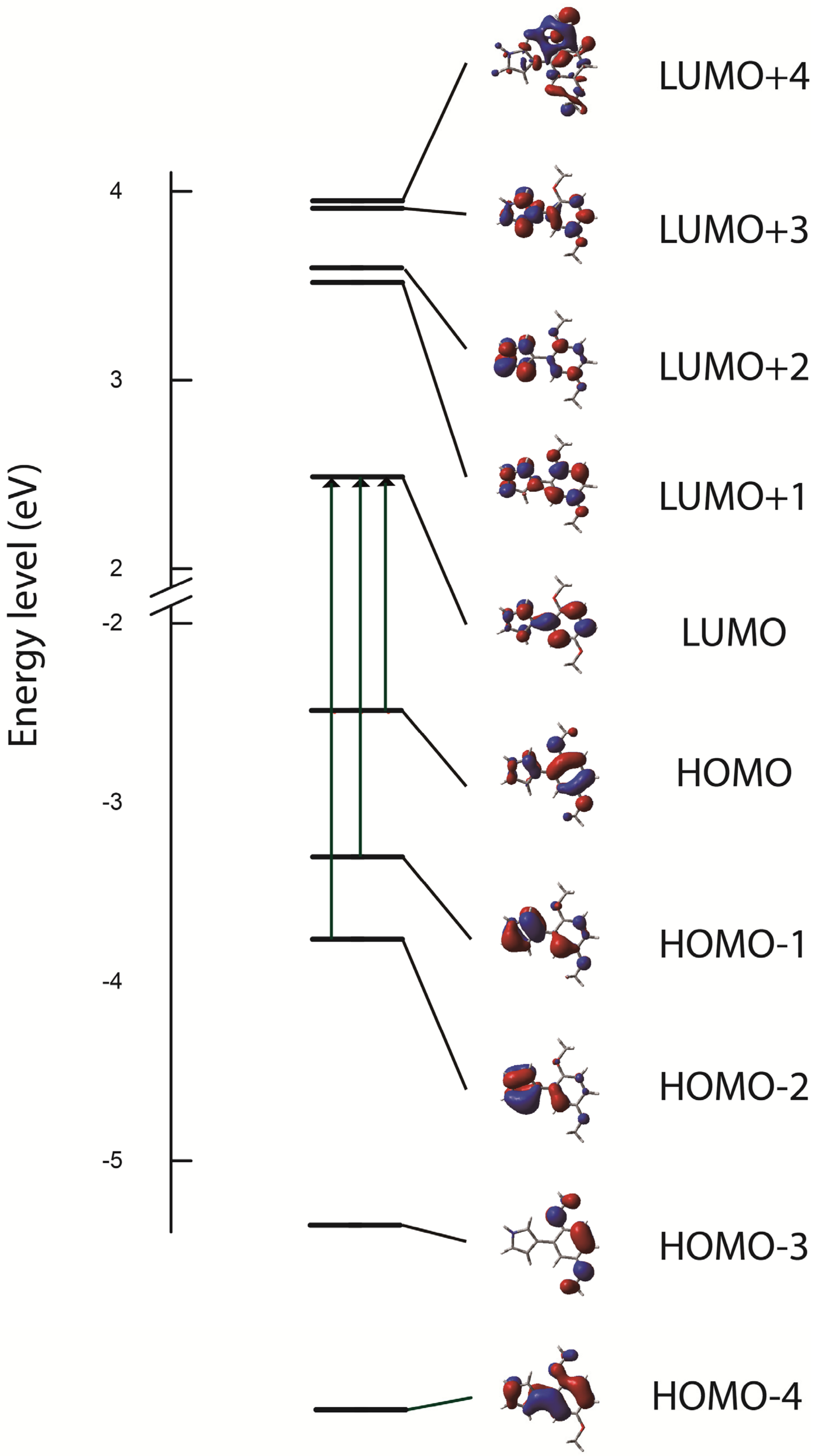

Figure S 6. Energy diagram of **1** with MOs**,** transitions indicated by calculations marked by arrows.

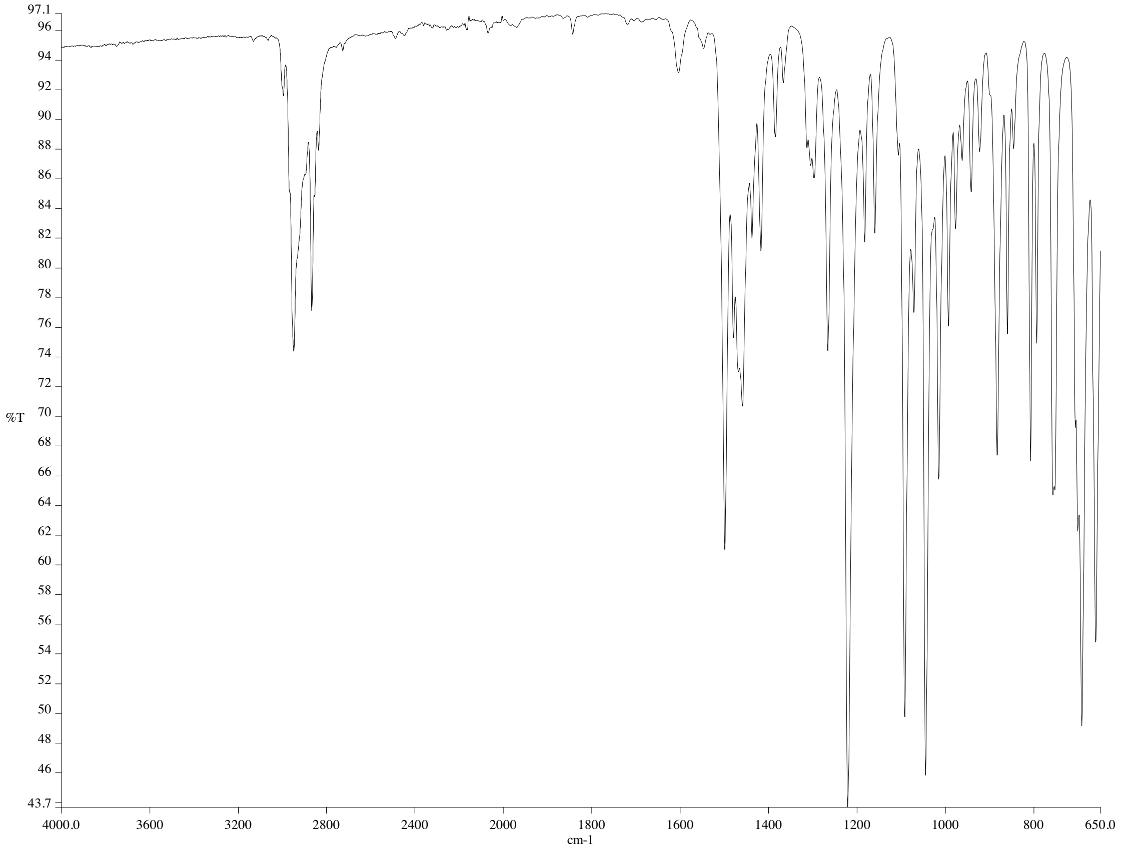

Figure S 7. IR spectrum of **4a**.

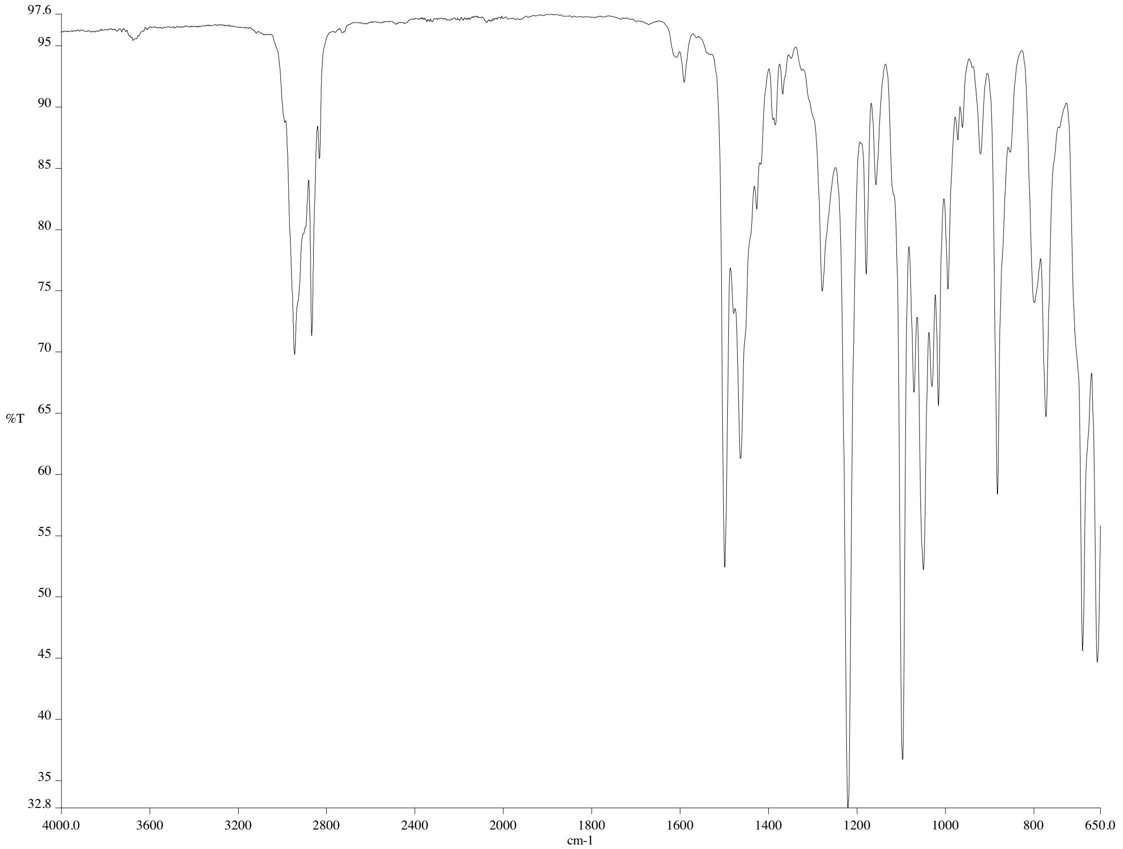

Figure S 8. IR spectrum of **4b**.

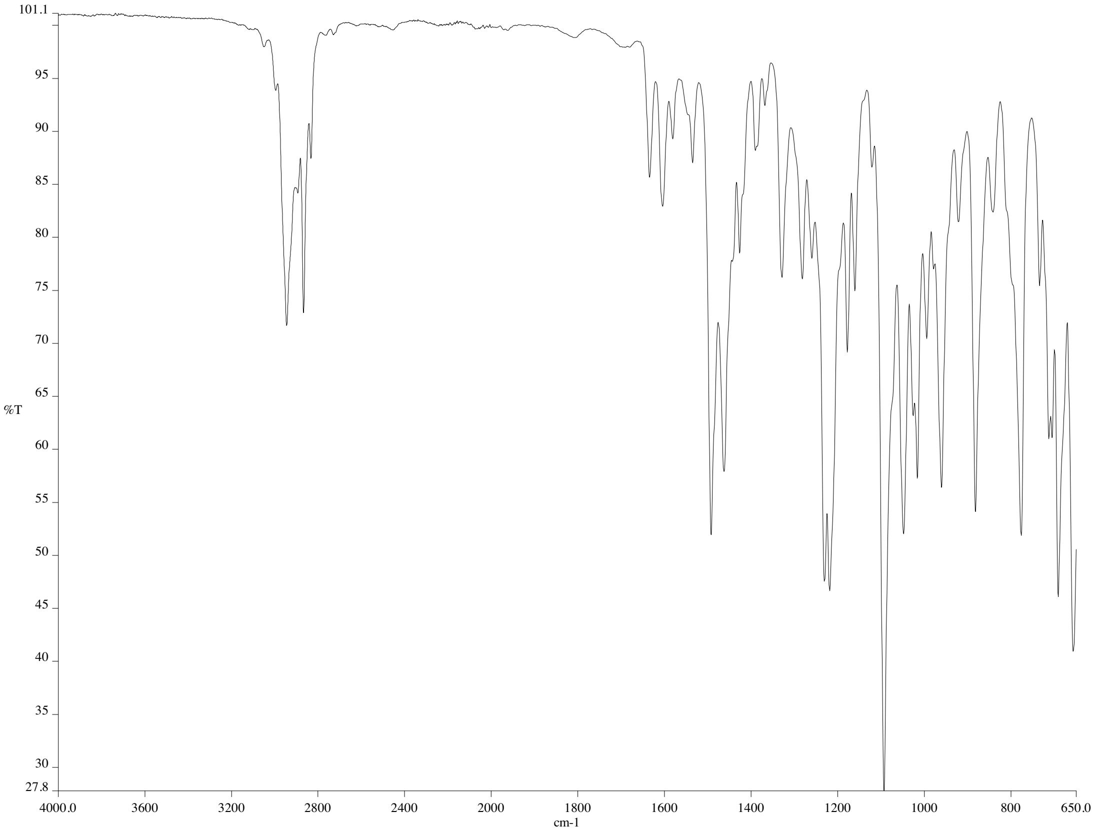

Figure S 9. IR spectrum of *trans*-**4c.**

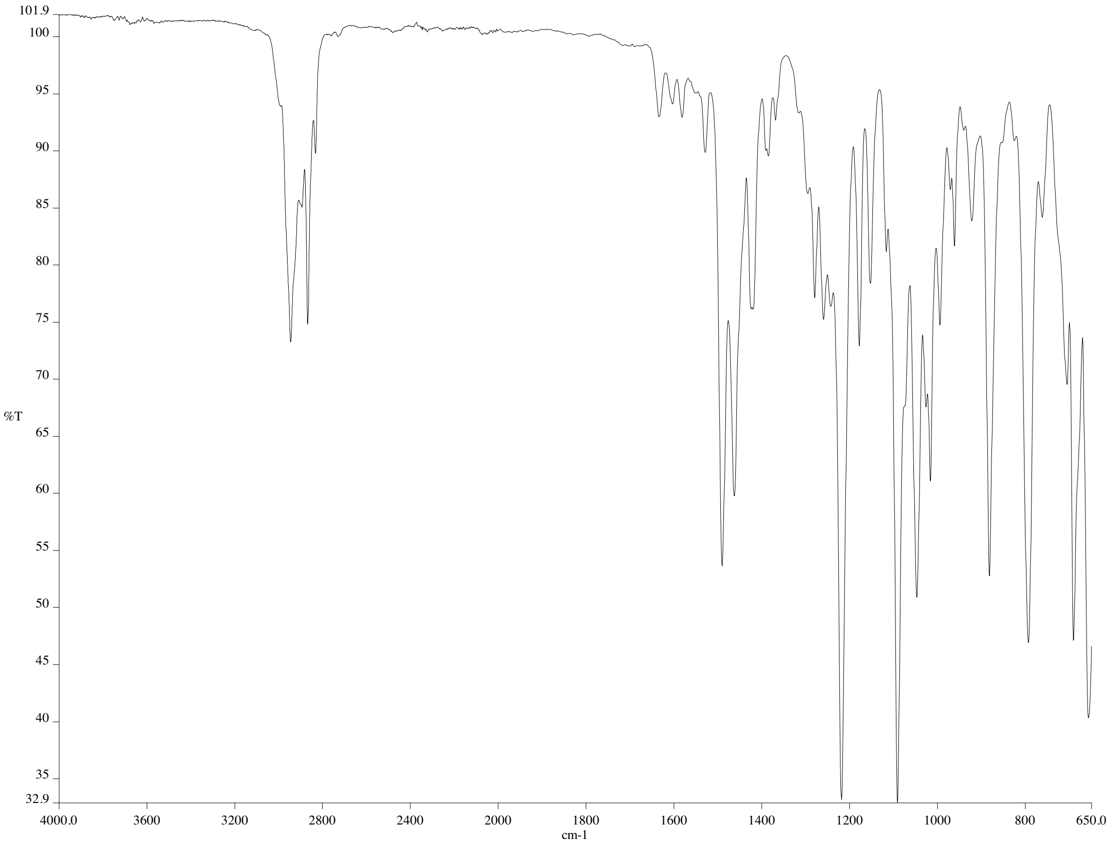

Figure S 10. IR spectrum of *cis*-**4c.**

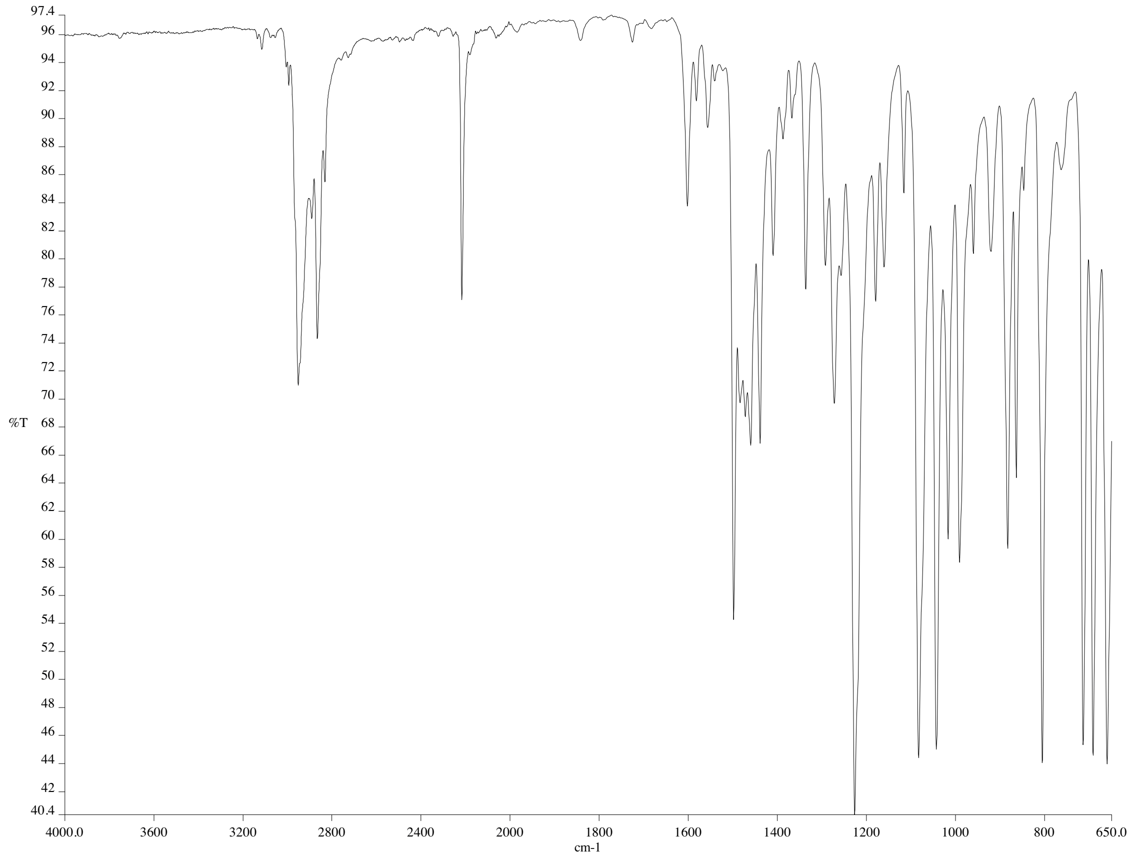

Figure S 11. IR spectrum of **4d.**

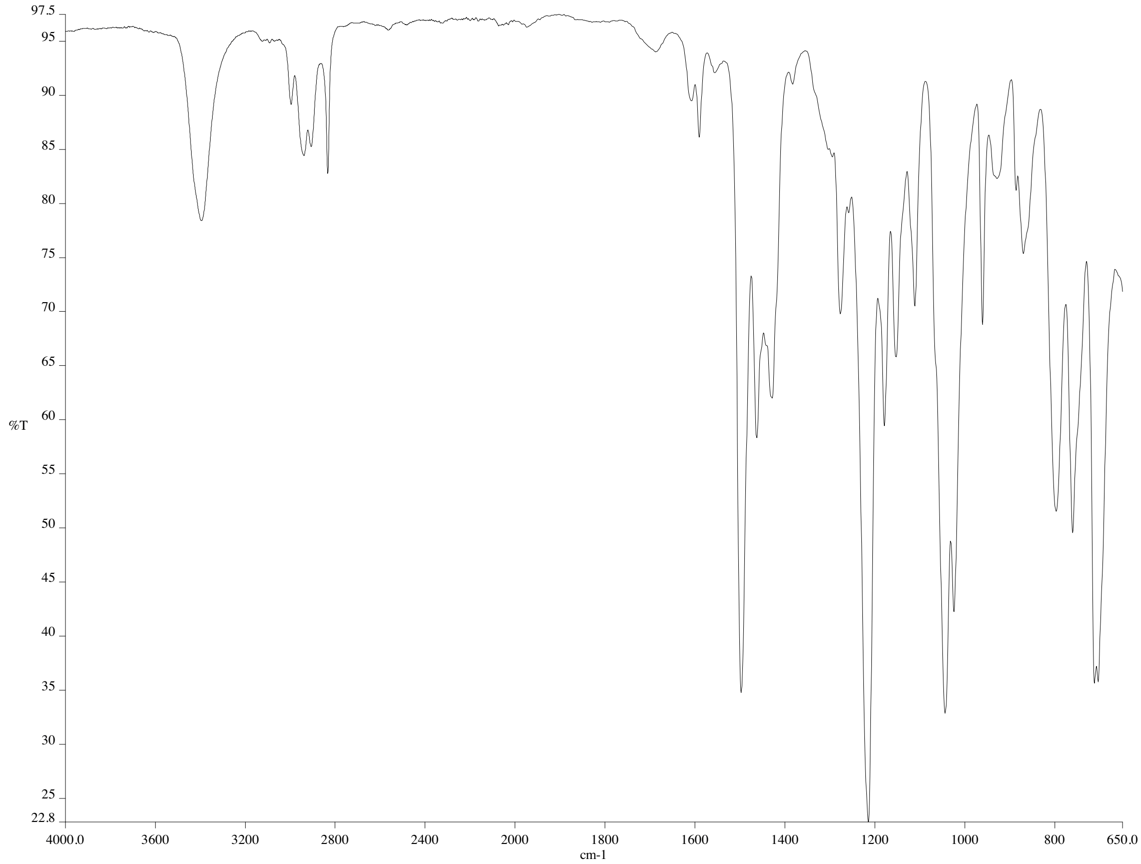

Figure S 12. IR spectrum of **3a.**

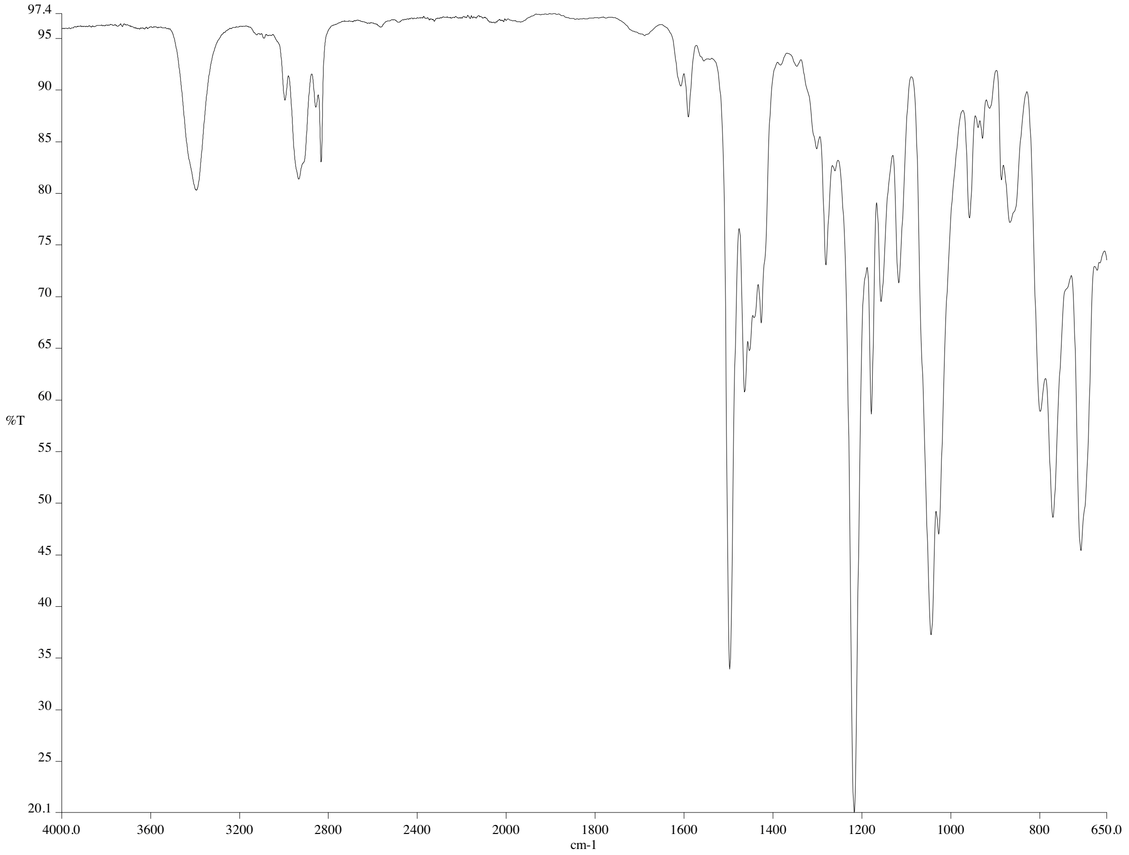

Figure S 13. IR spectrum of **3b.**

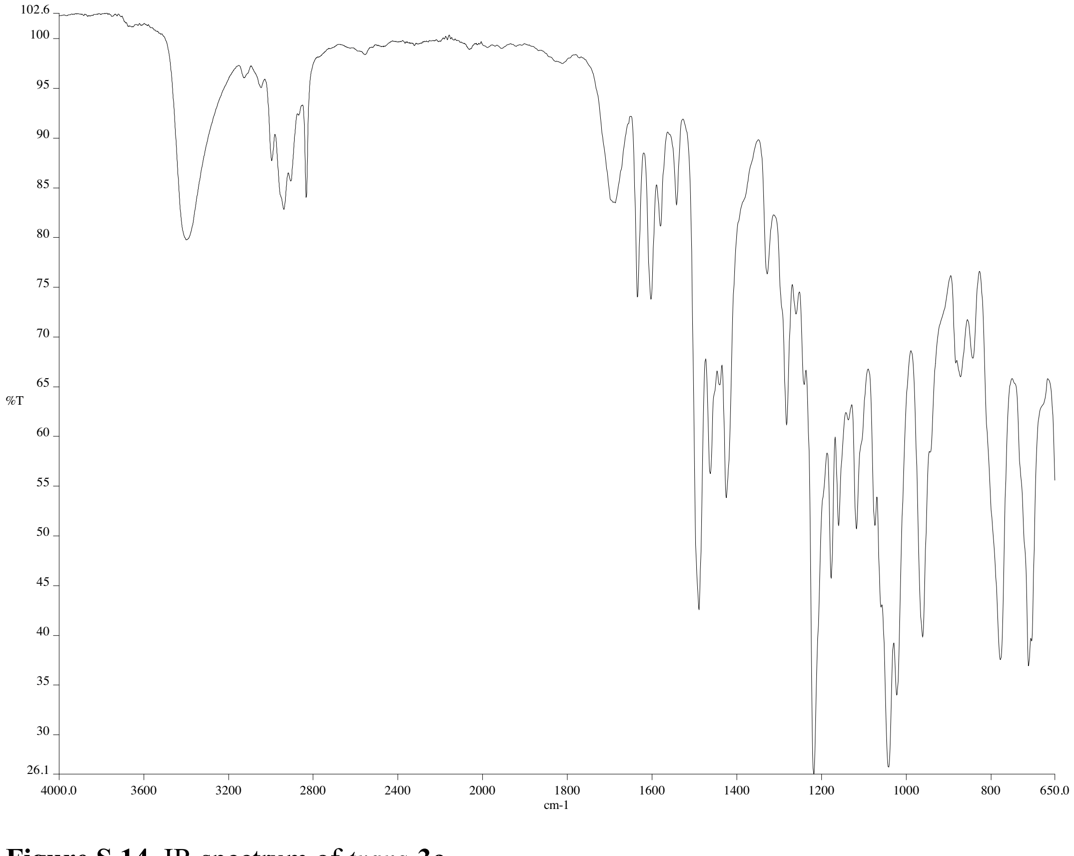

Figure S 14. IR spectrum of *trans*-**3c.**

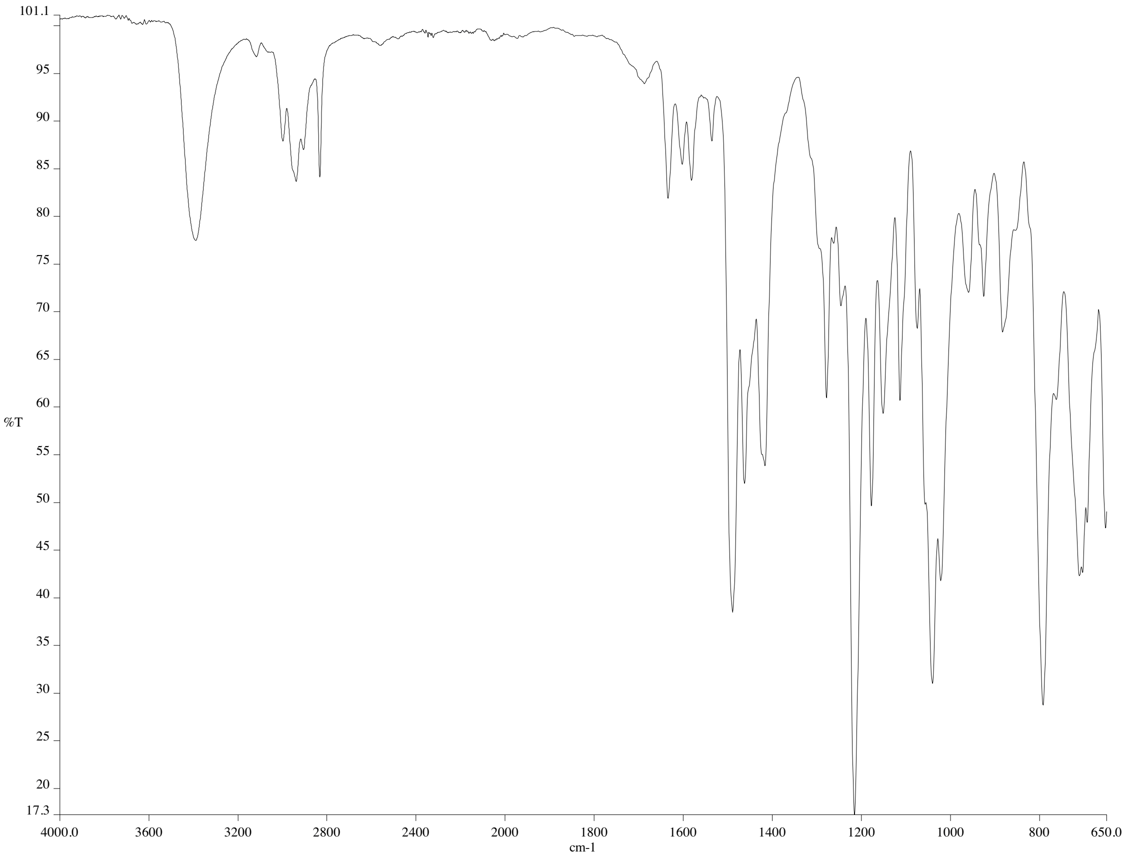

Figure S 15. IR spectrum of *cis*-**3c.**

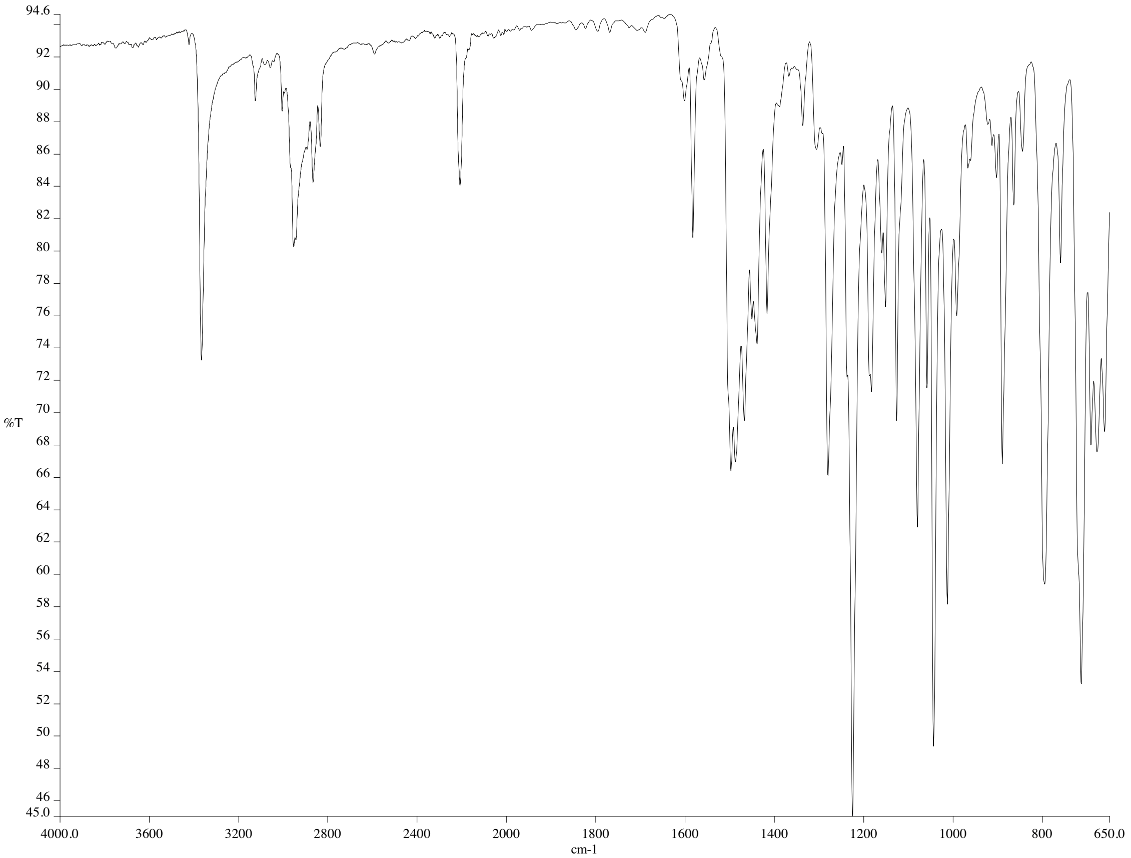

Figure S 16. IR spectrum of **3d.**

NMR spectra

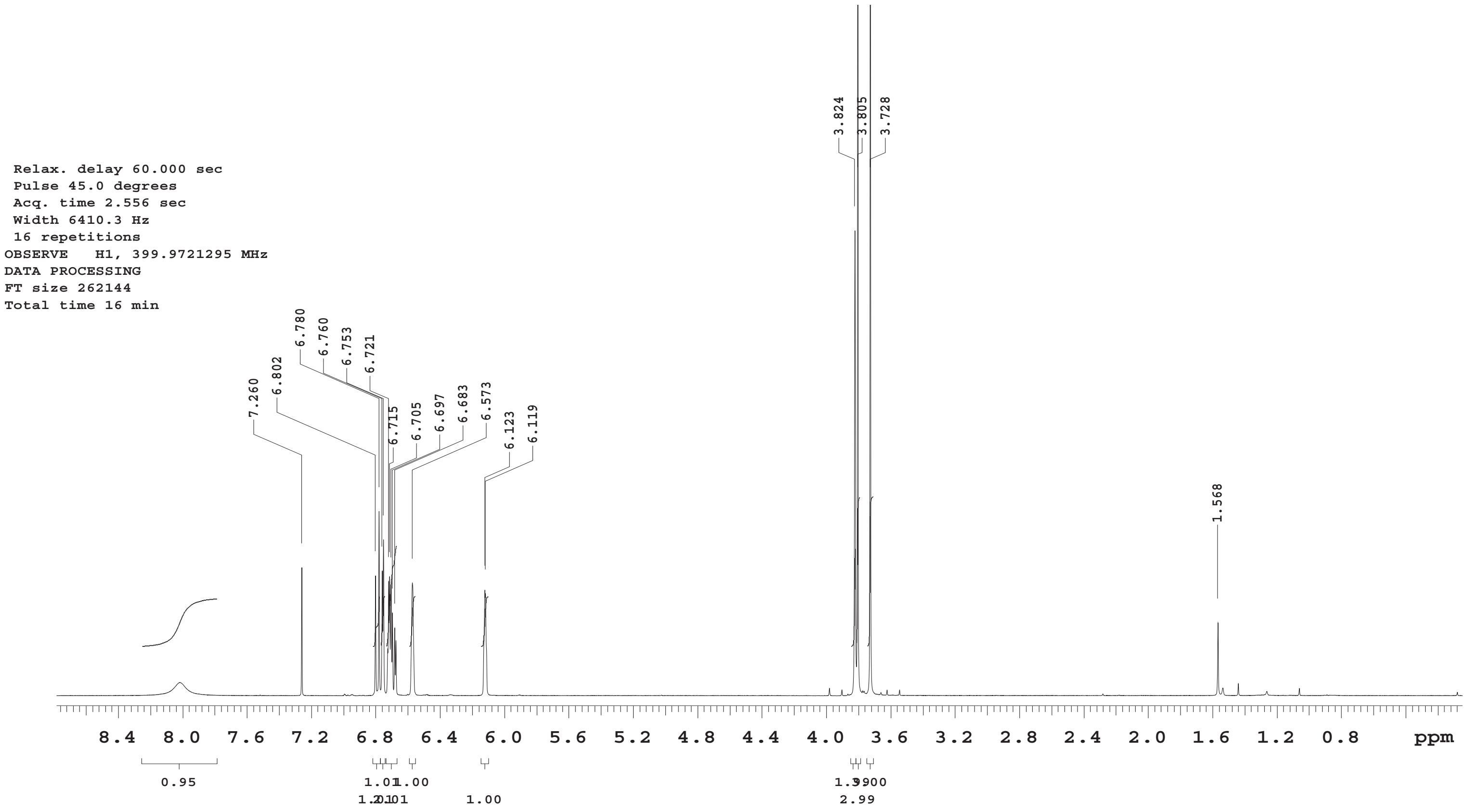

Figure S 17. ^{1}H NMR spectrum (400 MHz, $CDCl_3$ solution) of **3a**.

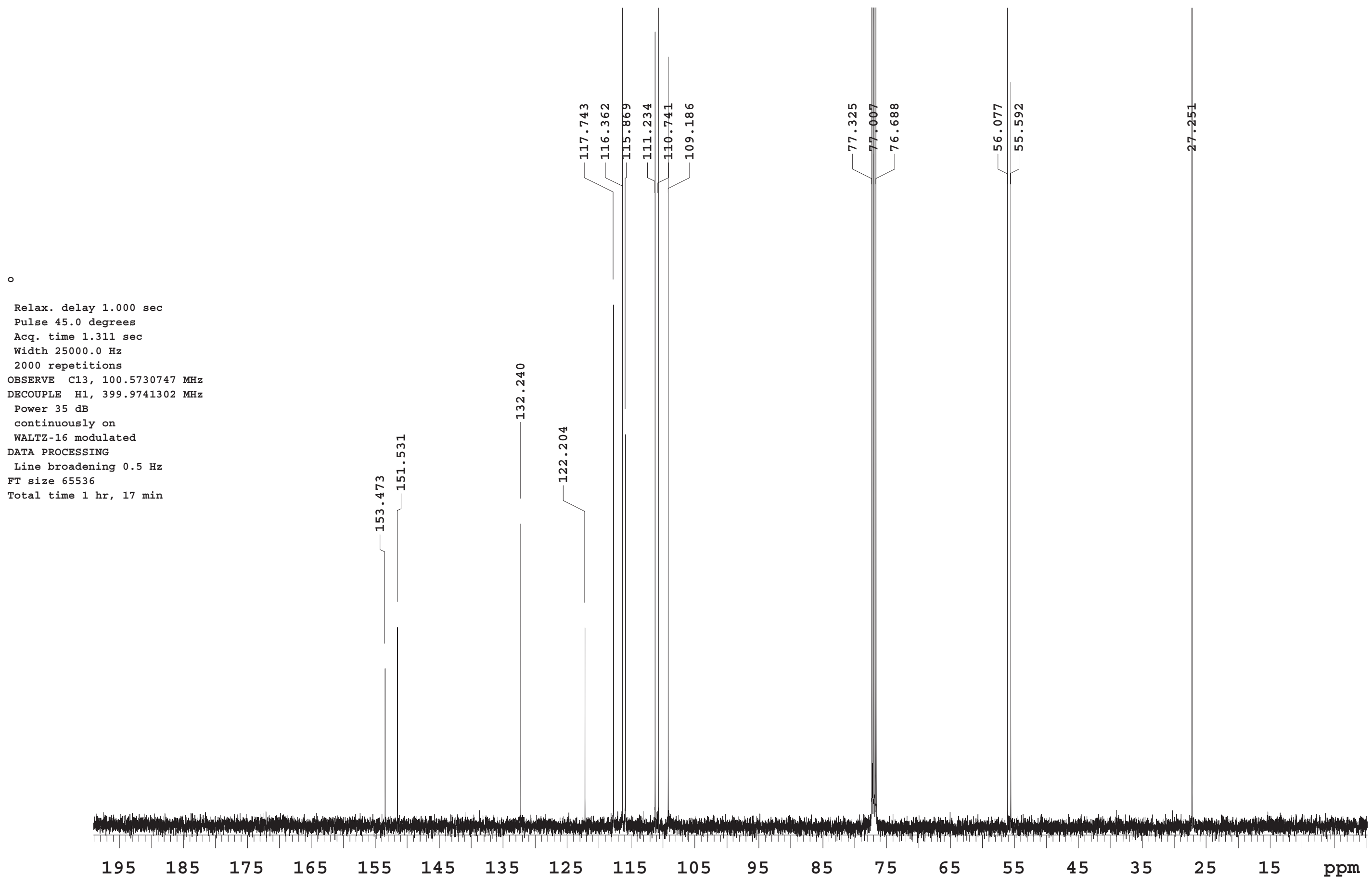

Figure S 18. ^{13}C NMR spectrum (100.6 MHz, $CDCl_3$ solution) of **3a**.

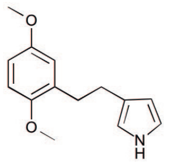

3b

Temp. 25.0 C / 298.1 K
Sample #1, Operator: huanghao

Relax. delay 25.000 sec
Pulse 45.0 degrees
Acq. time 2.556 sec
Width 6410.3 Hz
16 repetitions
OBSERVE H1, 399.9721291 MHz
DATA PROCESSING
FT size 32768
Total time 7 min 21 sec

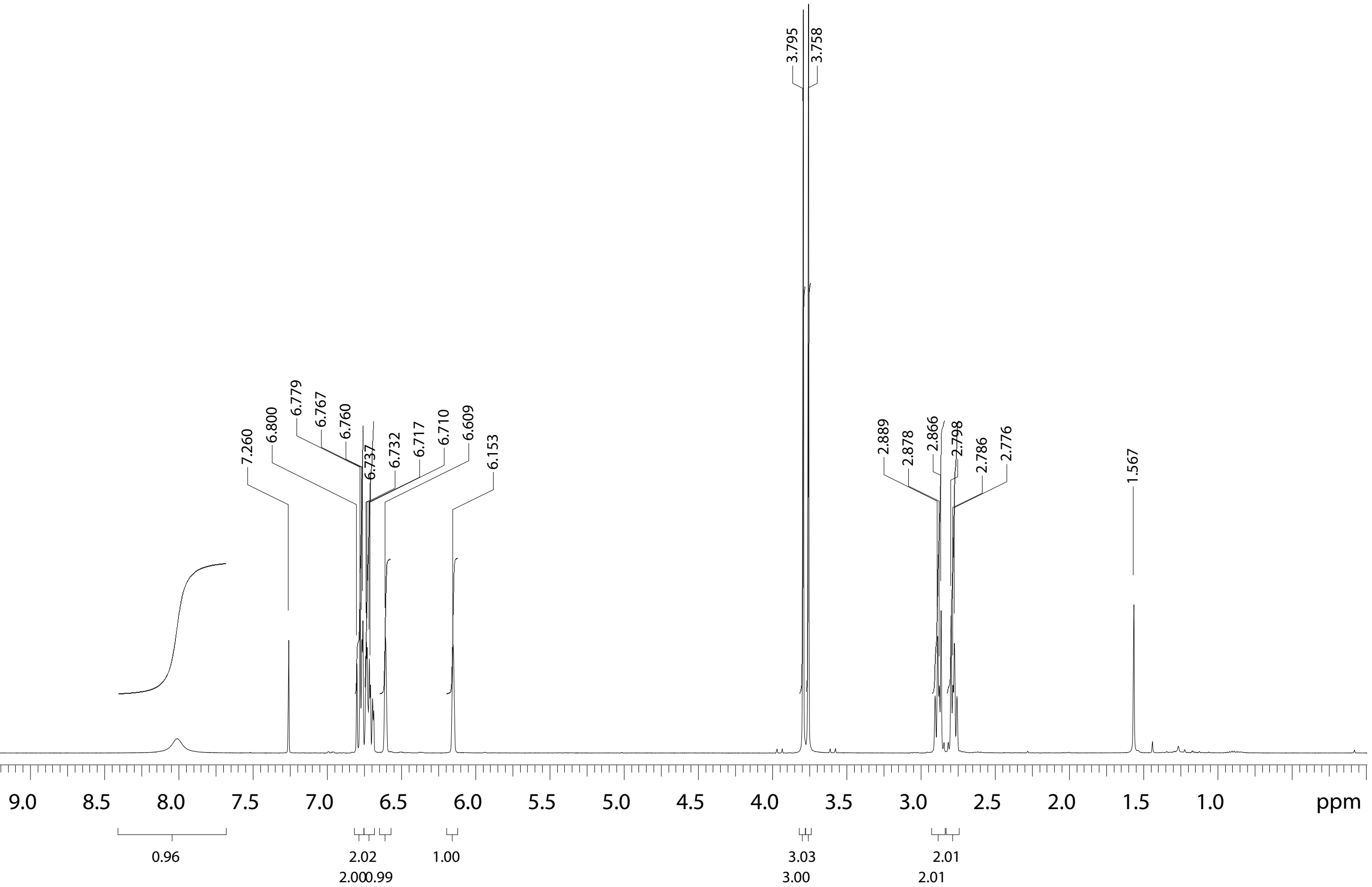

Figure S 19. ^{1}H NMR spectrum (400 MHz, $CDCl_3$ solution) of **3b**.

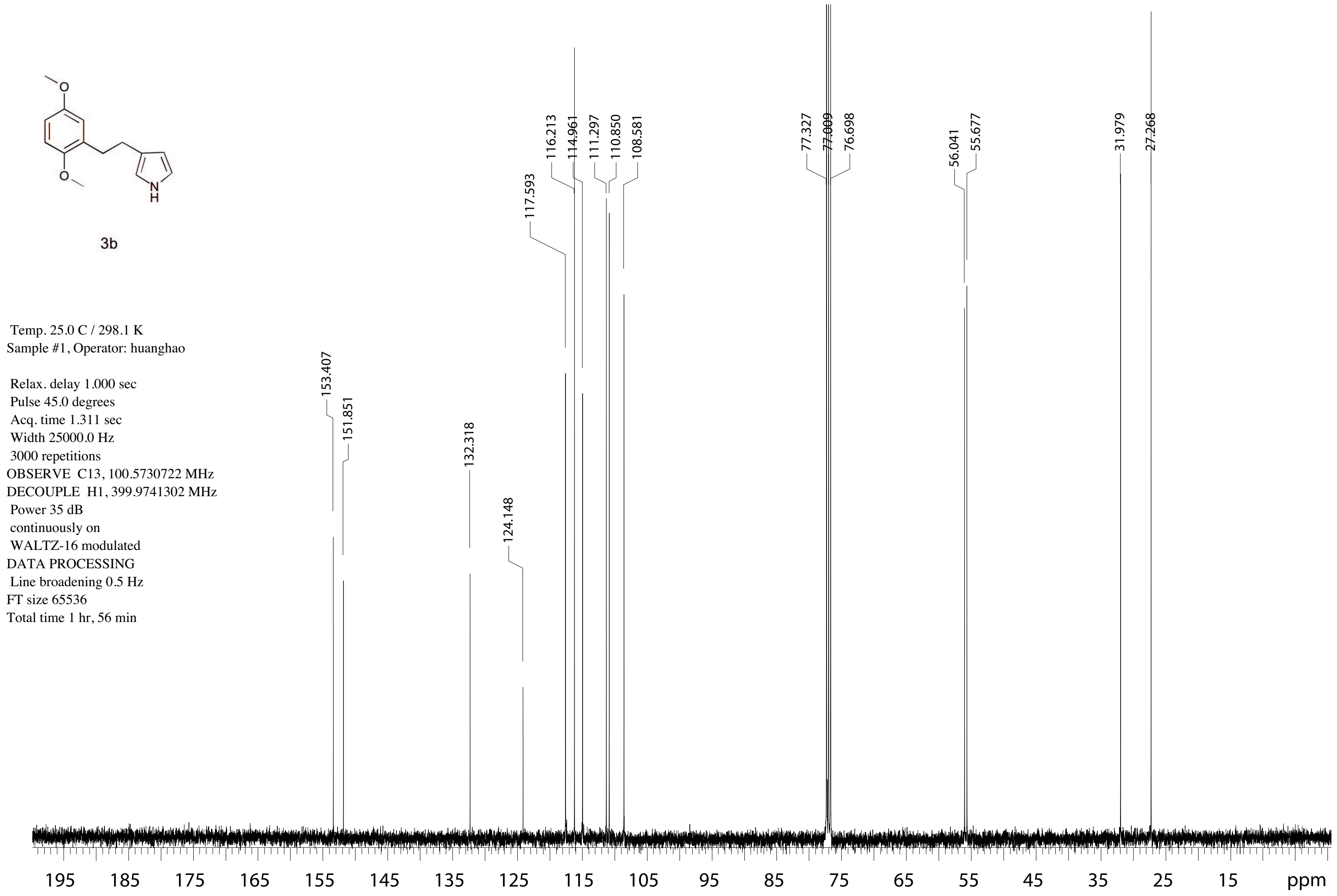

Figure S 20. ^{13}C NMR spectrum (100.6 MHz, $CDCl_3$ solution) of **3b**.

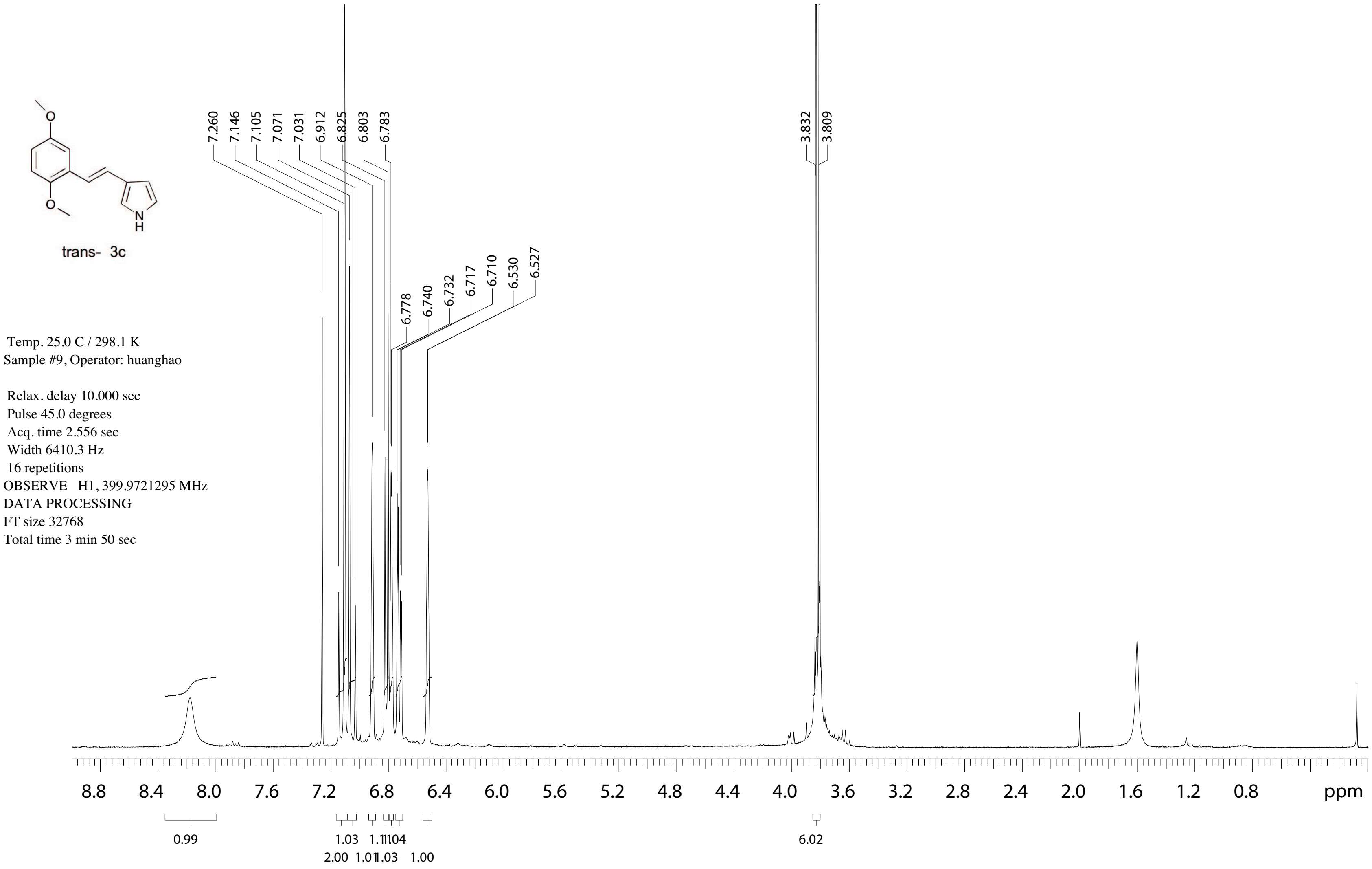

Figure S 21. ^{1}H NMR spectrum (400 MHz, $CDCl_3$ solution) of *trans*-**3c**.

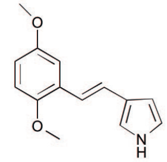

trans- 3c

Temp. 25.0 C / 298.1 K
Sample #9, Operator: huanghao

Relax. delay 1.000 sec
Pulse 45.0 degrees
Acq. time 1.311 sec
Width 25000.0 Hz
3000 repetitions
OBSERVE C13, 100.5730722 MHz
DECOUPLE H1, 399.9741302 MHz
Power 35 dB
continuously on
WALTZ-16 modulated
DATA PROCESSING
Line broadening 0.5 Hz
FT size 65536
Total time 1 hr, 55 min

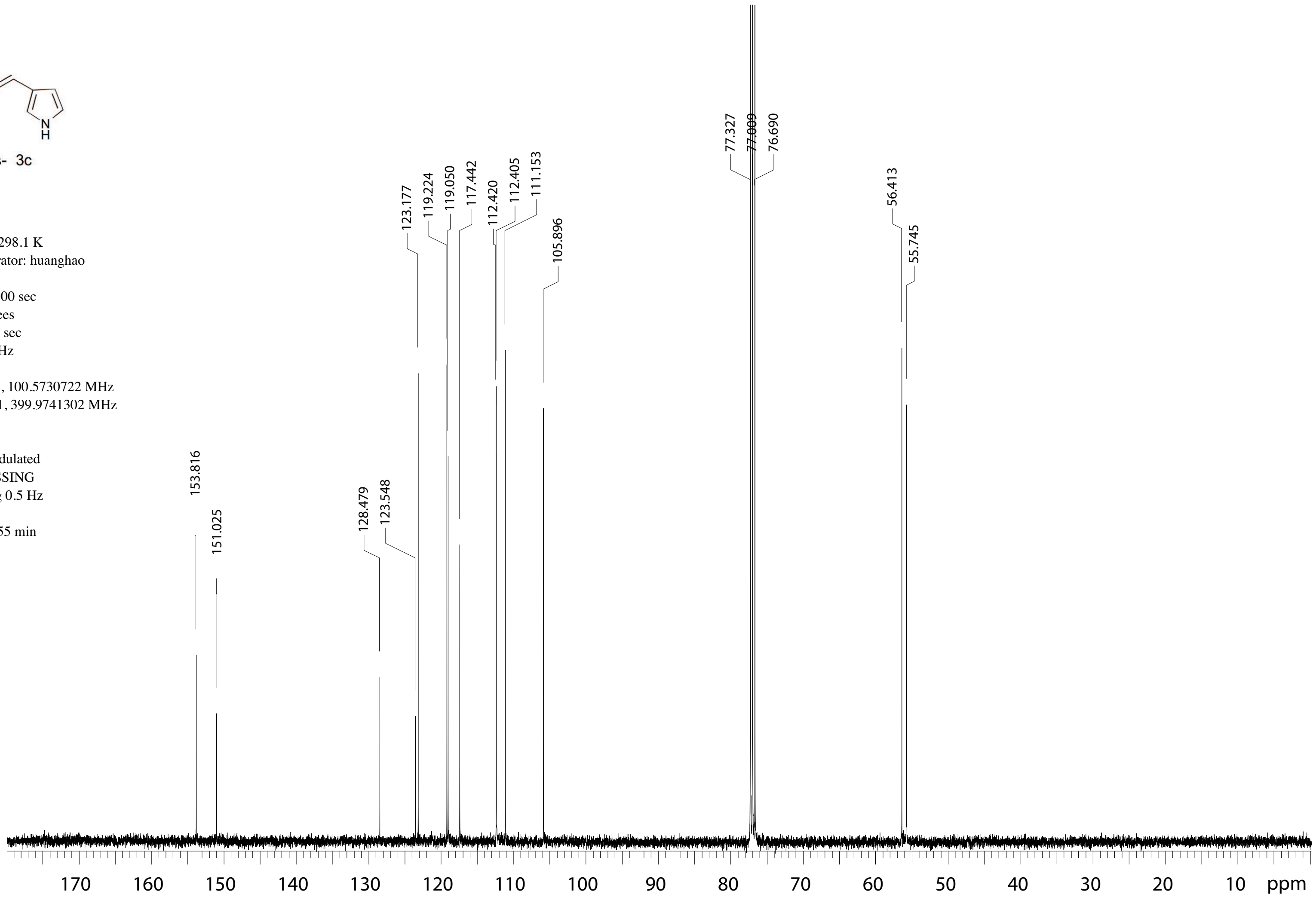

Figure S 22. ^{13}C NMR spectrum (100.6 MHz, $CDCl_3$ solution) of *trans*-**3c**.

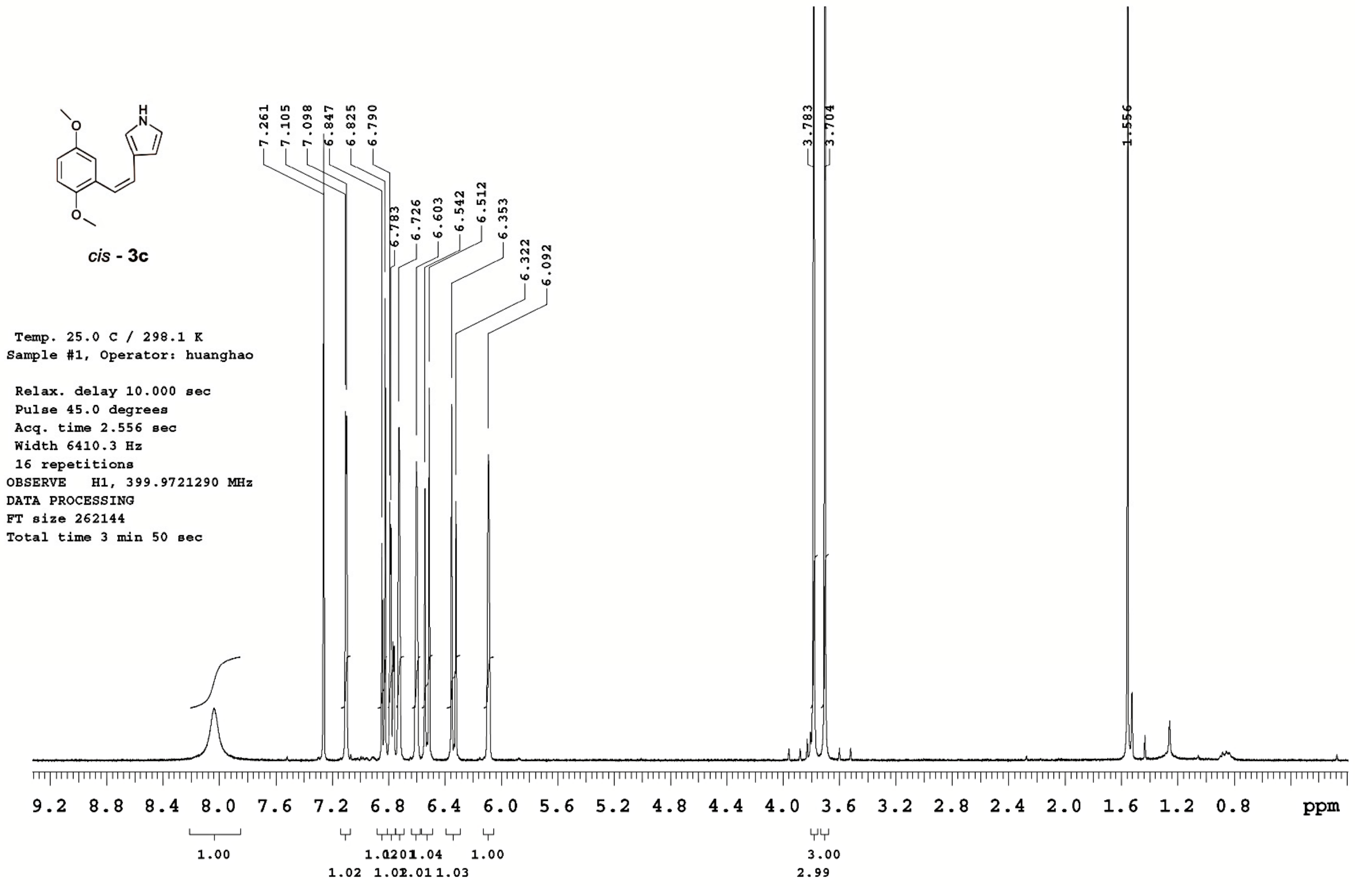

Figure S 23. ^{1}H NMR spectrum (400 MHz, $CDCl_3$ solution) of *cis*-**3c**.

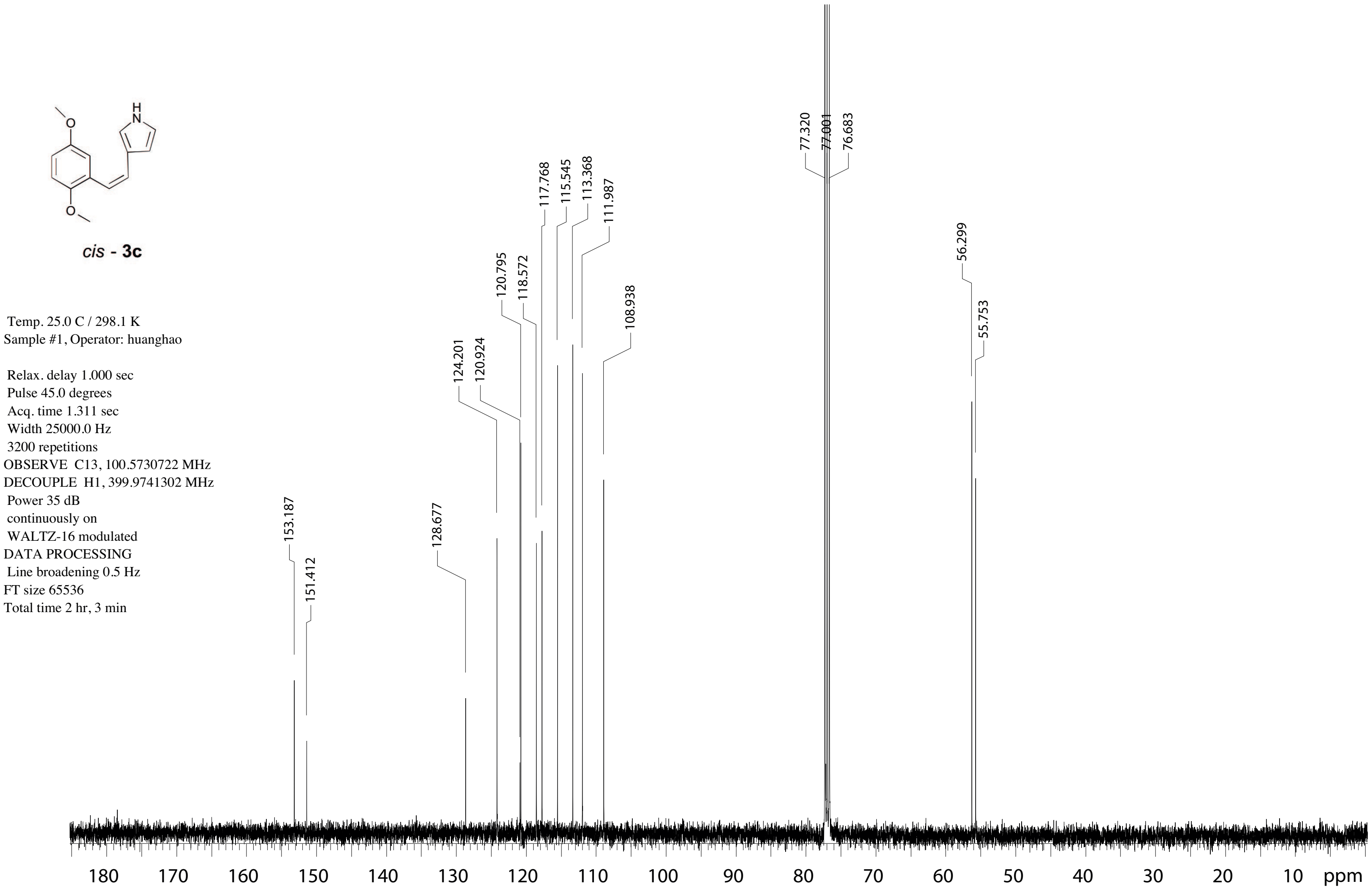

Figure S 24. ^{13}C NMR spectrum (100.6 MHz, $CDCl_3$ solution) of *cis*-**3c**.

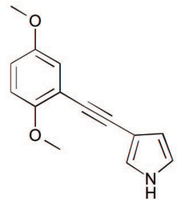

3d

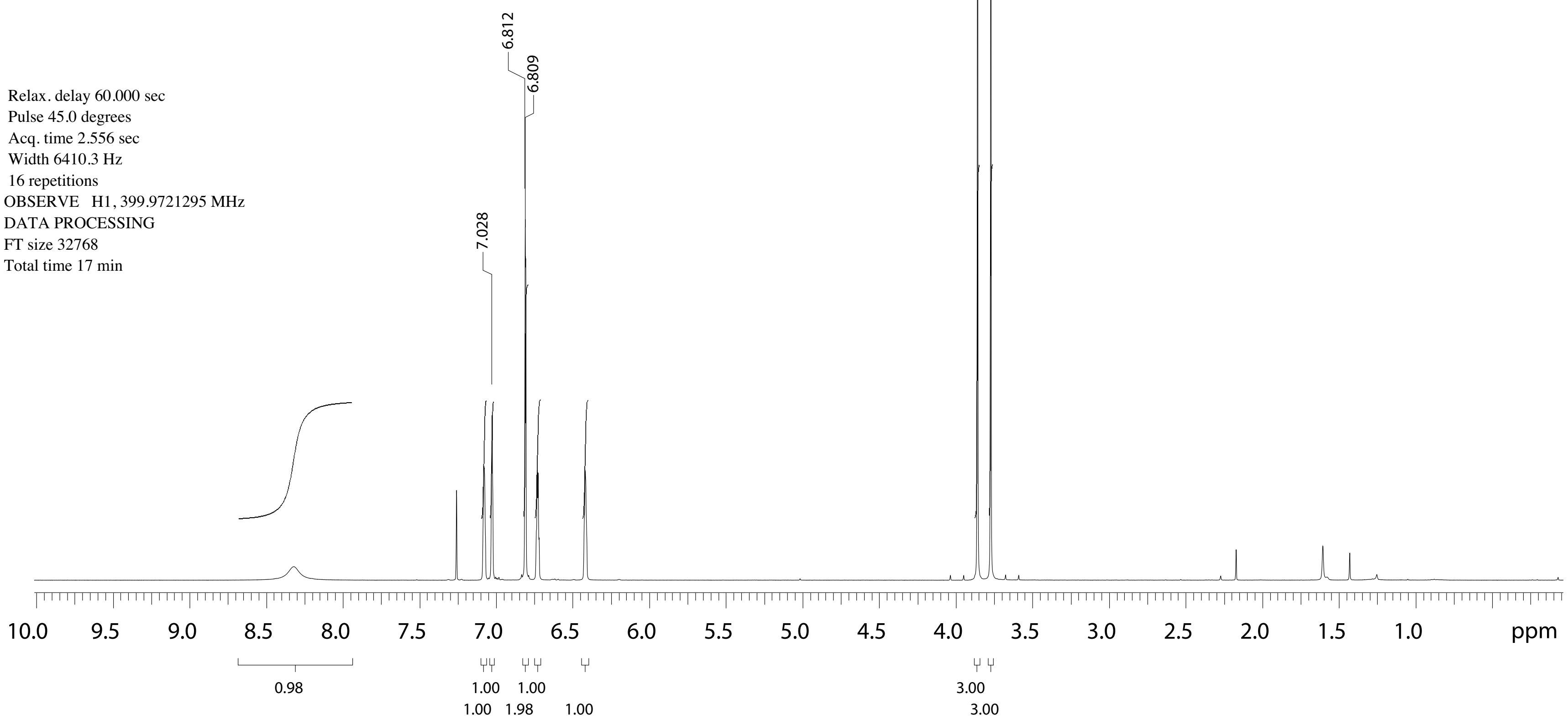

Figure S 25. ^{1}H NMR spectrum (400 MHz, $CDCl_3$ solution) of **3d**.

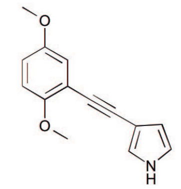

3d

Relax. delay 1.000 sec
Pulse 45.0 degrees
Acq. time 1.311 sec
Width 25000.0 Hz
2000 repetitions
OBSERVE C13, 100.5730722 MHz
DECOUPLE H1, 399.9741302 MHz
Power 35 dB
continuously on
WALTZ-16 modulated
DATA PROCESSING
Line broadening 0.5 Hz
FT size 65536
Total time 1 hr, 17 min

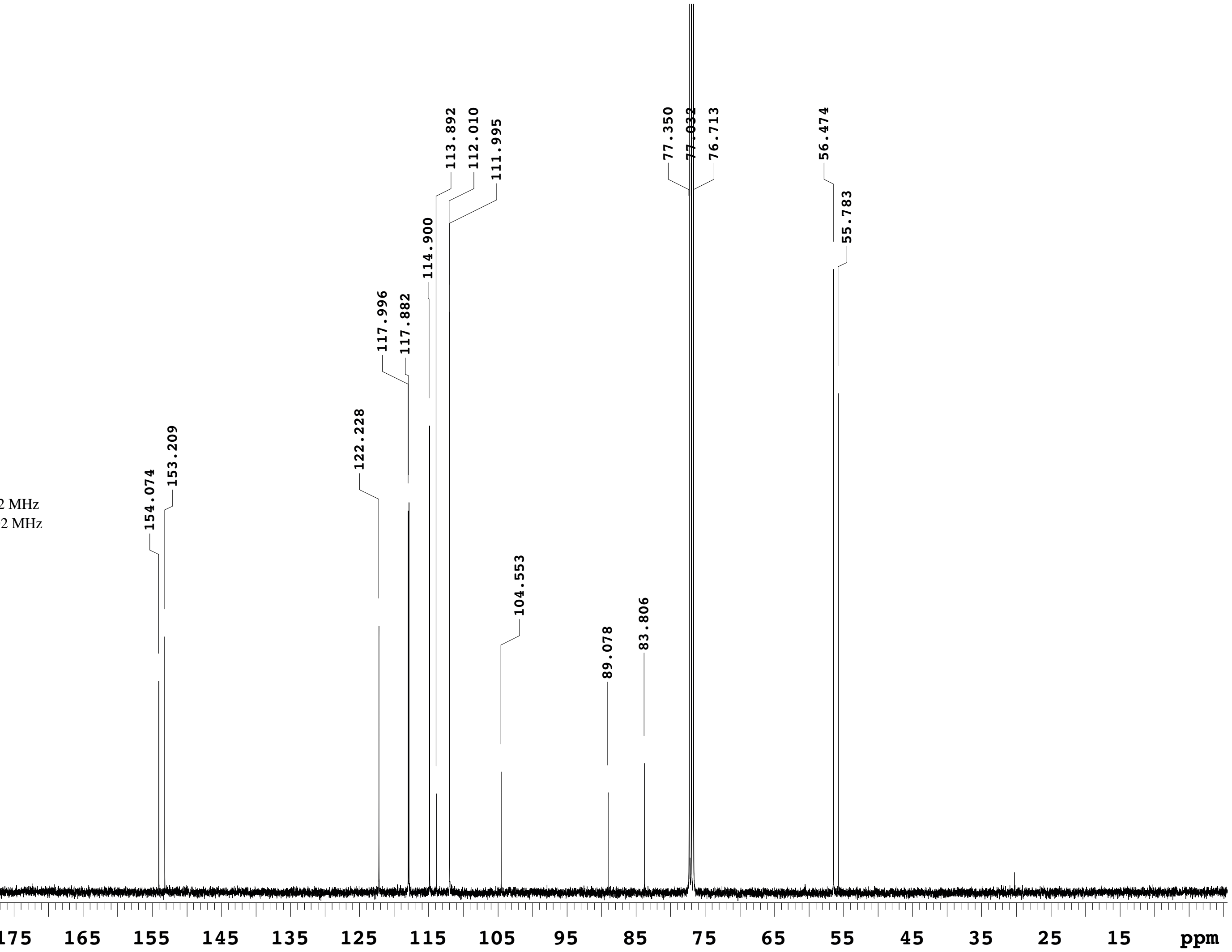

Figure S 26. ^{13}C NMR spectrum (100.6 MHz, $CDCl_3$ solution) of **3d**.

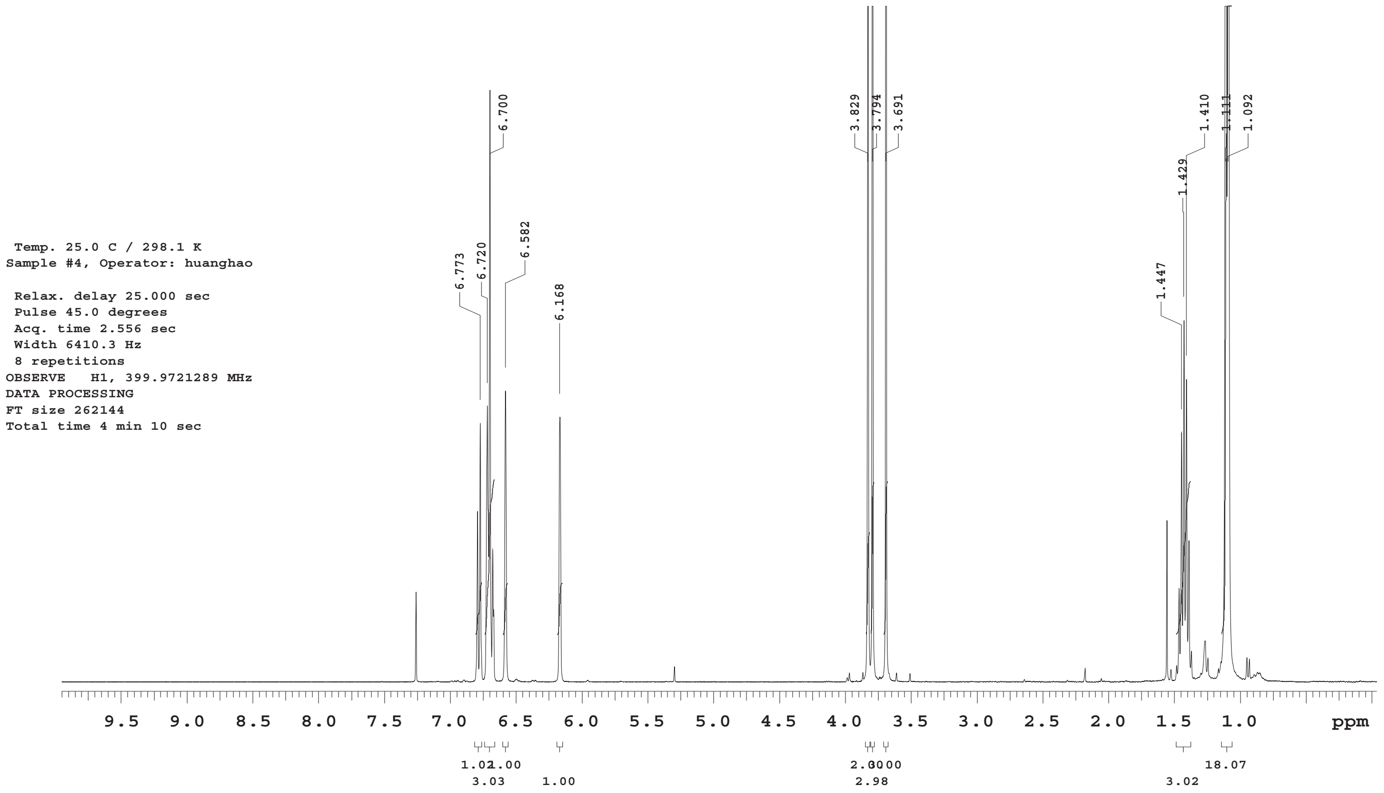

Figure S 27. ^{1}H NMR spectrum (400 MHz, $CDCl_3$ solution) of **4a**.

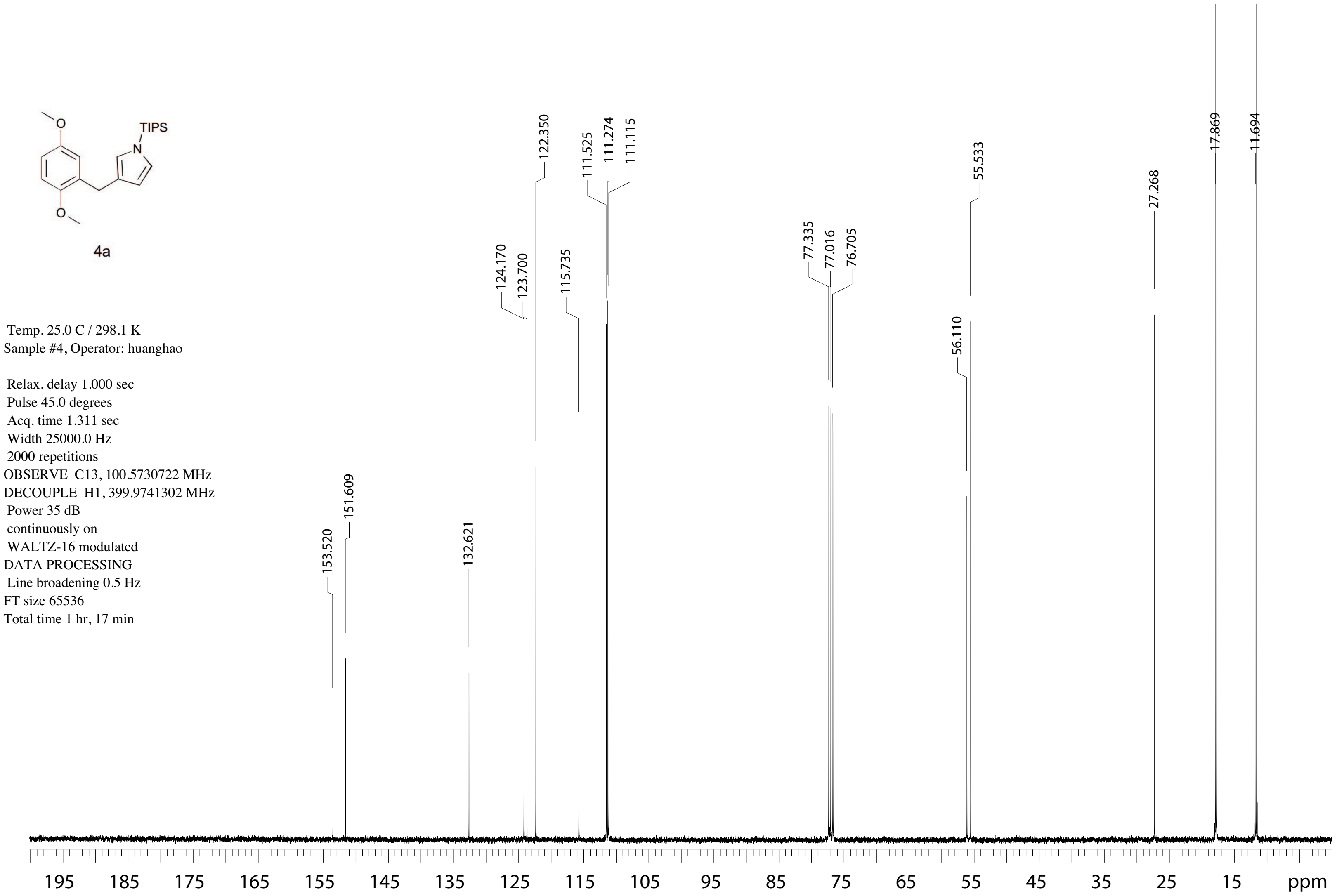

Figure S 28. ^{13}C NMR spectrum (100.6 MHz, $CDCl_3$ solution) of **4a**.

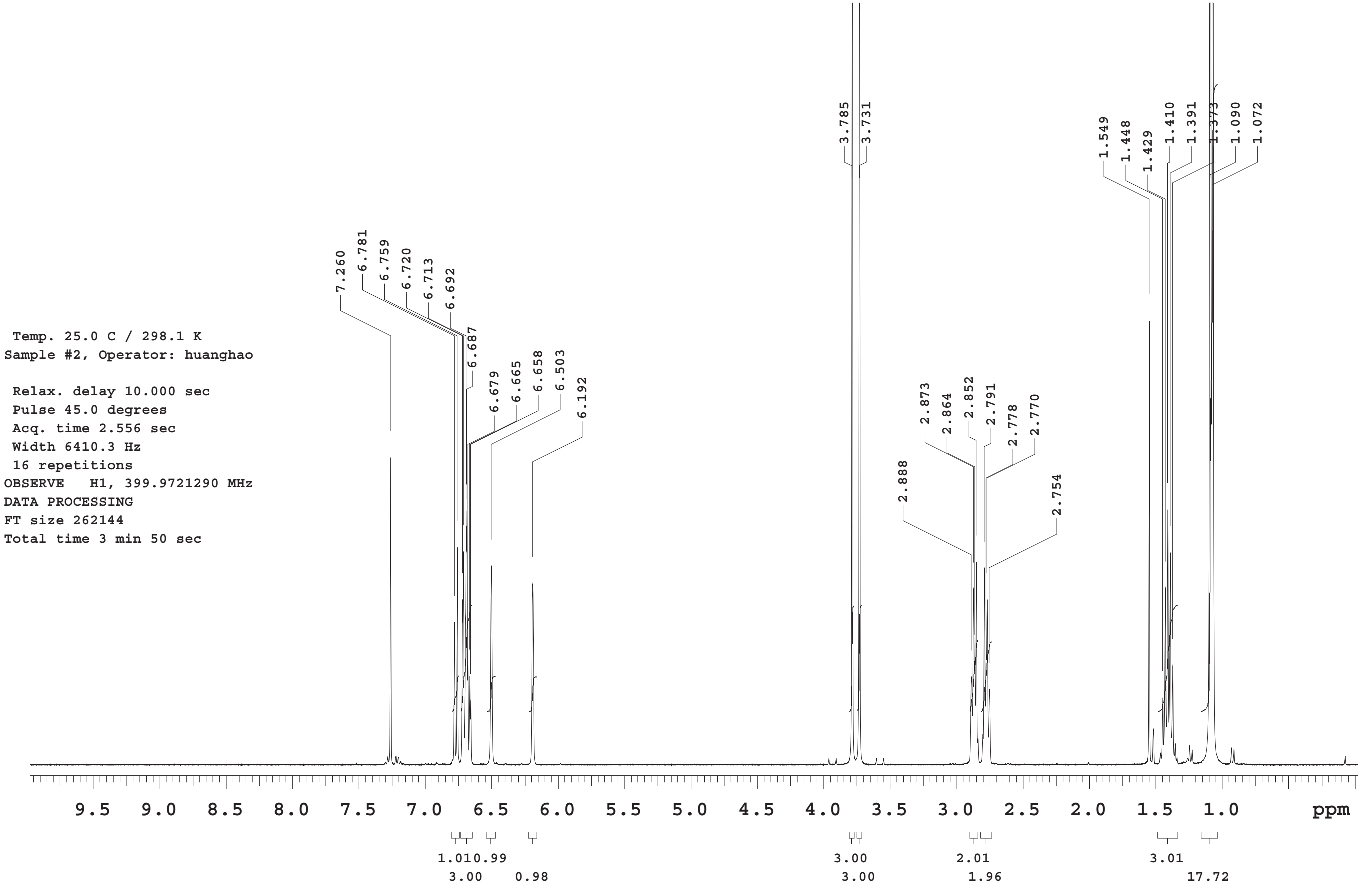

Figure S 29. ^{1}H NMR spectrum (400 MHz, $CDCl_3$ solution) of **4b**.

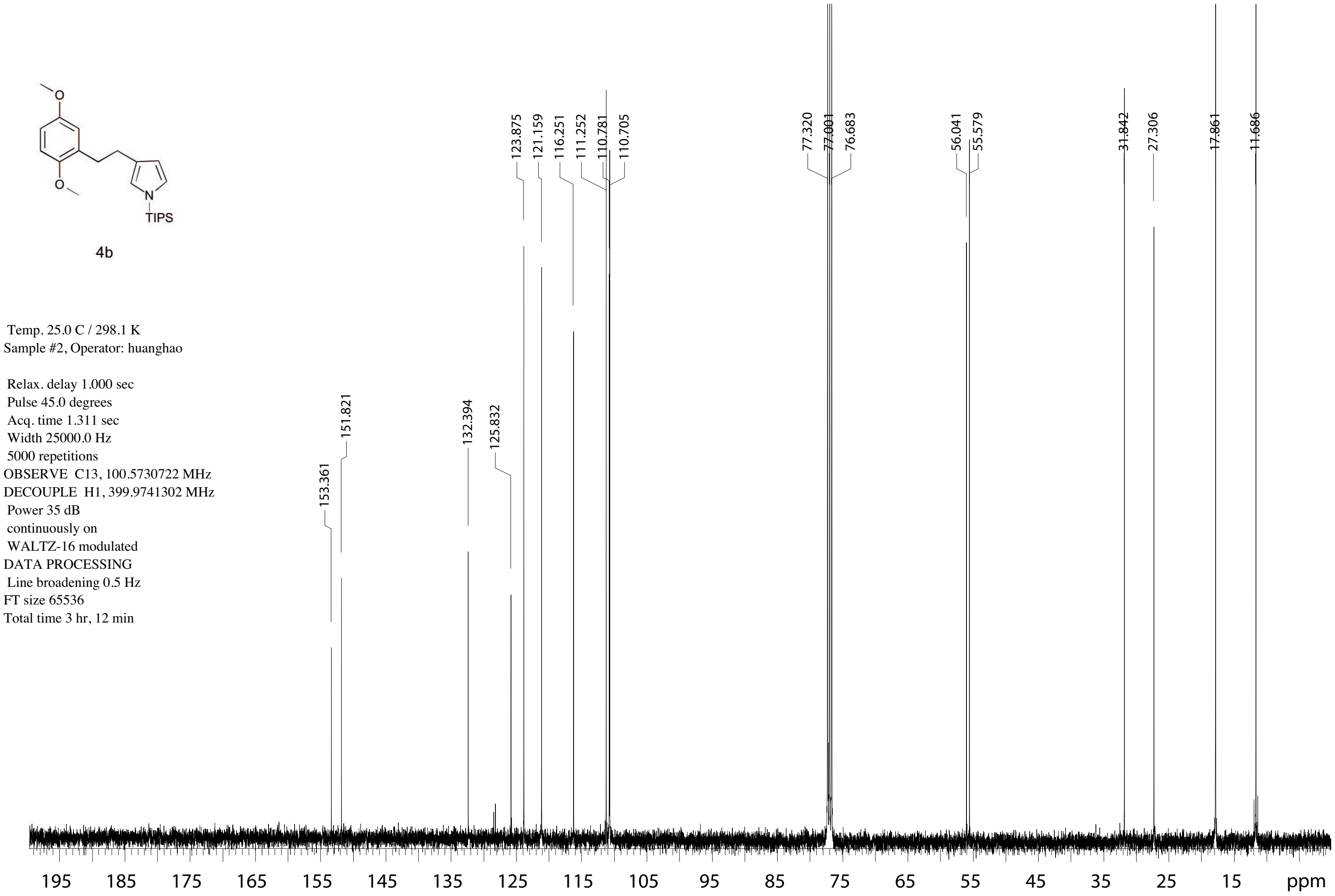

Figure S 30. ^{13}C NMR spectrum (100.6 MHz, $CDCl_3$ solution) of **4b**.

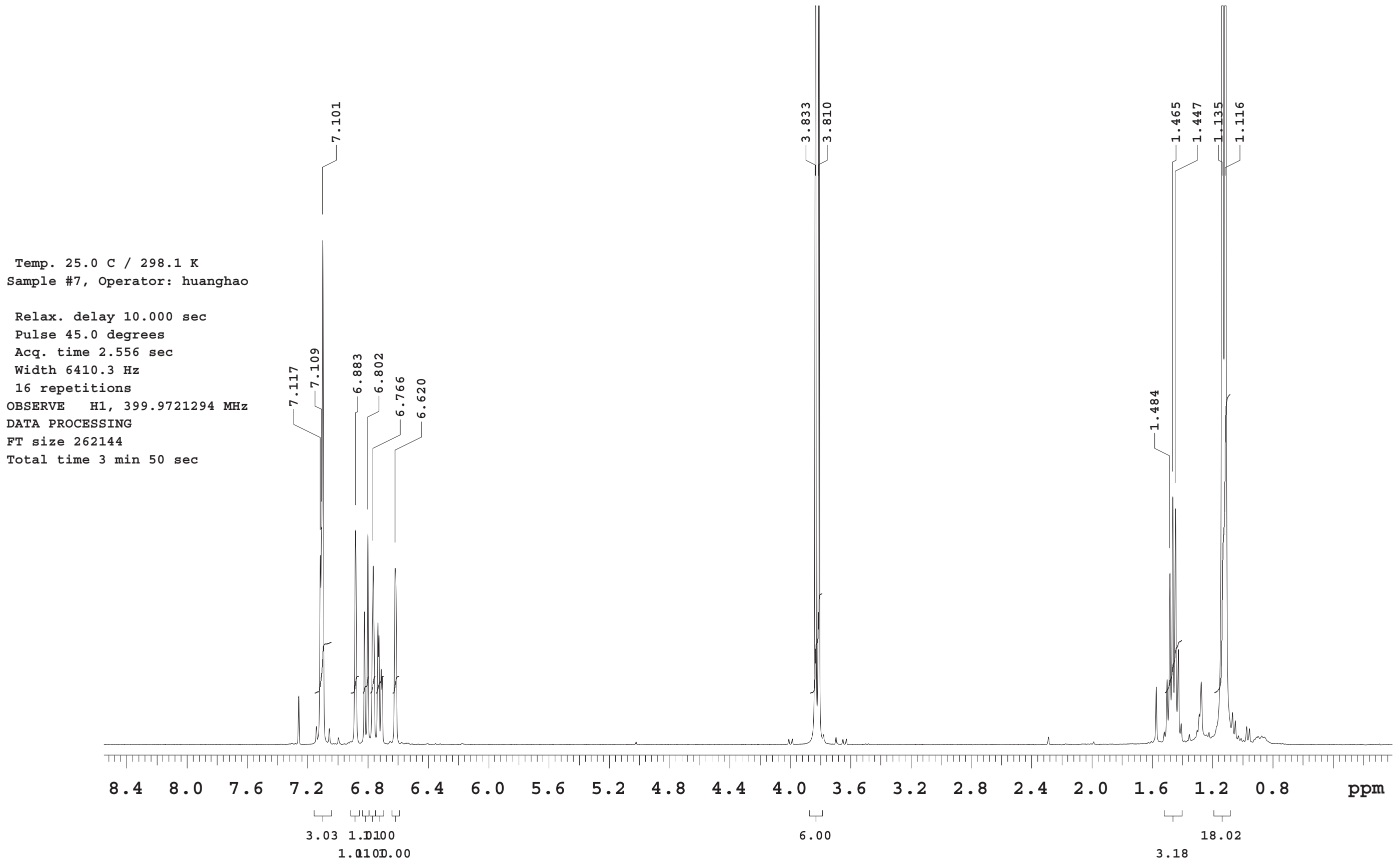

Figure S 31. ^{1}H NMR spectrum (400 MHz, $CDCl_3$ solution) of *trans*-**4c**.

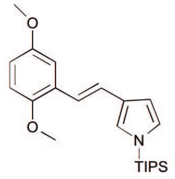

trans-4c

Temp. 25.0 C / 298.1 K
Sample #7, Operator: huanghao

Relax. delay 1.000 sec
Pulse 45.0 degrees
Acq. time 1.311 sec
Width 25000.0 Hz
2000 repetitions
OBSERVE C13, 100.5730722 MHz
DECOUPLE H1, 399.9741302 MHz
Power 35 dB
continuously on
WALTZ-16 modulated
DATA PROCESSING
Line broadening 0.5 Hz
FT size 65536
Total time 1 hr, 17 min

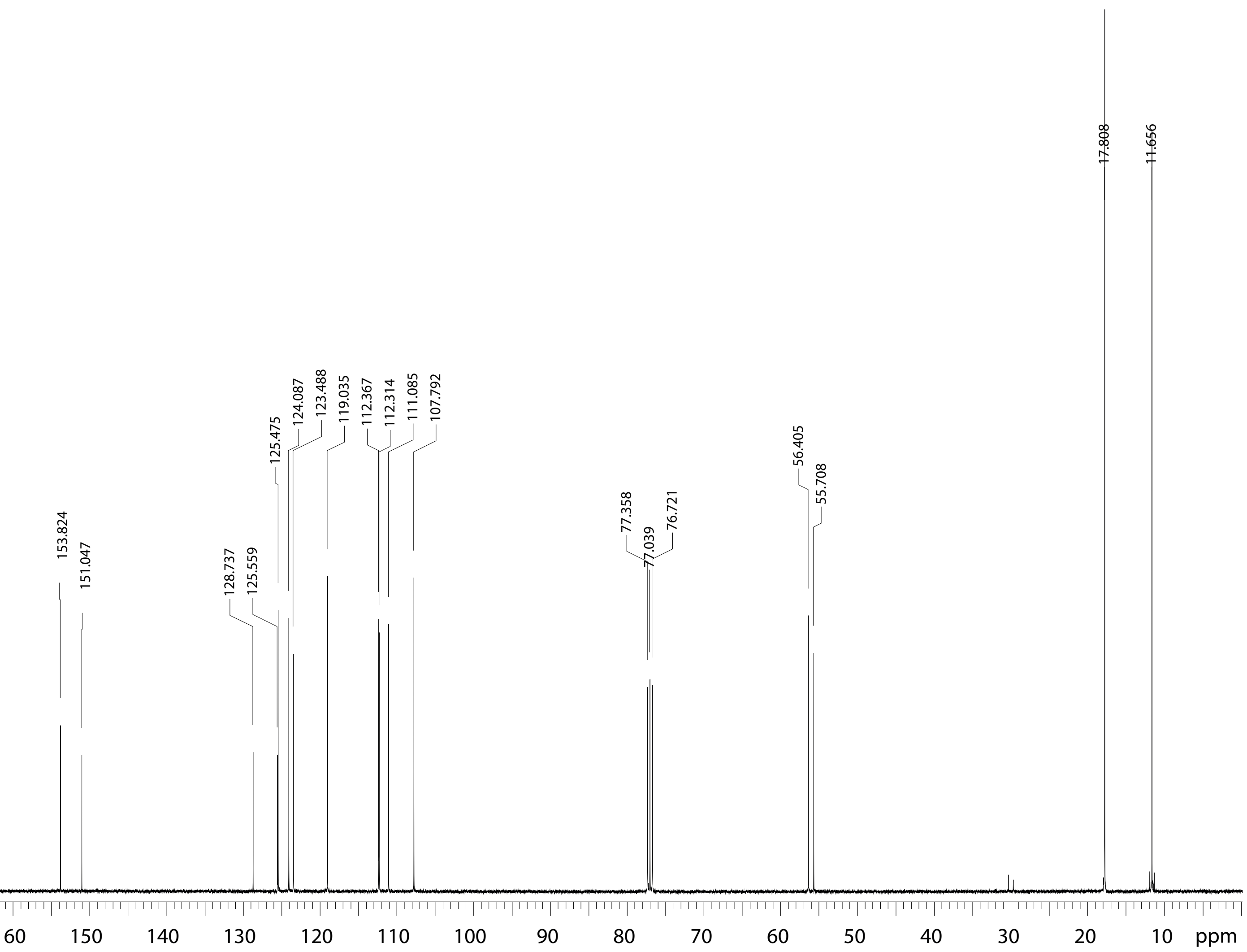

Figure S 32. ^{13}C NMR spectrum (100.6 MHz, $CDCl_3$ solution) of *trans*-**4c**.

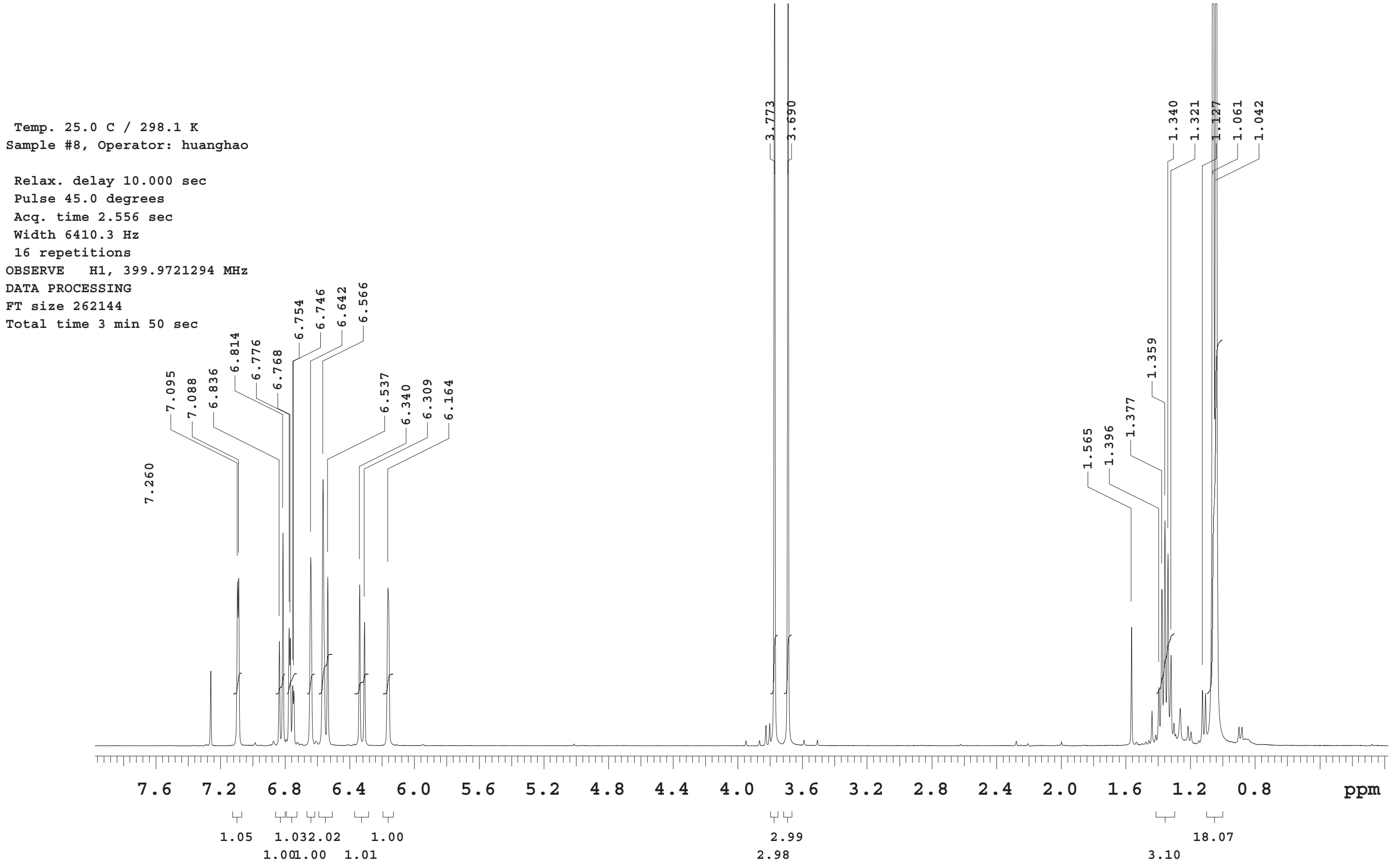

Figure S 33. ¹H NMR spectrum (400 MHz, $CDCl_3$ solution) of *cis*-**4c**.

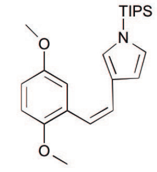

cis - **4c**

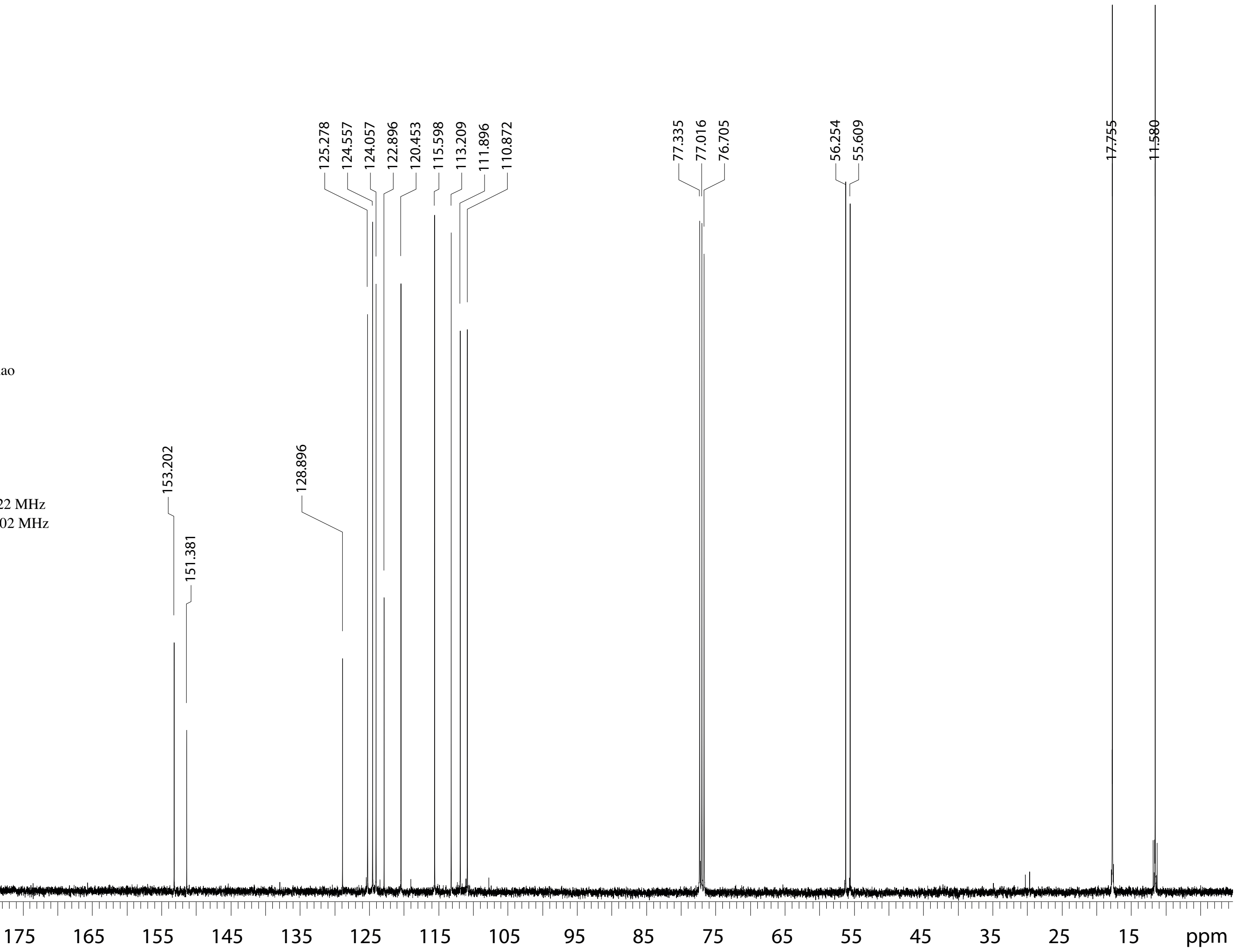

Figure S 34. ^{13}C NMR spectrum (100.6 MHz, $CDCl_3$ solution) of *cis*-**4c**.

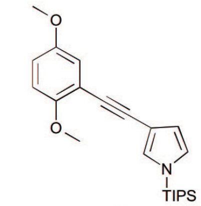

4d

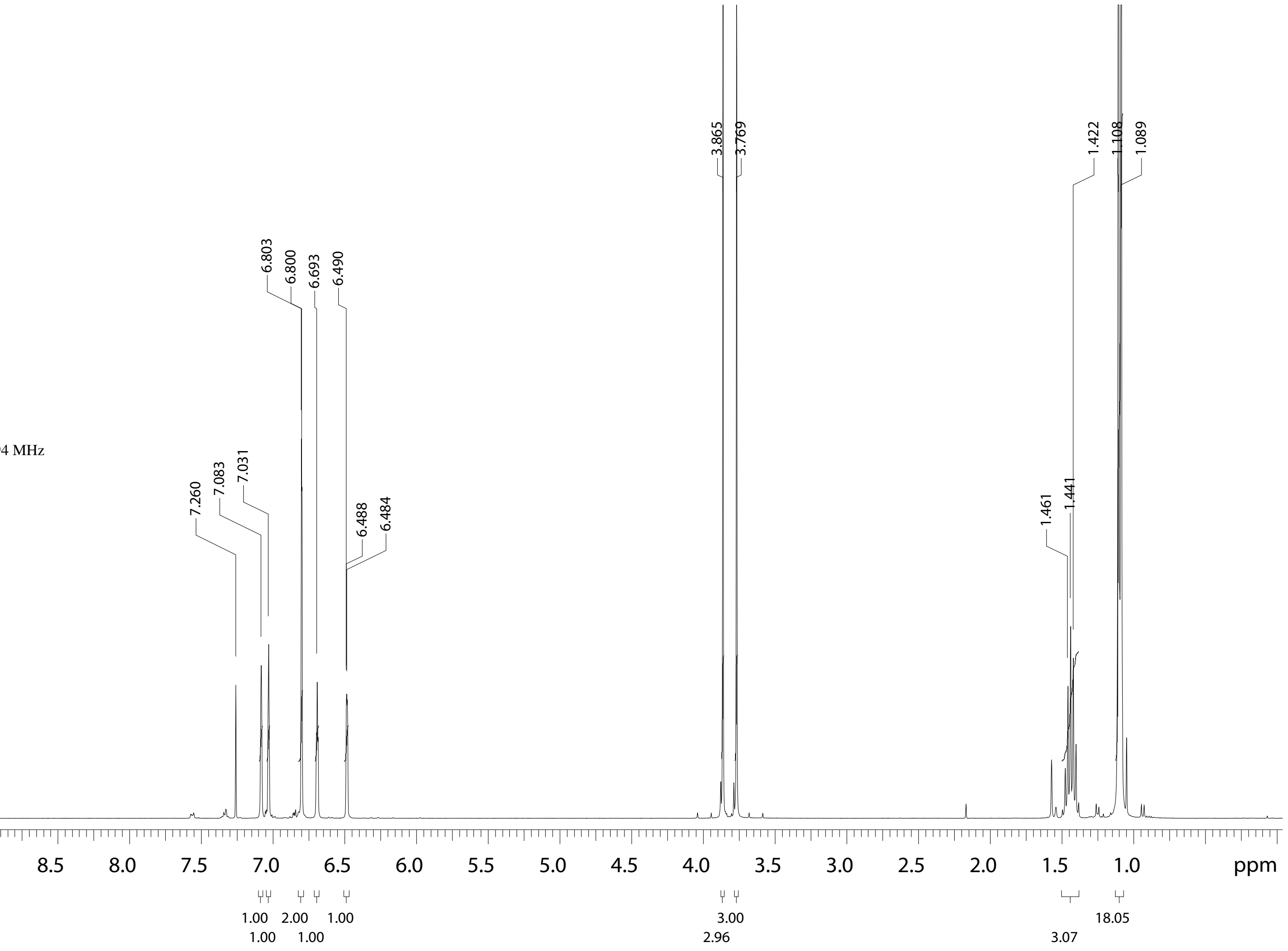

Figure S 35. ^{1}H NMR spectrum (400 MHz, $CDCl_3$ solution) of **4d**.

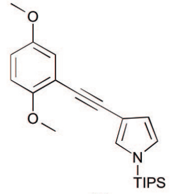

4d

Relax. delay 1.000 sec
Pulse 45.0 degrees
Acq. time 1.311 sec
Width 25000.0 Hz
2000 repetitions
OBSERVE C13, 100.5730722 MHz
DECOUPLE H1, 399.9741302 MHz
Power 35 dB
continuously on
WALTZ-16 modulated
DATA PROCESSING
Line broadening 0.5 Hz
FT size 65536
Total time 1 hr, 17 min

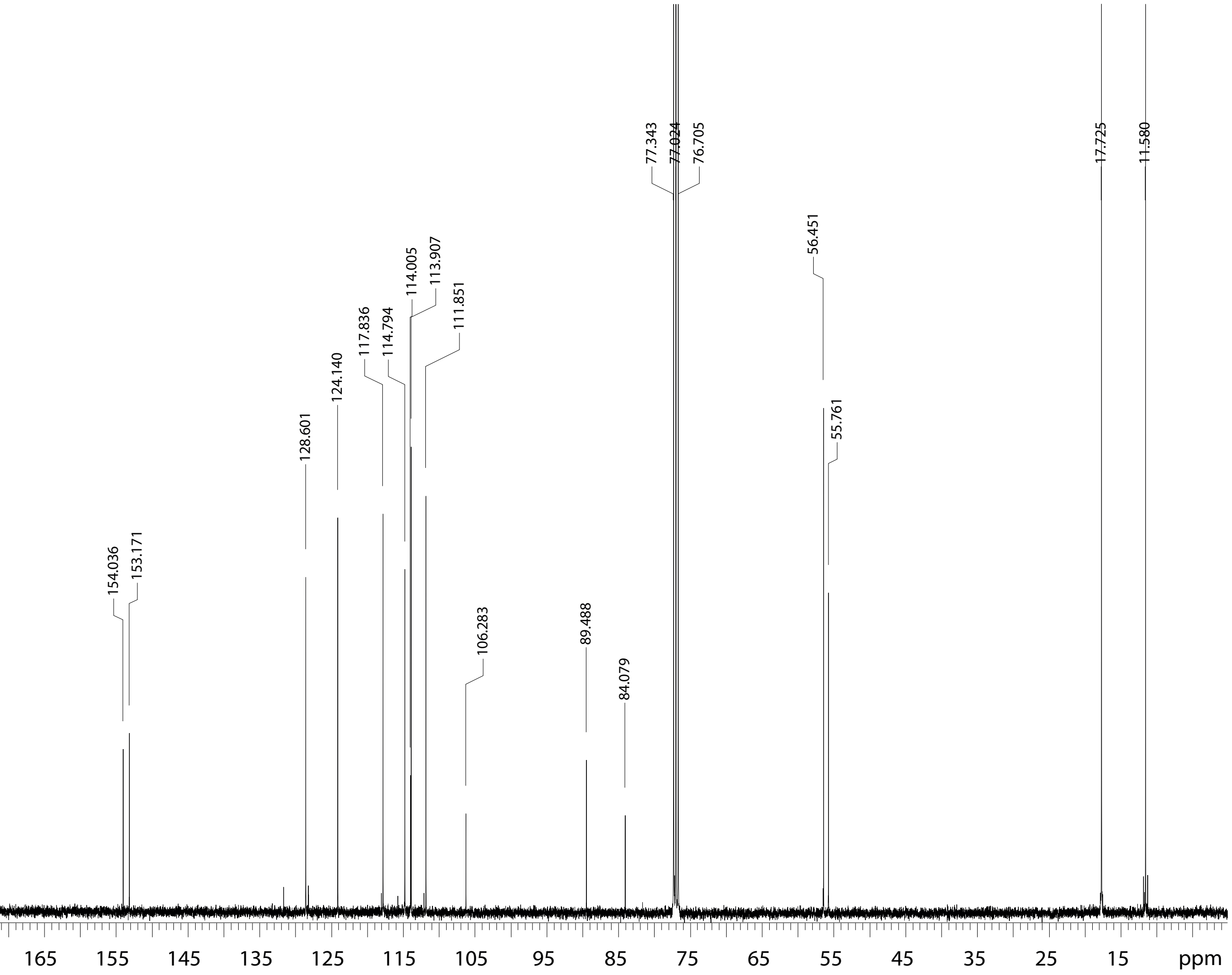

Figure S 36. ^{13}C NMR spectrum (100.6 MHz, $CDCl_3$ solution) of **4d**.

Mass spectra

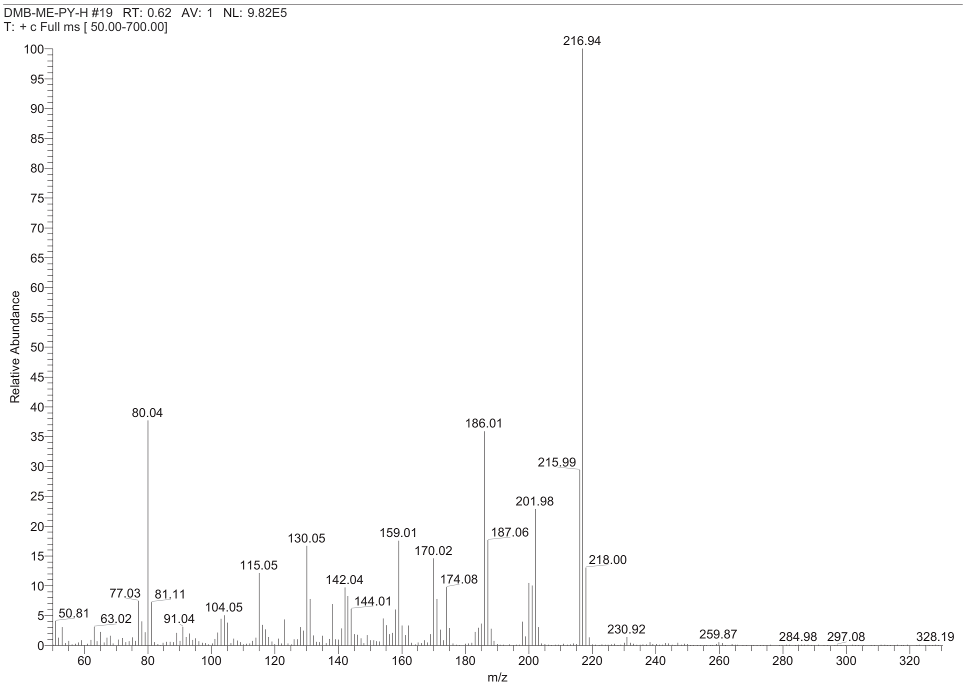

Figure S 37. Mass spectrum of **3a**.

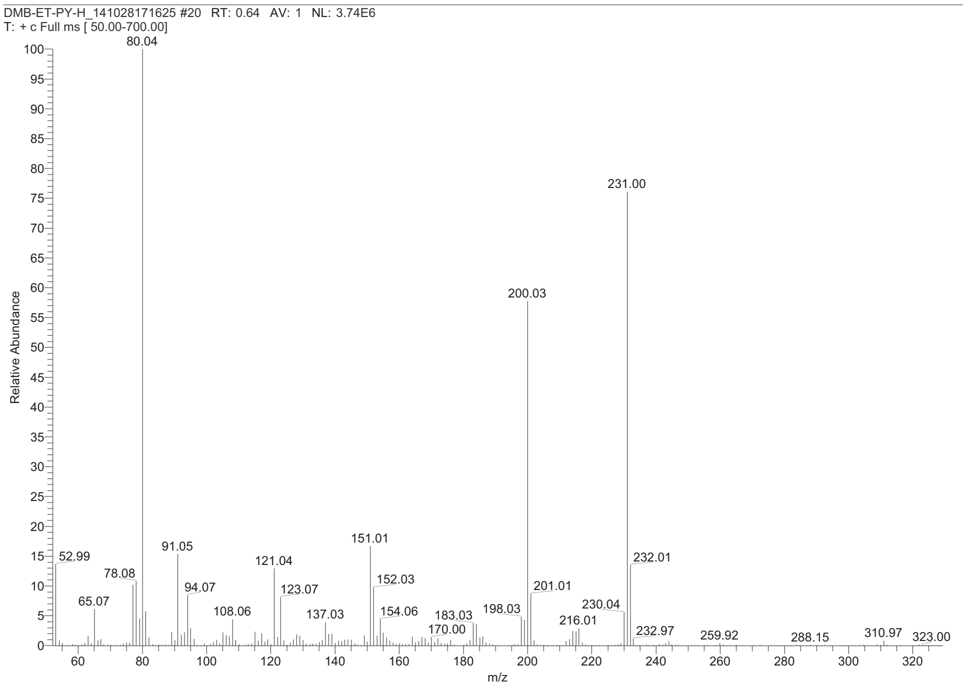

Figure S 38. Mass spectrum of **3b**.

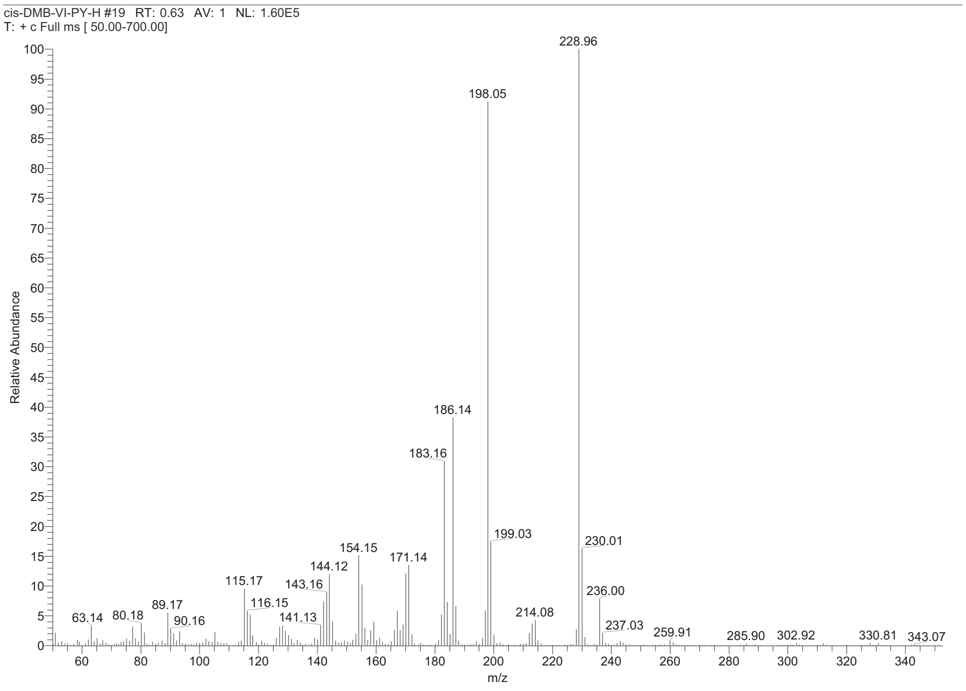

Figure S 39. Mass spectrum of *cis*-**3c**.

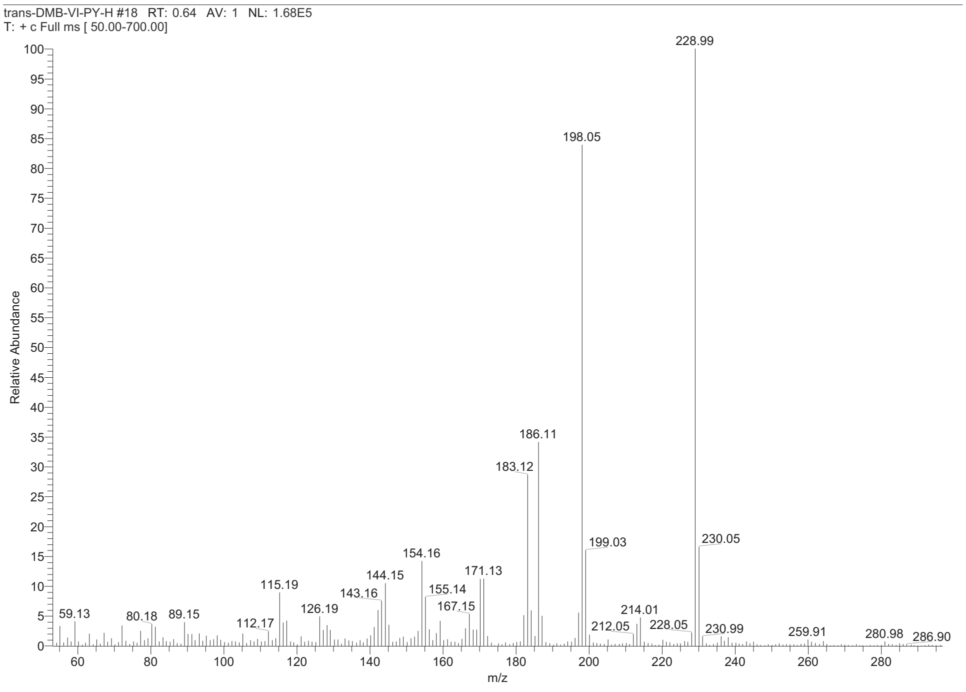

Figure S 40. Mass spectrum of *trans*-**3c**.

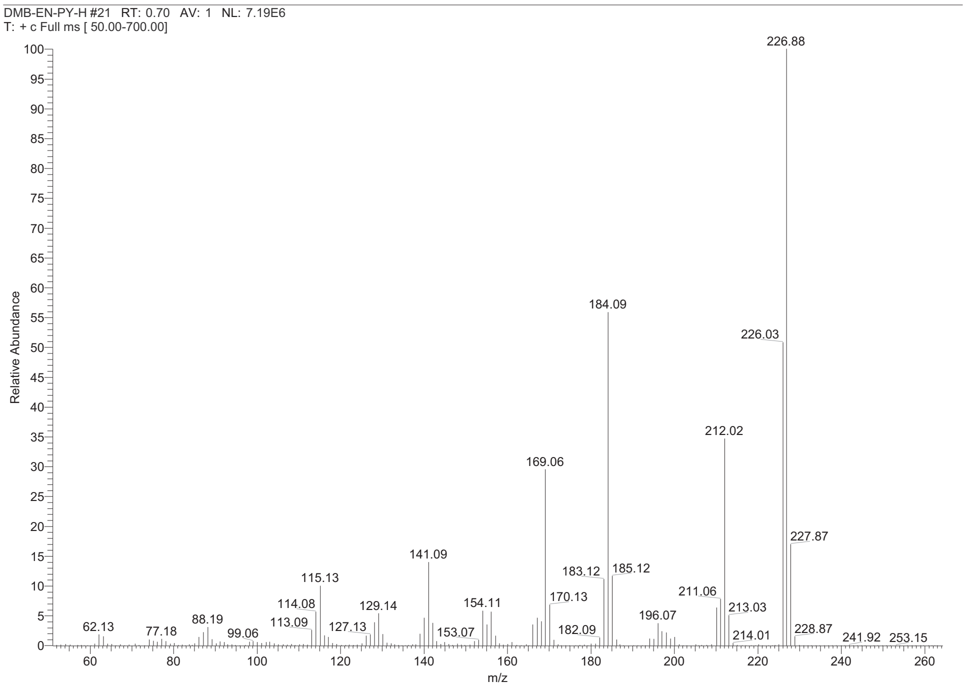

Figure S 41. Mass spectrum of **3d**.

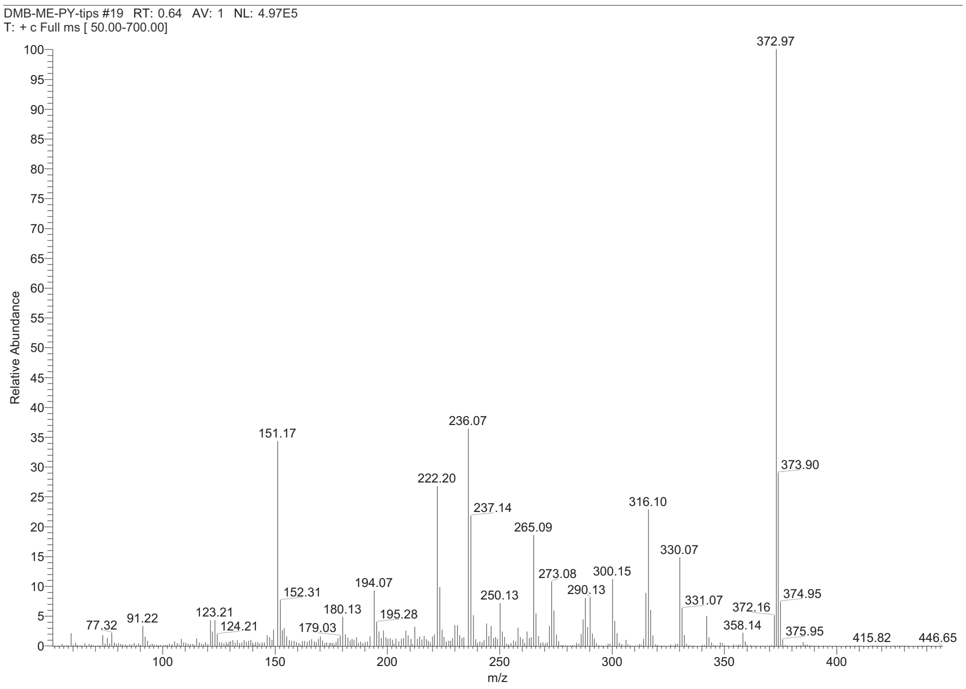

Figure S 42. Mass spectrum of **4a**.

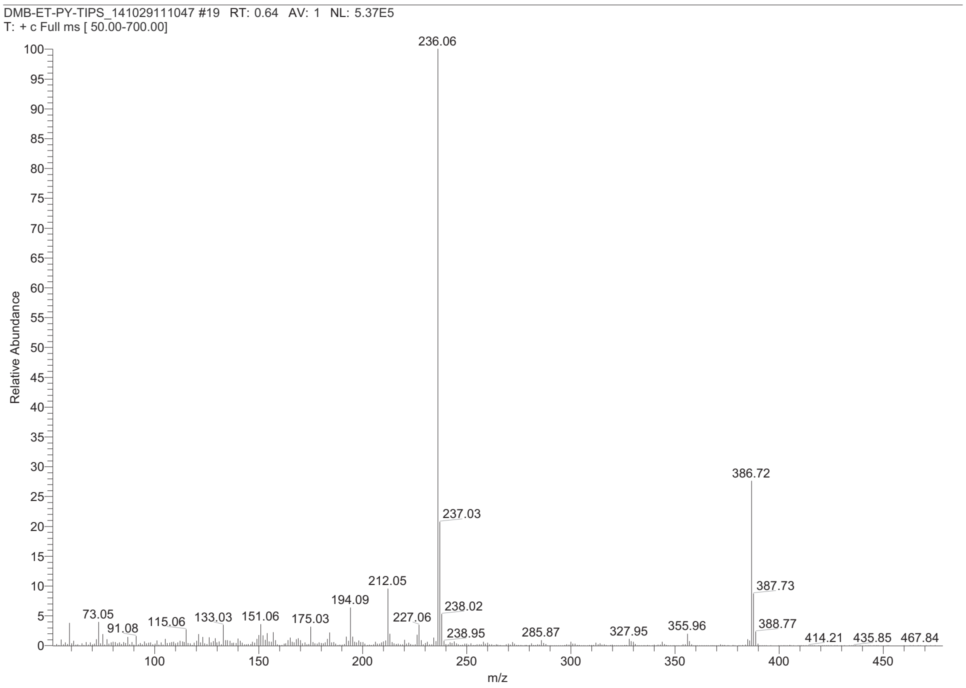

Figure S 43. Mass spectrum of **4b**.

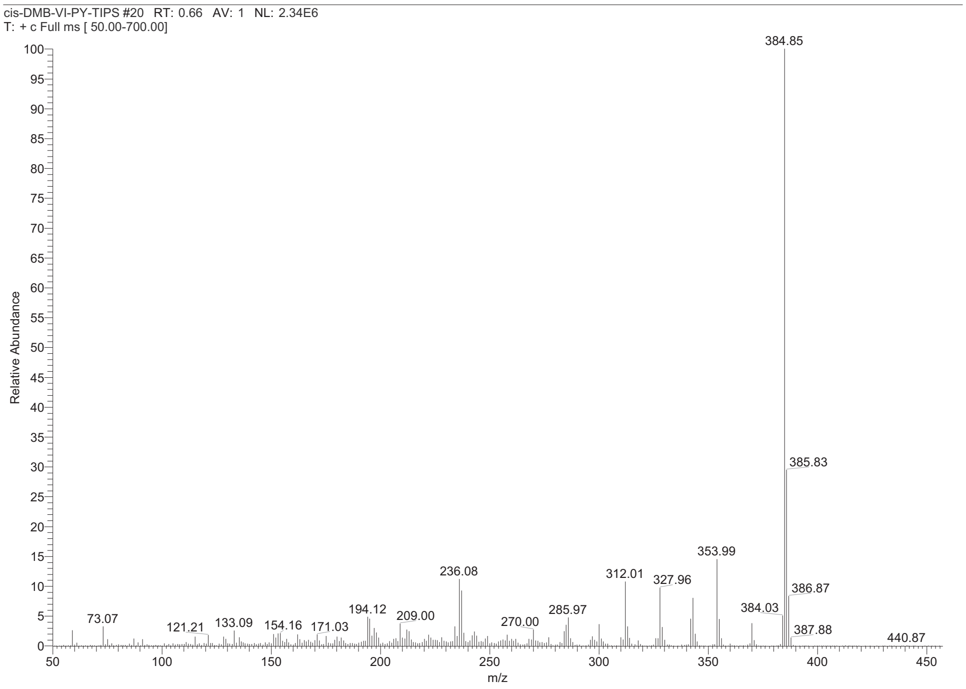

Figure S 44. Mass spectrum of *cis*-**4c**.

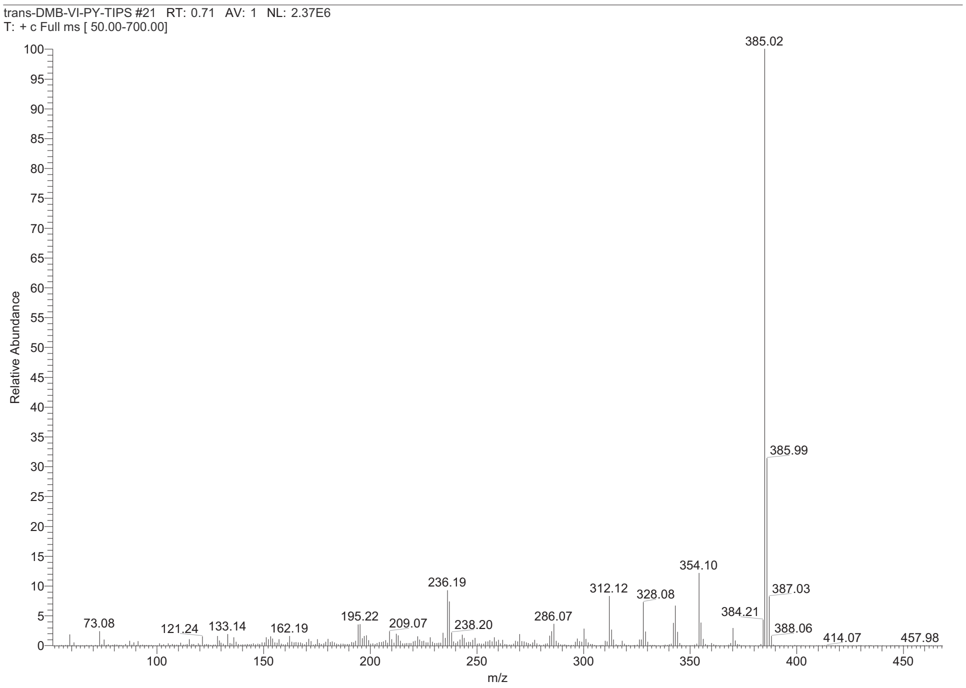

Figure S 45. Mass spectrum of *trans*-**4c**.

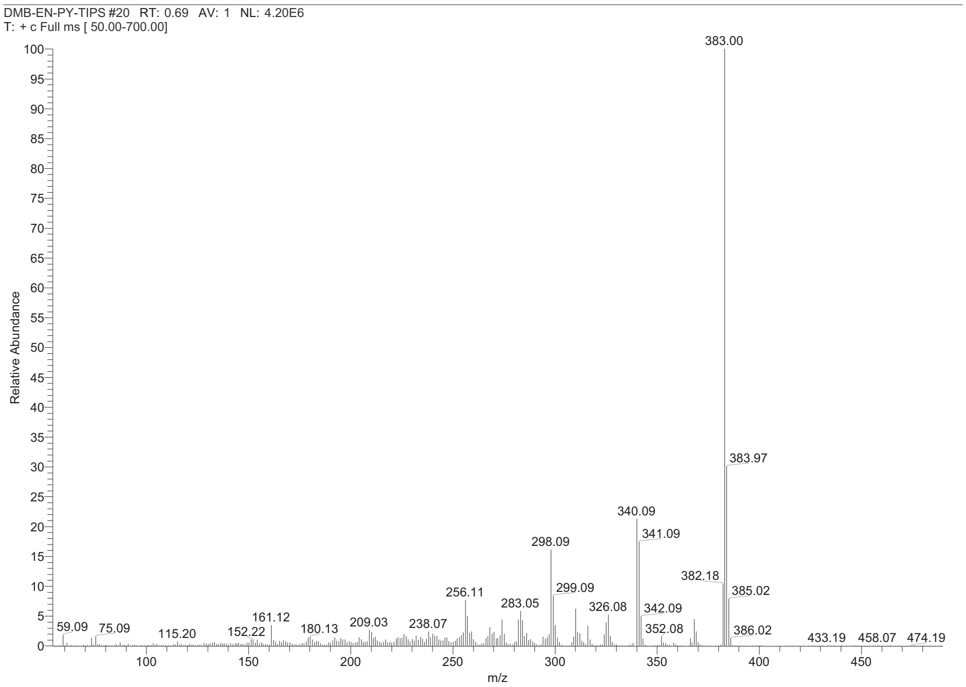

Figure S 46. Mass spectrum of **4d**.

Calculated NMR chemical shifts

Table S 2. Comparison of experimental and calculated ^{1}H chemical shifts for the dyads discussed in the text.

		Py-2	Py-4	Py-5	Ph-3	Ph-4	Ph-6
**1**	δ_{exp}	7.36	6.62	6.83	6.87	6.70	7.13
	δ_{DFT}	7.30	6.55	6.73	6.75	6.61	7.08
	$Error_{abs}$	0.06	0.07	0.10	0.12	0.09	0.05
**3a**	δ_{exp}	6.57	6.12	6.72	6.78	6.69	6.76
	δ_{DFT}	6.61	6.20	6.49	6.65	6.66	6.57
	$Error_{abs}$	0.04	0.08	0.23	0.13	0.03	0.19
**3b**	δ_{exp}	6.63	6.2	6.76	6.84	6.76	6.84
	δ_{DFT}	6.59	5.99	6.68	6.67	6.67	6.62
	$Error_{abs}$	0.04	0.21	0.08	0.17	0.09	0.22
trans-**3c**	δ_{exp}	6.91	6.52	6.78	6.81	6.72	7.10
	δ_{DFT}	6.68	6.52	6.70	6.75	6.62	6.63
	$Error_{abs}$	0.23	0.00	0.08	0.06	0.10	0.47
cis-**3c**	δ_{exp}	6.72	6.09	6.60	6.84	6.78	7.1
	δ_{DFT}	6.68	5.86	6.44	6.64	6.78	6.61
	$Error_{abs}$	0.04	0.23	0.16	0.20	0.00	0.49
**3d**	δ_{exp}	7.08	6.42	6.73	6.8	6.8	7.03
	δ_{DFT}	7.04	6.32	6.61	6.65	6.75	6.79
	$Error_{abs}$	0.04	0.10	0.12	0.15	0.05	0.24

The average error is 0.13 ppm and the RMS value is 0.17 ppm.

Table S 3. Comparison of experimental and calculated ^{13}C chemical shifts for the dyads discussed in the text.

		Py-2	Py-3	Py-4	Py-5	Ph-1	Ph-2	Ph-3	Ph-4	Ph-5	Ph-6
**1**	δ_{exp}	118.1	120.2	107.9	117.7	125.6	150.6	112.3	110.6	153.7	114.0
	δ_{DFT}	118.8	121.6	105.4	115.9	125.2	150.1	109.4	112.4	154.4	106.0
	$Error_{abs}$	0.7	1.4	2.4	1.9	0.4	0.5	2.9	1.8	0.6	7.9
**3a**	δ_{exp}	115.9	122.2	109.2	117.8	132.3	151.5	111.2	110.8	153.5	116.4
	δ_{DFT}	112.8	127.1	107.1	116.5	133.5	150.4	108.9	112.9	153.2	110.0
	$Error_{abs}$	3.1	4.9	2.1	1.3	1.3	1.1	2.3	2.2	0.2	6.4
**3b**	δ_{exp}	115.0	124.1	108.6	117.6	132.3	151.8	111.3	110.8	153.4	116.2
	δ_{DFT}	111.8	126.4	107.0	116.2	132.2	151.1	108.5	113.8	153.6	110.1
	$Error_{abs}$	3.1	2.3	1.6	1.4	0.1	0.8	2.8	2.9	0.2	6.1
trans **-3c**	δ_{exp}	117.4	123.5	105.9	119.0	128.5	151.0	112.4	112.4	153.8	111.1
	δ_{DFT}	118.3	124.9	102.5	118.2	126.9	152.4	109.8	112.9	153.5	111.4
	$Error_{abs}$	0.8	1.3	3.4	0.8	1.6	1.4	2.6	0.5	0.3	0.2
cis- **3c**	δ_{exp}	118.6	120.9	108.9	117.8	128.7	151.4	112.0	113.4	153.2	115.5
	δ_{DFT}	119.2	124.2	106.5	116.6	129.9	150.7	108.8	114.3	153.1	109.4
	$Error_{abs}$	0.7	3.3	2.4	1.2	1.2	0.7	3.2	1.0	0.1	6.2
**3d**	δ_{exp}	122.2	104.5	112.0	118.0	113.9	154.1	112.0	114.9	153.2	117.9
	δ_{DFT}	123.0	105.4	111.5	116.8	113.3	154.2	108.9	115.7	153.1	112.3
	$Error_{abs}$	0.8	0.9	0.5	1.2	0.6	0.2	3.1	0.9	0.1	5.5

The average error is 1.9 ppm, and the RMS is 2.6 ppm.

Gaussian 09 was used to perform the DFT calculation at B3LYP level of theory. Structures were optimized in the gas phase with the 6-311+G(d) basis set. NMR chemical shifts were calculated by the GIAO method with 6-311+G(2d,p) at the B3LYP level of theory using the SDM solvent model with chloroform as solvent.[1] NMR chemical shift data were extracted from the computed isotropic shielding constants and the slope and intercept from the linear regression analysis (eq 1):

$$\delta = \frac{A - \sigma}{-B} \qquad \text{(eq. 1)}$$

Here, σ is the isotropic shielding constant, A is the intercept, and B is the slope. The values of intercept and slope were obtained from reference 1.

1. Lodewyk, M. W.; Siebert, M. R.; Tantillo, D. J., *Chem. Rev.* **2012,** *112* (3), 1839-1862.
